# Synergistic Combination of NAPROC-13 and NMR ^13^C DFT Calculations: A Powerful Approach for Revising the Structure of Natural Products

**DOI:** 10.1021/acs.jnatprod.3c00437

**Published:** 2023-09-07

**Authors:** Hugo A. Sánchez-Martínez, Juan A. Morán-Pinzón, Esther del Olmo Fernández, David López Eguiluz, José F. Adserias Vistué, José L. López-Pérez, Estela Guerrero De León

**Affiliations:** † CIPFAR, Departamento de Farmacología Facultad de Medicina, University of Panama, Avenue Octavio Mendez Pereira, Panamá City 0801, Panamá; ‡ Departamento de Ciencias Farmacéuticas, Área de Química Farmacéutica, Facultad de Farmacia, CIETUS, IBSAL. Campus Miguel de Unamuno, University of Salamanca, 37007 Salamanca, Spain; § Departamento de Sistemas, Fundación General, University of Salamanca, Fonseca 2, 37002 Salamanca, Spain

## Abstract

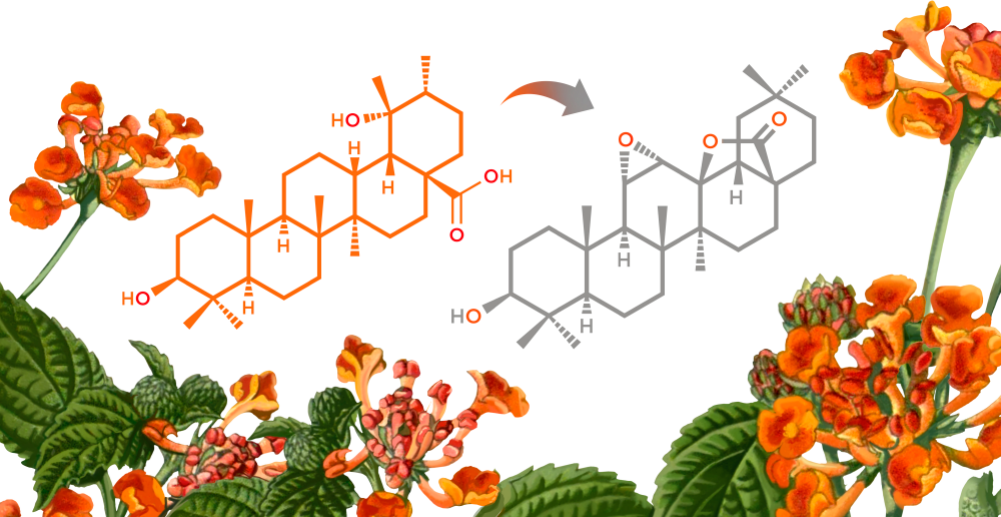

This article describes the structure revision of nine triterpenoids that have been reported corresponding to the same ^13^C NMR data set. In addition, ^13^C NMR calculation shows that some chemical shift assignments must be swapped. Our analysis improves the fit between the experimental and calculated data. Correcting misassigned structures and correctly assigning each signal is essential for elucidating new structurally related compounds. Furthermore, the ambiguity of several compounds, the structure of which differs in the literature and the Sci-Finder database, has been eliminated. Misassigned structures were found by chemical shift searches in NAPROC-13, and the results provide two or more different compounds with the same ^13^C NMR data. The process to determine the correct, most likely structural proposal in agreement with the experimental ^13^C NMR data was carried out by DFT calculations.

Approximately 3–9% of natural products have wrong structures assigned.^1−4^ In other cases, the structure is probably correct, but one or more NMR signals are misassigned, which is seen relatively frequently in the literature. These errors usually occur when the sample contains impurities, signals are neglected due to their low intensity, or the multiplicity of some ^13^C NMR signals is misinterpreted. In addition, an uncorrected typographical mistake could occur during the writing process. NAPROC-13^5^ is a web-based application that contains ^13^C NMR spectroscopic information for more than 26,000 natural products. More than 6% of the entries in NAPROC-13 are corrections to publications that contain structural errors. These corrections were incorporated after appearing in articles involving structural revisions or because an error or typo was noted as the entries were introduced into the database.

This article describes the structure revision of nine triterpenoids that were published with different structures corresponding to the same ^13^C NMR data set. The misassigned structures were found by searches in NAPROC-13 for chemical shifts, and the results provided two or more different compounds with the same ^13^C NMR data. DFT calculations were used to establish the correct or most likely structure proposal, in agreement with the ^13^C NMR experimental data. The herein methodology used for structural revisions is different from the many and varied computer-assisted structure elucidation (CASE) methods available.^6^ To demonstrate the usefulness of our approach, a comparative study was performed for the structure revision of two misassigned straight-chain natural polyenols, **A1** and **B1** (Figure 1). In one case, the study was supported by a CASE method in combination with the DFT-parametric computational method DU8+.^7^ In the other case, the study was supported by the method described in this publication. For further details, see the Supporting Information. The misassigned proposals and the correct structures are structurally distant. Thus, despite the structural simplicity of both compounds **A2** and **B2**, solving these structures is undoubtedly difficult (Figure 1). Furthermore, performing structural revision is even more difficult because the molecular ion deduced from the mass spectrum of **A1** and the number of carbons deduced from the molecular formula of **B1** are incorrect.

**Figure 1:**
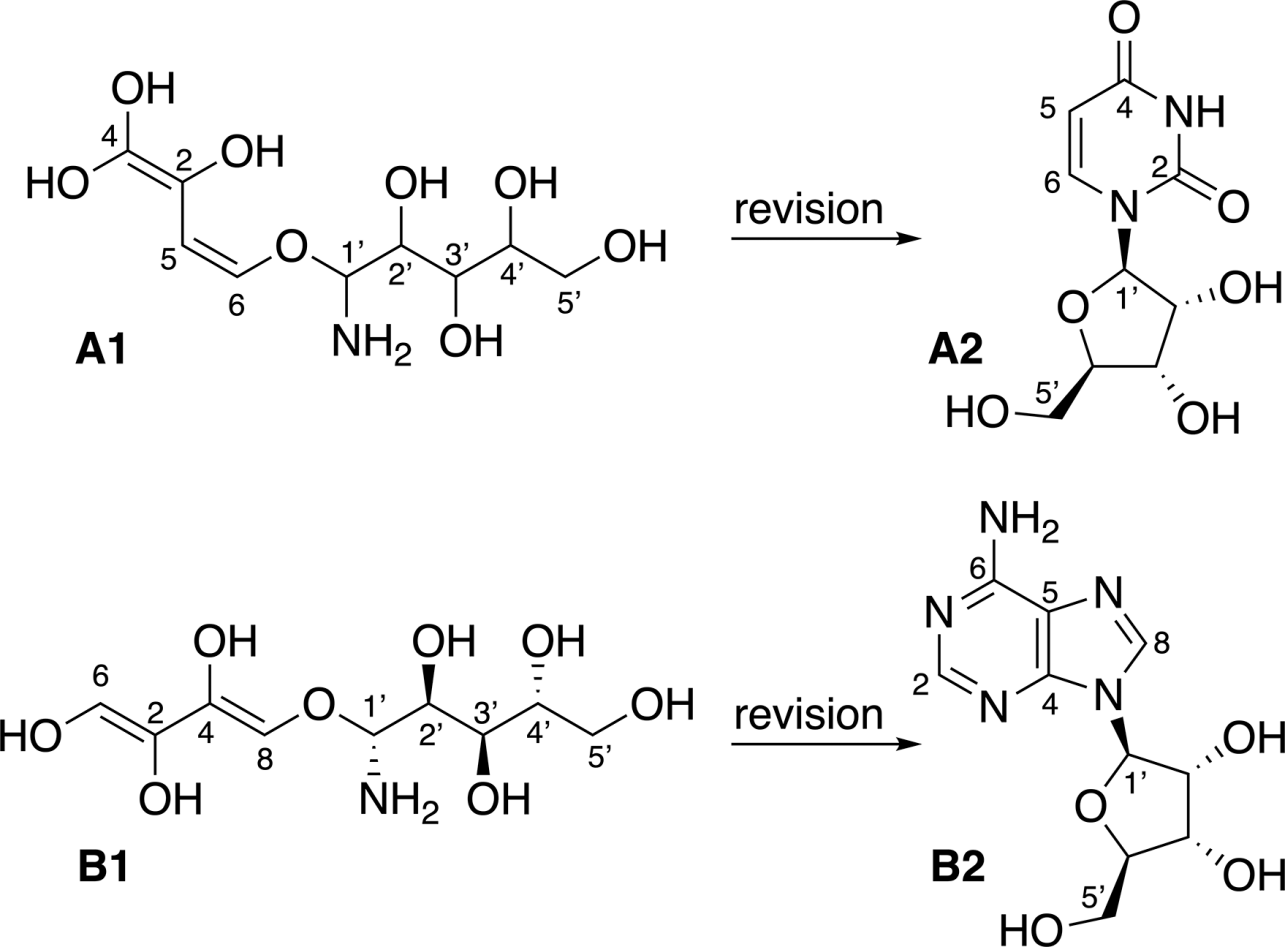
Misassigned structures of (5S,6R,7S,8R)-5-amino-(2Z,4Z)-1,2,3-trihydroxybuta-2,4-dienyloxypentane-6,7,8,9-tetraol (**A1**) and (*Z*)-5-amino-5-(1,1,2-trihydroxybuta-1,3-dienyloxy)pentane-6,7,8,9-tetraol (**B1**) and the revised structures uridine (**A2**) and adenine (**B2**).

Ma et al.^8^ and Siebatcheu et al.^9^ described the structures of compounds **A1** and **B1** (Figure 1). According to Kutatelage et al.,^7^ both compounds contain different enol groups, which is very unlikely based on their stability; thus, the compounds may be misassigned substances. According to our approach for the revision of misassigned compounds, a search by ^13^C NMR chemical shifts was carried out for putative misassigned structures **A1** and **B1** in NAPROC-13. The search results obtained for the **A1** chemical shift provided, in addition to **A1**, uridine (**A2**) and three other compounds (SI: I.1 and I.2). The ^13^C NMR chemical shifts of both substances **A1** and **A2** are almost identical. The structure of uridine is well established and solved by X-ray diffraction.^10^ The search of **B1** chemical shifts yielded five additional compounds (SI: II.1 and II.2). All of them are either adenosine (**B2**) or compounds that have adenosine as a structural fragment. A combined search that also considered the molecular weight deduced from **B1** mass spectra allows us to narrow down the result to only **B1** and **B2** (SI: II.3, II.4, and II.5). All of the carbons of both substances are color-coded according to the deviations between the searched chemical shifts and those of the values of the found compounds except for C-5 of compound **B2**, which resonates at 121 ppm and is not present in **B1**. The availability of the ^13^C NMR spectrum in the SI in both publications that describe this compound allows us to observe the presence of a signal at 121 ppm, which has been neglected by the authors. Consequently, **B1** and **B2** are identical. The structure of adenosine was established by X-ray diffraction.^11^ Therefore, **A1** and **B1** structures could be revised in a matter of minutes using NAPROC-13.

The structure revision of nine triterpenoids found in NAPROC-13 is discussed below, for which the application of established CASE methods may not be productive due to the size of the molecule and multiple possibilities of functional location, which generates a considerable number of alternatives and makes density functional theory (DFT) calculation methods very expensive. In addition, the different conformational possibilities for each of the stereoisomers or regioisomers generated also must be considered.

## Results and Discussion

The misassigned structures of nine triterpenoids with ursane, lupane, and oleane skeletons were computationally revised by calculating their ^13^C NMR spectra. For this purpose, Spartan’20^12^ was chosen, since it has implemented a protocol for calculating NMR chemical shifts in conformationally flexible molecules. These misassigned compounds were detected using NAPROC-13.^5^ In some of the examples presented, the structural revision involved a change in the triterpene skeleton. However, in the majority of cases, these compounds had already been published with the correct structure before. In addition, the differences between some structures in Sci-Finder and those in the original papers have been clarified.

**Figure 2:**
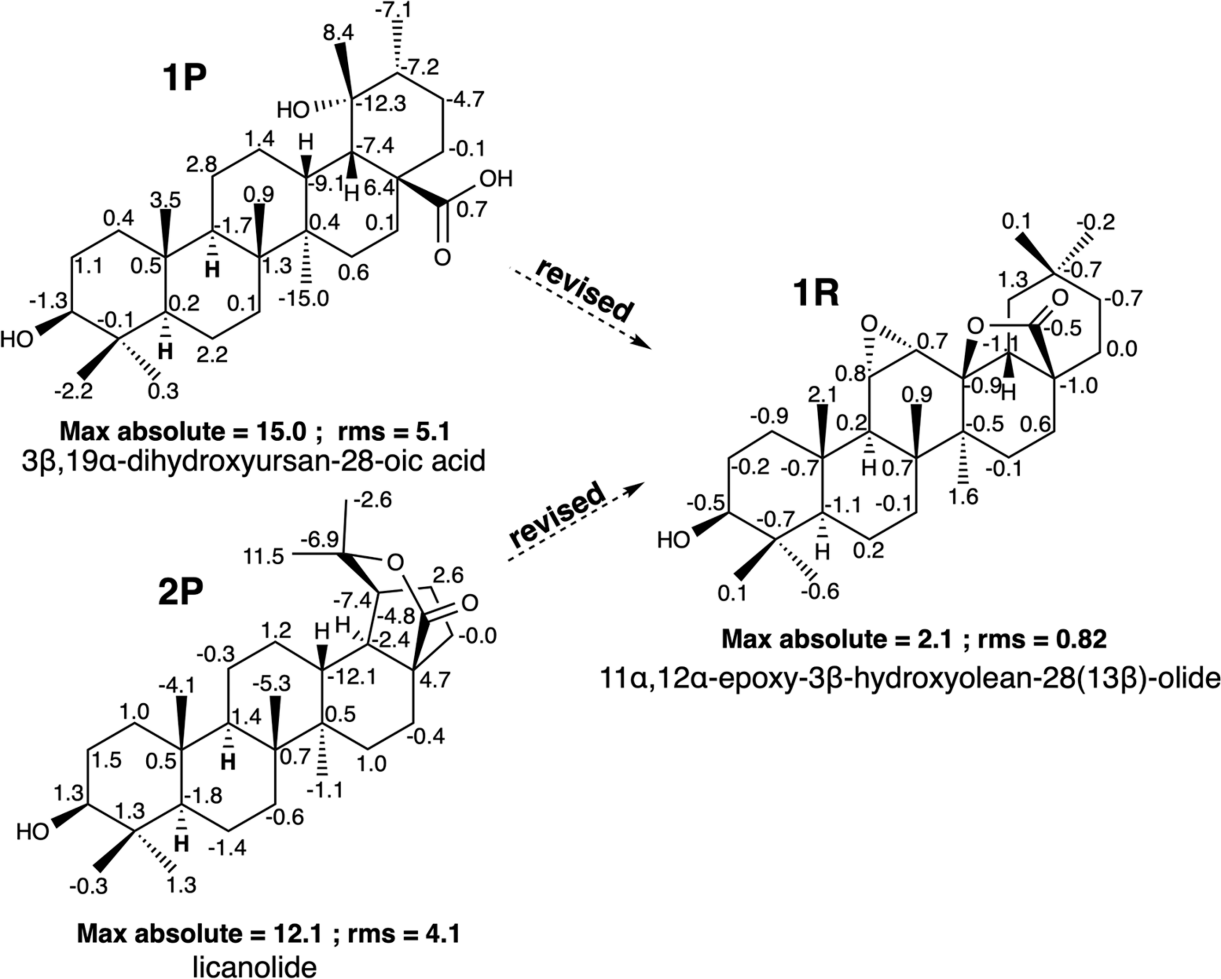
Misassigned structures of 3β,19α-dihydroxyursan-28-oic acid **(1P**) and licanolide (**2P**) and the correct structure of 11α,12α-epoxy-3β-hydroxyolean-28(13β)-olide (**1R**). Negative and positive deviations of δ_C_ (calculated value – experimental value) are indicated for each carbon in the structures. Max *y* rms values are underneath each structure

In a phytochemical study of the roots of *Lantana camara* L. (Verbenaceae),^13^ a new triterpenoid with an ursane skeleton, 3β,19α-dihydroxyursan-28-oic acid (**1P**) (Figure 2), was isolated. Like many other triterpenoids of this group, it contains hydroxy groups at C-3 and C-19 and a carboxylic group at C-28. The distinct structural feature of this triterpene is the absence of a Δ^12^ double bond, which is present in similar compounds of ursane group triterpenoids. Compound **1P** is the only triterpene of the ursane group with the described structural features. These data, together with the value of the chemical shift assigned to Me-27 at δ 33.2 (in other related ursanes, Me-27 resonates at δ 18), caught our attention and prompted us to search for their chemical shifts in NAPROC-13.^5^ Furthermore, the values of the ^13^C NMR shifts of the E-ring carbons of this triterpene greatly differ from those of other ursanes. As a result of the search, in addition to **1P**, two other triterpenoids were found, licanolide [3β-hydroxylupane-20(28)-olide] (**2P**) (Figure 2), a lupane isolated from *Licania tomentosa* (Chrysobalanaceae),^14^ and 11α,12α-epoxy-3β-hydroxyolean-28(13β)-olide (**1R**) (Figure 1), an oleanane isolated from *Paeonia japonica* (Paeoniaceae).^15^ Compound **1R** was previously isolated from *Atractylis carduus* L.^16^ Compounds **1P**, **2P**, and **1R** present very similar ^13^C NMR data (see Table 1 and SI: II.6); however, as seen in Figure 2, their structures are significantly different. The compounds have different carbon skeletons of lupane, ursane, and oleanane types. Moreover, **1P** contains a carboxylic acid and hydroxyl-free groups, while **2P** and **1R** contain a γ-lactone. Compound **1R** also possesses an epoxide group that is not present in the other two compounds. Despite this structural disparity, the reported mass spectra at 70 eV for the three triterpenoids showed identical *m*/*z* values of 470 [M]^+^ for **1P** and **1R** and 471 [M]^+^ for **2P**. In its IR spectra, **2P** shows an absorption band at 872 cm^–1^ characteristic of the epoxide group, similar to the band in the IR spectrum of **1R** (868 cm^–1^). For **1P**, no absorption bands below 1250 cm^–1^ are reported. Therefore, these three products, which have appeared in the literature under different structures, may correspond to the same compound. Determining the relationship between structurally different compounds is relatively straightforward using databases such as NAPROC-13, which offer the ability to search chemical shifts. In their ^1^H NMR spectra, the three compounds show three signals between 3.0 and 3.4 ppm that are only compatible with the **1R** structure. To determine the consistency between the proposed structures for **1P**, **2P**, and **1R** and their corresponding ^13^C NMR spectroscopic data, their ^13^C NMR spectra were computationally calculated following the protocol described in the Experimental Section.^12^ The computational results were compared with the experimental data, and the results are summarized in Figure 2, in which the term max absolute expresses the largest deviations between the calculated and experimental chemical shifts; the statistical term rms is also reported underneath each structure. Negative and positive deviations of δ_C_ (calculated value – experimental value) are indicated for each carbon in the compounds. The results show a good fit between the calculated and experimental values for **1R** (max abs = 2.1, rms = 0.8), while the structural proposals **1P** (max abs = 15.0, rms = 5.1) and **2P** (max abs = 12.1, rms = 4.1) are not compatible with their ^13^C NMR data. Compound **1P** shows very significant deviations between the calculated and experimental values, mainly for C-13 (−9.1), C-18 (−7.4), C-19 (−12.3), and C-27 (−15.0), while **2P** shows the main deviations for C-13 (−12.1), C-19 (−7.4), C-20 (−6.9), and C-29 (11.5). Both proposed structures for **1P** and **2P** were assigned based on two-dimensional NMR analysis. The two-dimensional spectra data for licanolide (**2P**) were reported later by the same authors.^17^ Comparison of the ^13^C NMR signals of **2P** with those of another triterpenoid with the same lupane skeleton, which differs only by free acid and hydroxyl groups instead of lactone, 3β,20-dihydroxylupane-28-oic acid,^18^ shows significant differences between the signals assigned to the E-ring carbons for both substances.

**Table 1:** ^13^C NMR Data for Compounds Revised **1**–**11** Recorded in CDCl_3_ Except **1R** in C_5_D_5_N^a^

δ_C_, type													
C	**1P**(13)	**2P**(14)	**1R**(15)	**3P**(20)	**3R**(21)	**4P**(22)	**4R**(23)	**5P**(24)	**6P**(22)	**7a**(25)	**7b**(26)	**11P**(27)	**11R**(28)
1	38.1, CH_2_	37.5, CH_2_	38.7, CH_2_	38.1, CH_2_	38.1, CH_2_	38.9, CH_2_	38.8, CH_2_	39.1, CH_2_	38.9, CH_2_	22.5, CH_2_	22.5, CH_2_	38.9, CH_2_	38.9, CH_2_
2	26.9, CH_2_	27.8, CH_2_	27.6, CH_2_	23.4, CH_2_	23.4, CH_2_	27.5, CH_2_	27.4, CH_2_	27.6, CH_2_	27.4, CH_2_	41.6, CH_2_	42.6, CH_2_	27.3, CH_2_	27.3, CH_2_
3	78.8, CH	75.8, CH	77.9, CH	80.7, CH	80.6, CH	79.4, CH	79.0, CH	79.2, CH	79.5, CH	213.3, C	213.2, C	78.8, CH	78.9, CH
4	38.8, C	37.2, C	39.4, C	37.9, C	37.9, C	38.8, C	38.9, C	39.1, C	38.8, C	58.4, CH	60.1, CH	40.5, C	38.9, C
5	54.5, CH	49.4, CH	55.0, CH	55.0, CH	54.9, CH	55.4, CH	55.4, CH	55.6, CH	55.4, CH	42.3, C	42.4, C	55.4, CH	55.5, CH
6	17.5, CH_2_	20.9, CH_2_	18.8, CH_2_	17.7, CH_2_	17.9, CH_2_	18.2, CH_2_	18.2, CH_2_	18.5, CH_2_	18.2, CH_2_	41.5, CH_2_	41.7, CH_2_	18.2, CH_2_	18.1, CH_2_
7	34.2, CH_2_	26.4, CH_2_	31.4, CH_2_	31.4, CH_2_	31.2, CH_2_	34.0, CH_2_	33.9, CH_2_	34.2, CH_2_	34.0, CH_2_	18.6, CH_2_	20.8, CH_2_	33.7, CH_2_	33.7, CH_2_
8	41.2, C	40.4, C	40.9, C	41.8, C	41.7, C	40.6, C	40.7, C	40.8, C	40.6, C	53.8, CH	53.1, CH	40.7, C	40.5, C
9	52.7, CH	52.6, CH	49.8, CH	53.0, CH	53.0, CH	50.6, CH	50.6, CH	50.7, CH	50.4, CH	37.6, C	37.6, C	51.2, CH	51.2, CH
10	36.4, C	36.4, C	36.8, C	36.4, C	36.3, C	37.2, C	37.2, C	37.4, C	37.1, C	59.7, CH	58.2, CH	37.2, C	37.2, C
11	21.2, CH_2_	21.6, CH_2_	52.8, CH	133.4, CH	133.2, CH	21.3, CH_2_	21.3, CH_2_	21.3, CH_2_	21.1, CH_2_	37.8, CH_2_	35.6, CH_2_	20.8, CH_2_	26.5, CH_2_
12	31.4, CH_2_	34.1, CH_2_	57.3, CH	129.0, CH	129.0, CH	27.3, CH_2_	27.3, CH_2_	27.6, CH_2_	27.3, CH_2_	24.1, CH_2_	24.2, CH_2_	25.5, CH_2_	20.9, CH_2_
13	50.6, CH	48.0, CH	87.6, C	89.7, C	89.6, C	38.9, CH	38.7, CH	42.5, CH	42.2, CH	45.4, C	42.4, C	35.9, CH	36.0, CH
14	40.5, C	41.3, C	41.7, C	42.0, C	41.9, C	41.6, C	41.6, C	41.5, C	41.2, C	38.4, C	42.5, C	38.8, C	39.9, C
15	29.7, CH_2_	29.5, CH_2_	27.0, CH_2_	25.6, CH_2_	25.6, CH_2_	26.5, CH_2_	26.6, CH_2_	27.1, CH_2_	26.8, CH_2_	32.2, CH_2_	30.3, CH_2_	27.8, CH_2_	27.9, CH_2_
16	26.7, CH_2_	30.3, CH_2_	21.6, CH_2_	30.9, CH_2_	22.8, CH_2_	27.3, CH_2_	27.5, CH_2_	25.7, CH_2_	25.4, CH_2_	36.3, CH_2_	36.2, CH_2_	26.4, CH_2_	31.9, CH_2_
17	43.8, C	43.7, C	44.1, C	45.2, C	45.1, C	37.6, C	37.6, C	48.4, C	48.1, C	30.3, C	30.6, C	46.0, C	33.5, C
18	57.1, CH	50.2, CH	51.2, CH	40.4, CH	60.6, CH	45.4, CH	45.4, CH	47.5, CH	47.3, CH	43.3, CH	43.7, CH	46.6, CH	46.7, CH
19	87.5, C	56.9, CH	38.0, CH_2_	38.2, CH	38.0, CH	44.2, CH	44.2, CH	44.8, CH	44.6, CH	37.1, CH_2_	35.6, CH_2_	85.9, CH	86.0, CH
20	49.6, CH	87.5, C	31.5, C	60.7, CH	40.3, CH	74.2, C	74.2, C	84.0, C	83.7, C	28.5, C	28.5, C	33.5, C	46.1, C
21	31.0, CH_2_	24.8, CH_2_	34.4, CH_2_	22.9, CH_2_	30.8, CH_2_	133.0, CH	133.0, CH	134.0, CH	133.7, CH	32.6, CH_2_	33.1, CH_2_	31.9, CH_2_	32.3, CH_2_
22	37.7, CH_2_	32.5, CH_2_	27.7, CH_2_	31.3, CH_2_	31.4, CH_2_	140.6, CH	140.6, CH	138.7, CH	138.4, CH	40.1, CH_2_	39.3, CH_2_	32.2, CH_2_	25.5, CH_2_
23	27.7, CH_3_	26.8, CH_3_	28.4, CH_3_	27.8, CH_3_	27.9, CH_3_	28.0, CH_3_	28.0, CH_3_	28.2, CH_3_	28.0, CH_3_	7.1, CH_3_	7.0, CH_3_	23.9, CH_3_	27.9, CH_3_
24	18.8, CH_3_	17.0, CH_3_	16.0, CH_3_	16.1, CH_3_	16.0, CH_3_	15.3, CH_3_	15.4, CH_3_	15.6, CH_3_	15.3, CH_3_	14.9, CH_3_	14.7, CH_3_	13.6, CH_3_	15.3, CH_3_
25	15.1, CH_3_	23.4, CH_3_	17.3, CH_3_	19.2, CH_3_	16.0, CH_3_	16.3, CH_3_	16.3, CH_3_	15.9, CH_3_	16.2, CH_3_	18.2, CH_3_	19.8, CH_3_	25.4, CH_3_	16.5, CH_3_
26	17.2, CH_3_	21.1, CH_3_	18.9, CH_3_	19.0, CH_3_	18.9, CH_3_	15.7, CH_3_	15.7, CH_3_	16.5, CH_3_	15.7, CH_3_	22.3, CH_3_	17.9, CH_3_	15.3, CH_3_	15.5, CH_3_
27	33.2, CH_3_	17.3, CH_3_	20.4, CH_3_	16.2, CH_3_	17.8, CH_3_	14.3, CH_3_	14.3, CH_3_	14.3, CH_3_	14.1, CH_3_	63.4, CH_2_	64.3, CH_2_	16.4, CH_3_	13.7, CH_3_
28	179.4, C	179.3, C	178.9, C	18.1, CH_3_	179.7, C	65.9, CH_2_	65.9, CH_2_	175.8, C	175.5, C	32.8, CH_3_	32.1, CH_3_	179.8, C	179.9, C
29	23.6, CH_3_	33.0, CH_3_	33.0, CH_3_	17.9, CH_3_	17.6, CH_3_	21.9, CH_3_	20.9, CH_3_	19.9, CH_3_	19.7, CH_3_	35.8, CH_3_	34.7, CH_3_	28.7, CH_3_	23.9, CH_3_
30	20.0, CH_3_	18.9, CH_3_	23.4, CH_3_	179.9, C	19.1, CH_3_	22.2, CH_3_	22.2, CH_3_	21.3, CH_3_	21.0, CH_3_	30.6, CH_3_	31.9, CH_3_	27.9, CH_3_	28.7, CH_3_
				171.1, C	170.9, C								
				21.4, CH_3_	21.4, CH_3_								

a The chemical shifts correspond to those given in the publications describing each product. After the computational calculation, some chemical shifts were swapped (see Supporting Information).

The ^13^C NMR spectra of the three substances (Table 1 and SI: II.6) show the same number of methyls and methines; however, **1R** shows a quaternary carbon at 31.5 ppm, while **1P** and **2P** show a methylene, which also resonates at a similar chemical shift of 31 ppm (Table 1 and SI: II.6). The small differences observed in the remaining signals can be justified due to the use of different deuterated solvents in NMR experiments (CDCl_3_ for **1P** and **2P** and C_5_D_5_N for **1R**). Most likely, the higher purity of sample **1R** prevented its solubilization in CDCl_3_ and was decisive for the correct interpretation of the spectroscopic data and correct structure elucidation.

When the experimental ^13^C NMR chemical shifts of **1P** and **2P** (Table 1 and SI: II.6) are transferred to the **1R** structure, assuming that the additional methylene described for **1P** and **2P** is actually a quaternary carbon and compared with the data obtained by computational calculation for **1R**, a good fit is obtained. In addition, the rms values are close to those observed for the substance described as **1R** (see SI: III.1, III.2, and III.3). Consequently, **1P** with an ursane skeleton and **2P** with a lupane skeleton are misassigned structures and actually correspond to **1R** with an oleanane skeleton. The structure of **1R** was corroborated because it was also reported as the compound obtained by the photochemical oxidation of oleanolic acid.^19^ As in many other reported examples of structural revisions, **1P** and **2P** with erroneous structures have been published with the correct structure as **1R** several years before.

**Figure 3:**
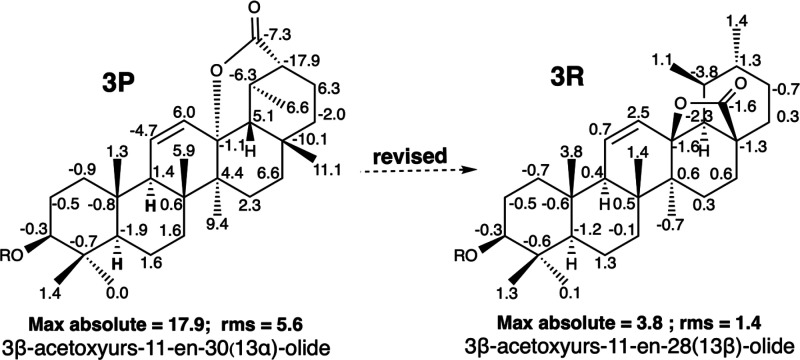
Misassigned structure of 3β-acetoxyurs-11-en-30(13α)-olide (**3P**) and the correct structure of 3β-acetoxyurs-11-en-28(13α)-olide (**3R**). Negative and positive deviations of δ_C_ (calculated value – experimental value) are indicated for each carbon of these structures. Max *y* rms values are underneath each structure. R = CO-CH_3_.

A phytochemical study carried out on *Tripterygium doianum* (Celastraceae) afforded a triterpenoid named 3β-acetoxyurs-11-en-30(13α)-olide (**3P**)^20^ (Figure 3). The structural elucidation of this compound was based on the analysis of 1D and 2D NMR spectroscopic data. Additionally, by computational studies, a conformational search was carried out to establish the conformation of this compound and to rationalize the NOE effects observed. The authors state in the publication that **3P** is the first 30,13α-olide ursene-type triterpenoid isolated from a natural source. This unique structural feature of **3P** prompted us to perform a chemical shift search in NAPROC-13 to determine whether its chemical shift pattern is specific to this compound or if it is found in other structurally related substances. In addition to **3P** acetate, the search on NAPROC-13 provided another triterpene named 3β-acetoxyurs-11-en-28(13β)-olide (**3R**) (Figure 3), isolated from *Pieris japonica* D. DON (Ericaceae).^21^ The ^13^C NMR chemical shifts of **3R** are practically identical to those of **3P** (Table 1 and SI: II.6) with small differences that do not exceed 0.3 ppm in the most unfavorable case. This case is a new example of two triterpenoids with practically identical ^13^C NMR spectra; both triterpenoids have an ursane skeleton and were assigned different structures. This prompted us to perform a computational calculation of the ^13^C NMR spectra of both compounds **3P** and **3R**. The computational results were compared with the experimental data, and a summary of the results obtained is shown in Figure 3. In **3P**, the maximum deviation of 17.9 ppm is that of C-20, which supports the carboxylic group of the lactone; moreover, other significant deviations affect the carbons in the vicinity. In contrast, when the ^13^C NMR data of **3P** are assigned to the **3R** structure, they show a maximum difference of 3.8 and an rms value of 1.4 after several chemical shifts are swapped (SI: III.5). Therefore, the spectroscopic data for both substances show a good fit between the calculated and experimental values for **3R** and do not seem compatible with the **3P** structure (max abs = 17.9, rms = 5.6), as shown in Figure 3. Compound **3R** was isolated from *Euclea natalensis* (Ebenaceae),^29^ and its structure was confirmed by semisynthesis from acetylursolic acid.^30^ Therefore, compound 3β-acetoxyurs-11-en-30(13α)-olide (**3P**) is a misassigned compound, and its structure was revised to 3β-acetoxyurs-11-en-28(13β)-olide (**3R**). This is another example in which a misassigned compound was published correctly more than 25 years ago.

**Figure 4:**
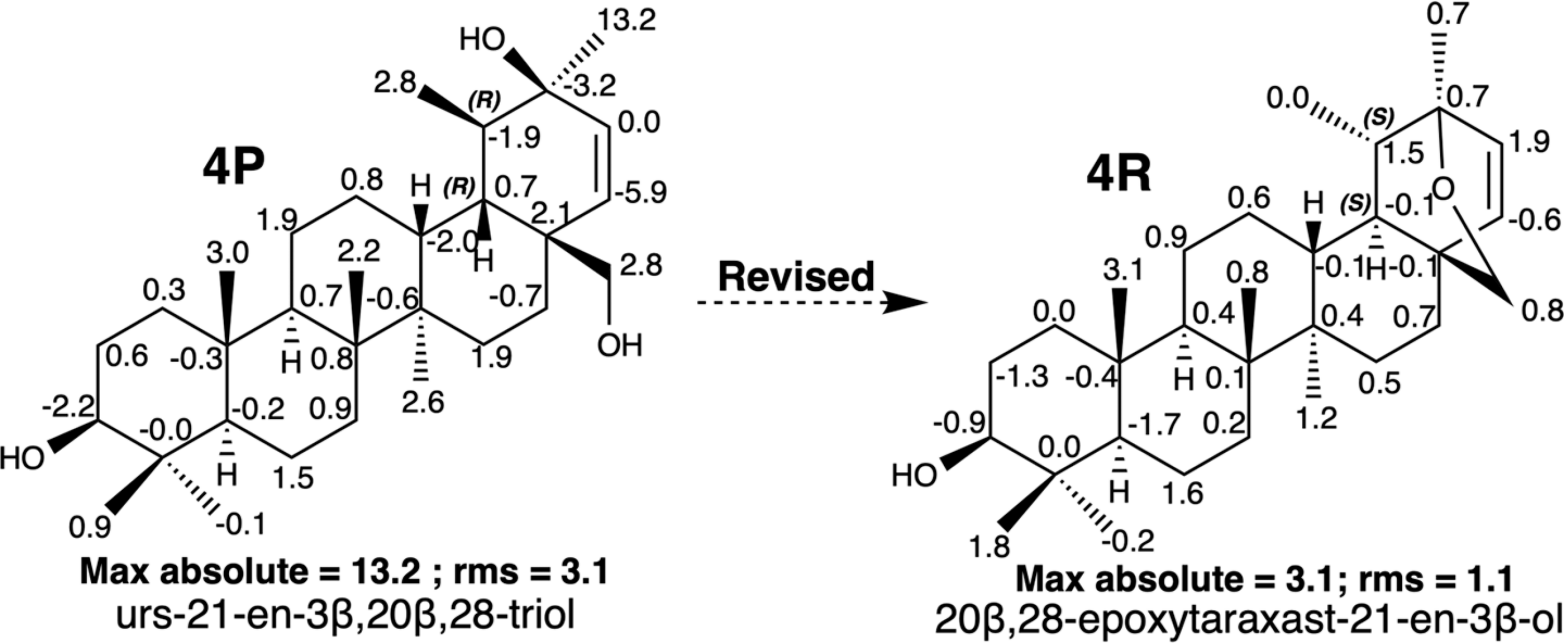
Misassigned structures of urs-21-en-3β,20,28-triol (**4P**) and 20β,28-epoxytaraxast-21-en-3β-ol (**4R**). Negative and positive deviations of δ_C_ (calculated value – experimental value) are indicated for each carbon of the structures. Max *y* rms values are underneath each structure.

The triterpenoid with an ursane skeleton, urs-21-en-3β,20,28-triol (**4P**)^22^ (Figure 4), isolated from *Dracocephalum forrestii* (Labiatae), caught our attention because it has inverted C-18 and C-19 stereocenters with respect to another closely related triterpenoid published in the same article, (3β,18α,19α)-ursane-3,20,28-triol, the structure of which has been recently revised to alloheterobetulin (20,28-epoxytaraxastan-3β-ol).^31^ After being subjected to computational calculations, **4P** generated unmatched ^13^C NMR data mainly in two carbons close to the mentioned stereocenters, methyl C-30 (13.2 ppm) and olefinic CH-22 (−5.9 ppm). These are the largest deviations between the calculated and experimental values, with an rms value of 3.1 (Figure 4; SI: III.6). First, we believed that the correct structure may contain an inversion in the stereocenters C-18 and C-19; hence, we performed computational calculations for the other three stereoisomers. The comparative analysis is provided in the Supporting Information (III.7–III.10). From their analyses, the stereoisomer that best fits the calculated data is 18*S*19*S* (**4P-c**; SI: III.7, III.10) with a max absolute of 6.9 and an rms of 2.5. In this stereoisomer, the hydroxyl group at C-20 is in an axial arrangement, establishing a hydrogen bond with the hydroxymethyl group of C-28. A search for the most downfield chemical shifts of this substance in NAPROC-13^5^ provides another triterpenoid, 20β,28-epoxytaraxaster-21-en-3α-ol (**4R**),^23^ with an 18*S*19*S* configuration and an oxygenated bridge between the C-20 and C-28 positions, instead of both hydroxyl groups in the **4P** compound (Figure 4). This triterpenoid presents ^13^C NMR data (Table 1 and SI: II.6) practically identical to those of **4P**. Comparison of the experimental data of **4R** with those obtained by computational calculation shows a maximum absolute of 3.1 ppm corresponding to methyl C-25 and an rms of 1.1 (Figure 4). Therefore, the structure of urs-21-ene-3β,20β,28-triol (**4P**) was revised to 20β,28-epoxytaraxaster-21-en-3β-ol (**4R**).^23^

**Figure 5:**
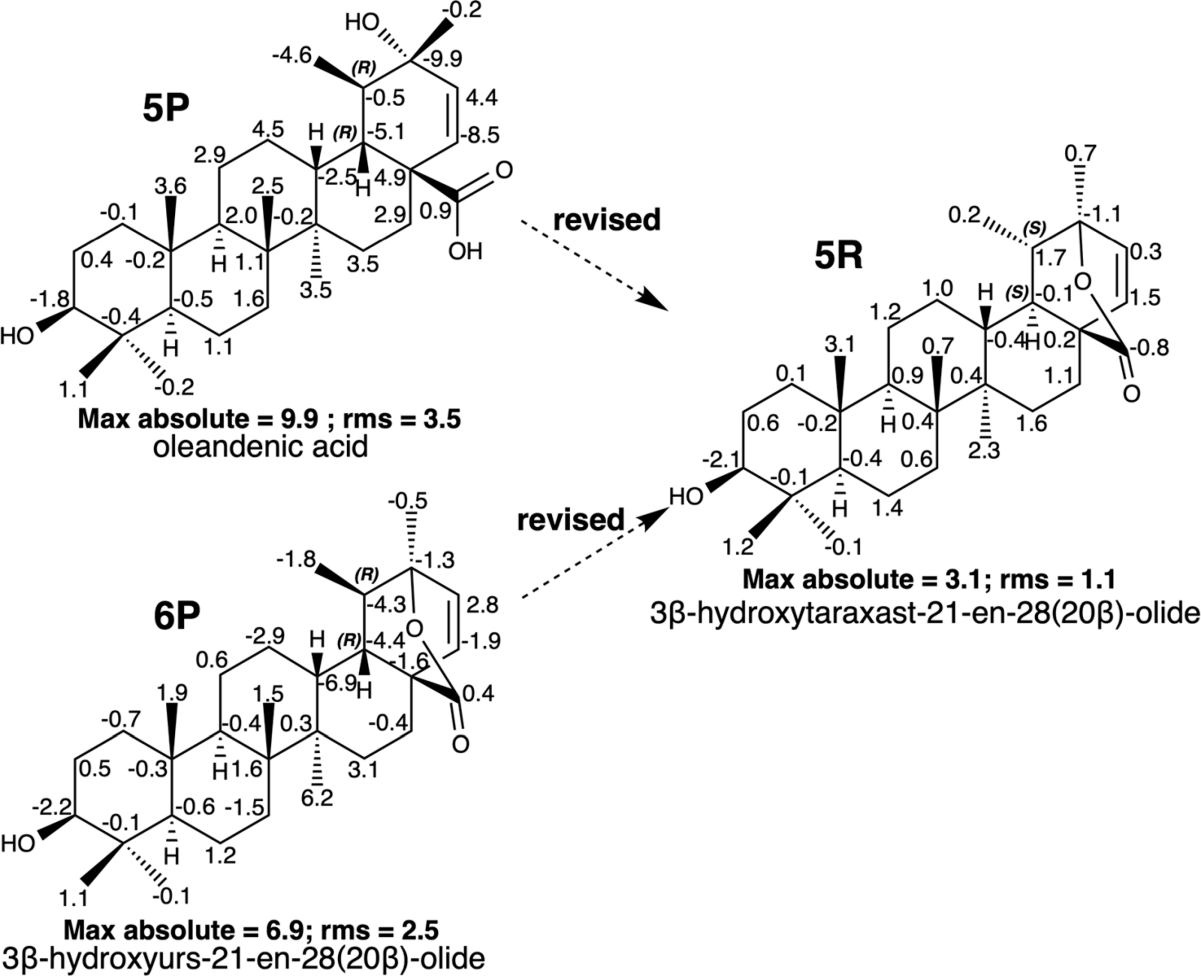
Misassigned structures of oleandenic acid (**5P**) and 3β-hydroxyurs-21-en-28(20β)-olide (**6P**) and the correct structure of 3β-hydroxytaraxast-21-en-28(20β)-olide (**5R**). Negative and positive deviations of δ_C_ (calculated value – experimental value) are indicated for each carbon in the structures. Max and rms values are underneath each structure.

Oleandenic acid (**5P**) (Figure 5) is a triterpenoid with an ursane skeleton isolated from different plant species, such as *Nerium oleander* (Apocynaceae)^24^ or *Ligustrum japonicum* Thunb. (Oleaceae),^32^ showing moderate growth inhibition of HeLa cells.^32^ A search carried out with the ^13^C NMR chemical shifts of **5P**(24,32) (Table 1) in NAPROC-13^5^ yielded, in addition to **5P**, another closely related triterpenoid called 3β-hydroxyurs-21-en-28(20β)-olide (**6P**)^22^ (Figure 5) that was isolated from *Dracocephalum forrestii* (Labiatae).

The free carboxylic and hydroxyl groups present in **5P** are lactonized in **6P** and present an inversion at the C-20 stereocenter. The mass spectra of both compounds showed the same peak at *m*/*z* 455. When analyzing the IR spectrum of hydroxyacid **5P**, we observed the presence of an absorption band at 1740 cm^–1^ compatible with a lactone group. Both substances, **5P** and **6P**, show practically identical ^13^C NMR spectra (Table 1 and SI: II.7), suggesting that they are the same compound. The chemical shift in their ^13^C NMR spectra assigned to C-20 of 83.9 ppm, which is characteristic of carbon that supports the non-carbonyl oxygen of lactone, together with the absorption band at 1740 cm^–1^ in the IR spectrum of **5P** leads to the conclusion that the compound is **6P**. To confirm this hypothesis, the ^13^C NMR spectra of **5P** and **6P** were computationally calculated. As seen in Figure 5, the maximum deviation for **5P** of −9.9 ppm corresponds to C-20, which supports the oxygenated function, and its rms is 3.5; in contrast, the deviation in C-20 is only −1.3 ppm for **6P**. Despite a better approximation between the calculated and experimental data observed (max abs = 6.9 ppm; rms of 2.5), **6P** presents important deviations in the carbons around C-18 and C-19.

In the same publication that describes **6P**,^22^ two other related compounds are described and their structures are also revised: **4P** in the present article and (3β,18α,19α)-ursane-3,20,28-triol, which was previously revised.^31^ Given the revision of these two compounds, we calculated the ^13^C NMR data of the other three stereoisomers of **6P** by inverting the C-18 and C19 carbons, the results of which are summarized in Sections III.14 to III.17 in the Supporting Information.

The analysis of these data indicates that **5R** (Figure 5) is the stereoisomer with a better fit between the calculated and experimental data and presents an inversion in both the C-18 and C-19 stereocenters with respect to the **6P** compound. Consequently, **5P** and **6P** were revised to 3β-hydroxytaraxast-21-en-28(20β)-olide (**5R**), which is a new natural product previously not described in the literature.

**Figure 6:**
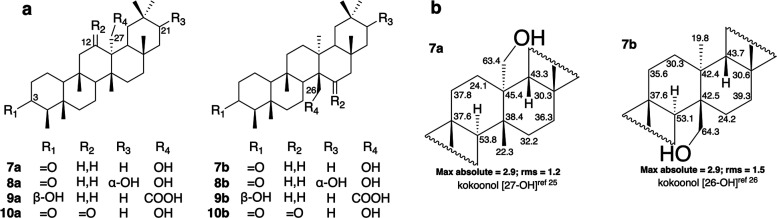
(a) C-26- or C-27-Functionalized friedelanes. (b) Experimental values of the ^13^C NMR chemical shifts of the C and D rings of kokoonol with a hydroxyl group at C-27 (**7a**) or C-26 (**7b**).

A study carried out with *Kokoona zeylanica* Thwaites (Celastraceae)^33^ described the isolation of six new friedelanes, which were characterized as 27-hydroxy D:A-friedooleananes (Figure 6, series a). The ^13^C NMR data of two of these compounds, kokoonol (**7a**) and kokondiol (**8a**), were reported in another paper published by the same research group.^26^ 21α,26-Dihydroxy-D:A-friedoolean-3-one (**8b**) was isolated from *Salacia reticulata* var. Diandra, which also belongs to the family Celastraceae.^34^ Its ^13^C NMR spectroscopic data are very similar to those of **8a**. Subsequently, Giner et al., isolated from *Caloncoba glauca*(25) several new D:A-friedooleananes with oxygenated functions at C-27, including **7a**. In their work, Giner et al. state that the physicochemical properties, such as melting point, optical rotation, and EIMS, of the product they describe as kokoonol (**7a**) were in close agreement with the published data for kokoonol by Gunatilaka et al.^33,26^ Because of the similarity of the spectroscopic data between **8a** and **8b**, Gunatilaka et al.^35^ carried out a thorough study of the NMR data, including 2D experiments and NOE studies on **8a** isolated from *S. reticulata* and concluded that this compound is actually **8b**. As a consequence, they revise the structures of the six D:A-friedooleananes previously described as 27-hydroxy D:A-friedooleananes,^26^ including kokoonol (**7a**) and kokondiol (**8a**), to the corresponding 26-hydroxy D:A-friedooleananes, **7b**, and **8b** (Figure 6, series b). Both the compound isolated from *Kokoona zeylanica*,^33^ as kokoonol, and the compound isolated from *Caloncoba glauca*,^25^ also known as kokoonol, are referenced in Sci-Finder as **7b** with the same CAS number (2183-92-7). Giner et al. did not carry out a comparative study between the ^13^C NMR data of **7a** published by them and the data reported by Gunatilaka et al.^26^ A detailed analysis of the ^13^C NMR data of both compounds, which were described under the same name kokoonol (Table 1), enabled us to observe some differences affecting the quaternary carbons C-13 (42.4/45.4 ppm) and C-14 (42.5/38.4 ppm) bearing C-26 and C-27, in addition to small differences in the neighboring methylenes (Figure 6b). This creates an uncertainty that supports the results obtained both research groups, who describe NOE effects that support the localization of the hydroxyl group at C-26^35^ and at C-27^25^ for kokoonol. Therefore, we decided to computationally calculate the ^13^C NMR spectra of **7a** and **7b** to determine whether the compounds described by both research groups are the same substance and whether the small differences observed resulted from some impurity, an incorrect interpretation of the spectra, or a mistake in the writing of the paper.

**Figure 7:**
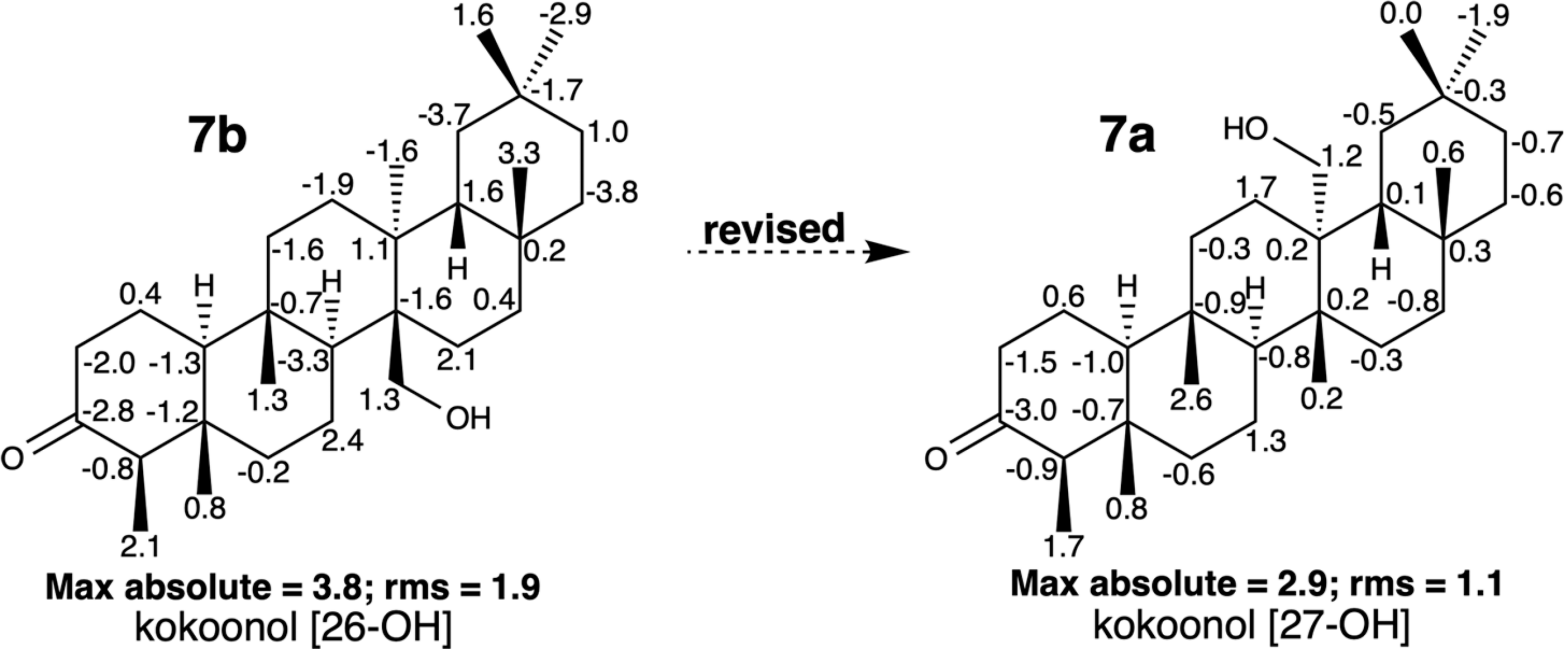
Structures of kokoonol [26-OH] (**7b**) and kokoonol [27-OH] (**7a**). The ^13^C NMR chemical shifts assumed for the two compounds are identical and correspond to those published by Giner et al.^25^ Negative and positive deviations of δ_C_ (calculated value – experimental value) are indicated for each carbon on the structures. Max *y* rms values are underneath each structure.

The result of this computational study (Figure 7; SI: III.18, III.19, and III.20) clearly indicates that the substance described by Gunatilaka et al.^33^ is **7b** and corresponds with the structural revision performed by the same authors in a later publication.^35^ The substance described by Giner et al.^25^ is **7a**, as established by these authors in the original publication; however, the compound is different from the one in Sci-Finder under the same name, kokoonol. The ^13^C NMR data of the compound described by Ginet et al.^25^ show a very good fit with those obtained by computational calculation for **7a** (max abs = 2.9; rms = 1.1) and is superior to those obtained for **7b** (max abs = 3.8; rms = 1.9) (Figure 7; SI: III.18 and III.19). After considering a large number of misassigned signal swaps according to the computational result (SI: III.20; signs marked with an asterisk), the data obtained for the compound described by Gunatilaka et al.^33^ show a closer approximation for **7b** (max abs = 2.9; rms = 1.5) than for **7a** (max abs = 3.9; rms = 1.7). Therefore, these are two different compounds. The substance correctly described by Giner et al.^25^ corresponds to the structure initially erroneously proposed by Gunatilaka et al.^33^ as kokoonol, contrary to the information in Sci-Finder, in which both regioisomers are stored with the same CAS number as 26-hydroxy D:A-friedooleanane. In more recent publications, such as those by Somwong et al.,^36^ citing kokoonol isolated from *Salacia verrucosa*, the location of the hydroxyl group in this molecule remains ambiguous.

To verify the reliability of our method in calculating the ^13^C NMR spectroscopic data for **7a** and **7b**, which present small differences in ^13^C NMR chemical shifts, we carried out a literature search for closely related D:A-friedooleananes to **7a**, yielding trichadenic acid B (**9a**),^37^ a D:A-friedooleanane isolated from *Phyllanthus flexuosus* (Euphorbiaceae). Its structure was initially misassigned as D:A-friedooleanane 26-carboxylic acid (**9b**) (Figure 6a).^38^ Subsequently, its structure was unambiguously revised to D:A-friedooleanane 27-carboxylic acid (**9a**) by X-ray diffraction analysis (Figure 6a).^37^ The results obtained by computational calculation faithfully reproduce the experimental ^13^C NMR data of compound **9a** (SI: III.21).

Two regioisomers, pristimeronol (**10a**)^39^ and salasone A (**10b**)^40^ (Figure 6), are also described in the literature, and the ^13^C NMR spectra of these compounds show great similarities. Additionally, computational calculations of the ^13^C NMR spectra are in agreement with the calculated and experimental ^13^C NMR data for **10a** and **10b**; these computational results perfectly reproduce the small differences observed in their experimental ^13^C NMR data (SI: III.22 and III.23). Consequently, the computational data obtained allow us to correctly assign structures **7a** and **7b** as well as to validate the structures correctly published as **9a**, **10a**, and **10b**.

**Figure 8:**
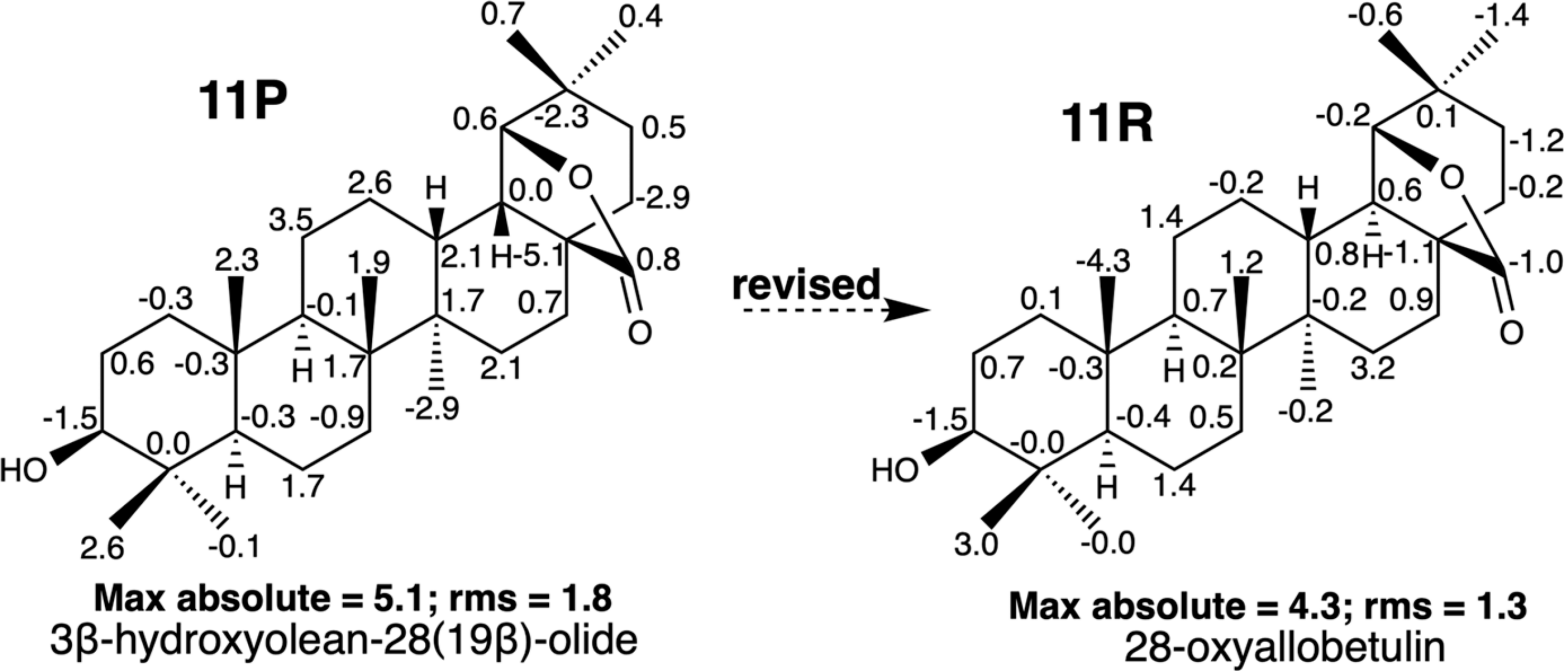
Structures of misassigned 3β-hydroxyolean-28(19β)-olide **11P** and the correct structure of 28-oxyallobetulin **11R**. Negative and positive deviations of δ_C_ (calculated value – experimental value) are indicated for each carbon in the structures. Max *y* rms values are underneath each structure.

In NAPROC-13, we located another pair of triterpenoids, 3β-hydroxyolean-28(19β)-olide (**11P**) and 28-oxyallobetulin (**11R**) (Figure 8), that were assigned the opposite configuration at C-18, although their ^13^C NMR data were almost identical (Table 1 and SI: II.7). Compound **11P** is an oleanane triterpenoid isolated from *Diospyros angustifolia* (Ebinaceae),^27^ whereas **11R** is a germanicane triterpenoid obtained from betulinic acid.^28^ To resolve this inconsistency, computational calculations of both epimers were performed to obtain their theoretical ^13^C NMR chemical shifts and compare them with those published for these compounds. As shown in Figure 8, for **11P**, the maximum deviation between the calculated and its experimental ^13^C NMR chemical shift (max absolute) is 5.1, showing a value of rms = 1.8; for the C-18 epimer **11R**, the values of the experimental and calculated chemical shifts present a closer approximation (max absolute 4.3 and rms 1.3) (Figure 8). The maximum deviation of 5.1 ppm in **11P** corresponds to C-17 bearing the carbonyl carbon of the lactone, while the largest deviation in **11R** corresponds to C-25 methyl, a value that seems to be a misprint considering the value of this chemical shift in other closely related compounds. DP4, with a value of 100%, clearly indicates that the correct structure is **11R**. Unfortunately, ^1^H NMR data, which could be definitive in clarifying the structure of this pair of epimers, including the H_13_–H_18_ coupling constant, are not reported in these articles. However, the structure of **11R** is well established by the published X-ray diffraction data for the corresponding acetate,^41^ which is identical to the one obtained by acetylating **11R**.^28^ Accordingly, 3β-hydroxyolean-28(19β)-olide (**11P**) was revised to 28-oxyallobetulin (**11R**).

**Figure 9:**
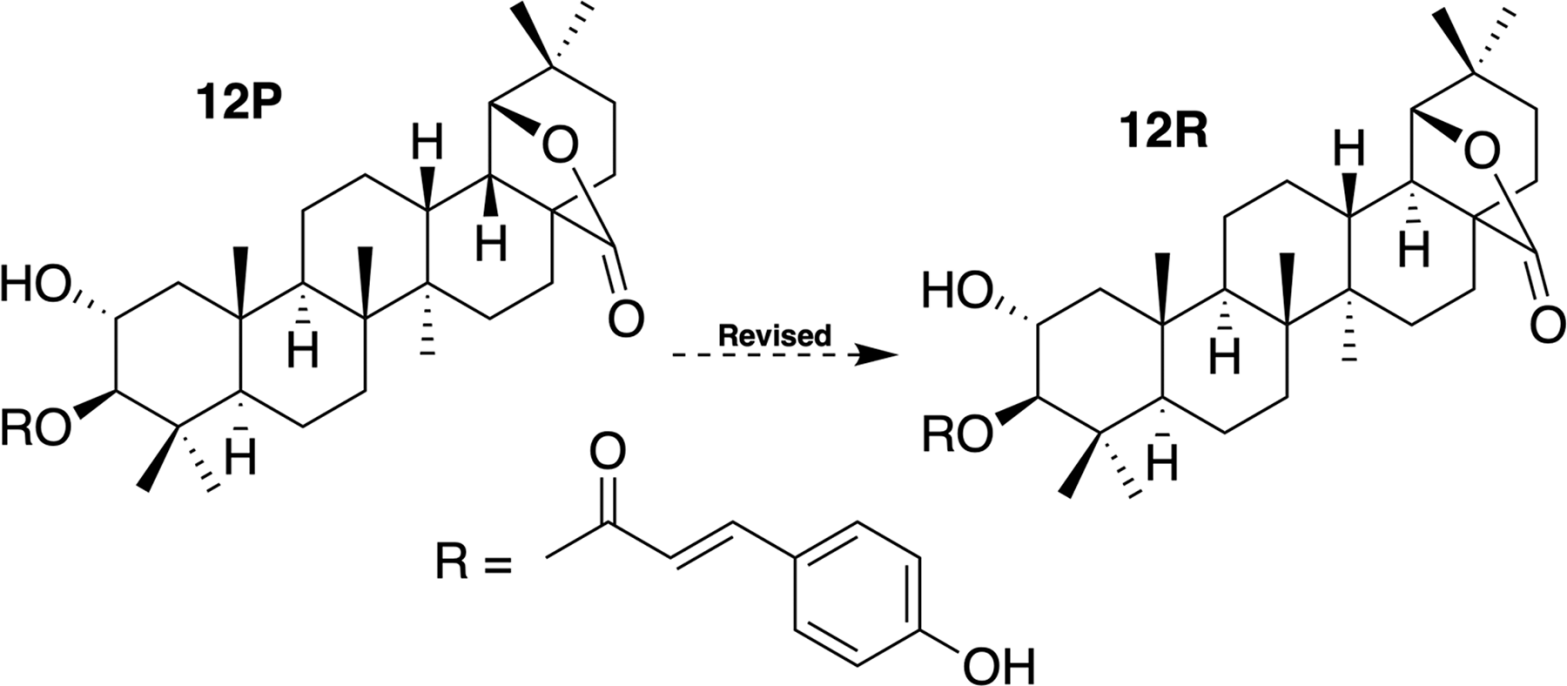
Structure of misassigned compound **12P** and that of revised **12R**. This review was performed by considering the spectroscopic data from **11P**.

In the same paper that described **11P**, another triterpenoid, diospyrosooleanolide (**12P**), a 2α-hydroxy-3β-trans-*p*-coumaroyl derivative of **11P**, was also described. The spectroscopic data of the terpenoid part are very similar to those of compound **11P**; hence, the structure of diospyrosooleanolide (**12P**) was revised to the corresponding C-19 epimer **12R** (Figure 9).

**Figure 10:**
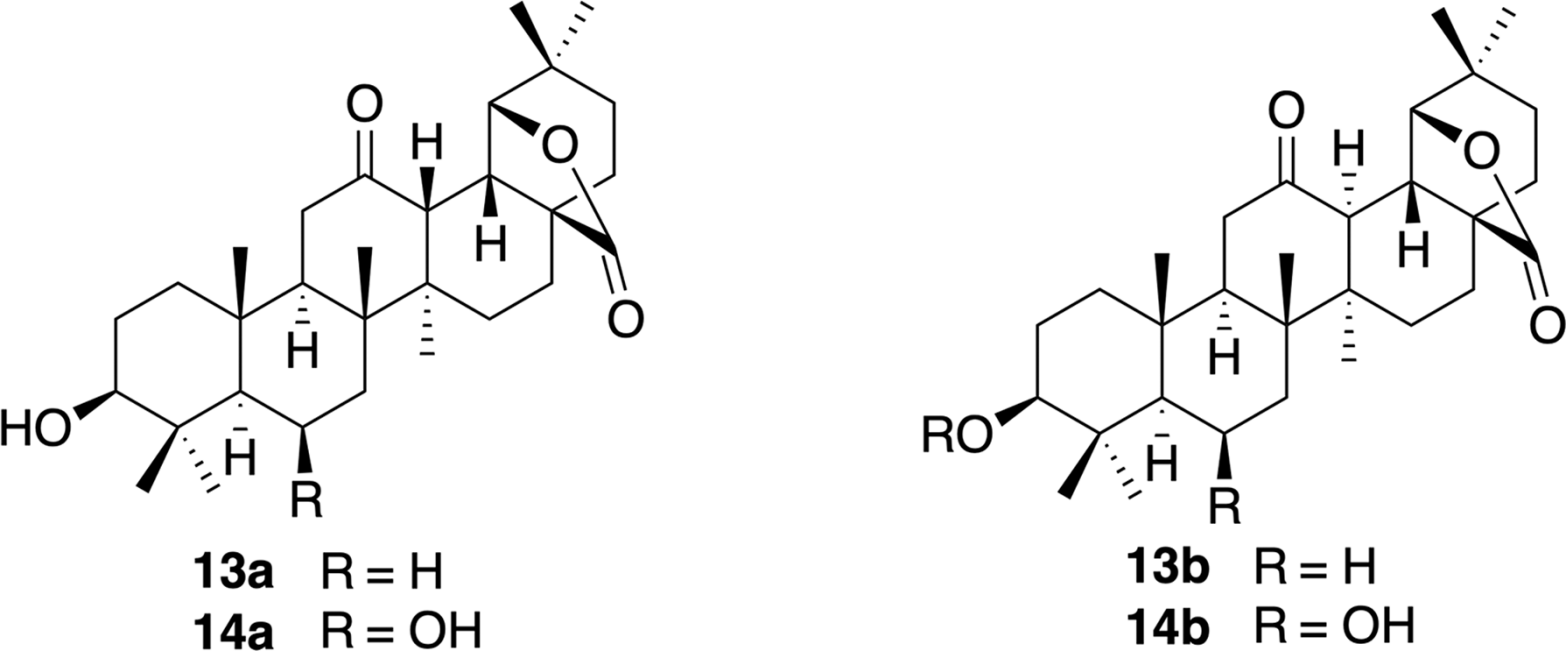
Structures of **13b** and **14b** verified by ^13^C NMR spectroscopic DFT calculation.

To verify whether other compounds structurally related to **11P** with the same configuration at C-18 occur in the literature, a Sci-Finder search was carried out. As a result, we found two other oleanane triterpenoids, **13a**(42) and **14a**(43) (Figure 10), which have, according to this database, the same configuration at C-13 and C-18 as the previously mentioned compounds **11P** and **12P**. In the articles that describe these compounds, the given structures are not coincident with those found in Sci-Finder, which were 3β-hydroxy-12-oxo-13Hα-olean-28,19β-olide (**13b**)^42^ and 3β,6β-dihydroxy-12-oxo-13Hα-olean-28,19β-olide (**14b**)^43^ (Figure 10). These two compounds present an inverse *trans*-diaxial arrangement for H-13/18 in relation to those of **11R** and **12R**. Due to the discrepancy between these publications and the Sci-Finder database and because the C-13 configuration is inverted in these substances compared to the oleananes, we decided to perform ^13^C NMR spectra computational calculations on **13a**/**b** and **14a**/**b** to confirm their structures. Based on the results (SI: III.26–III.29), the above-mentioned publications described the correct representation, and, consequently, the structures reported in Sci-Finder are erroneous. The max absolute and rms terms for **13b** (1.6 and 0.8, respectively) (SI: III.27) versus those obtained for **13a** (2.0 and 5.8, respectively; SI: III.26) clearly indicate that **13b** is the correct structure, which was confirmed by X-ray diffraction.^42^ For the calculation of the maximum absolute and rms values of **13a** and 1**3b**, the chemical shift of δ 39.4 assigned to C-24 was excluded, as it must be a misprint. This value deviated by more than 20 ppm from the value obtained by calculation and the value shown for other compounds with a similar environment published in the same article as **13b**. Similar results were obtained when comparing the statistical results obtained for **14b** versus **14a** since **14b** showed a max ab of 1.7 and an rms term of 0.9 versus the same values for **14a** (max abs = 5.6, rms = 2.0) after swapping several chemical shifts, which significantly improved the adjustment (see SI: III.28 and III.29).

## Conclusions

Searching chemical shifts for suspected misassigned compounds in NAPROC-13 can provide structurally different compounds that share the same ^13^C NMR spectroscopic data. Subsequent calculation of the ^13^C NMR spectra indicated the compound that most closely matched the experimental data in each case. It is relatively common that nowadays misassigned structures were correctly published in the past. In addition, if one of the structures found has been verified by X-ray diffraction, remaining misassigned structures can be checked very quickly. The examples herein provided reinforce the value of the method applied to correct natural product structures.

## Experimental Section

Computational calculations were performed with Spartan’20 (Wavefunction Inc., Irvine, CA, USA). To obtain the ^13^C NMR data by computational calculation, the automated protocol implemented in Spartan’20 was followed.^12^ This protocol consists of five steps: (1) Systematic conformational search using MMFF molecular mechanics, eliminating duplicate conformers and those with energy 40 kJ/mol above the global minimum; (2) geometric calculation using HF/3-21G, also eliminating duplicate conformers and those with energy higher than 40 kJ/mol above the global minimum; (3) energy calculation with the ωB97X-D/6-31G* model and removal of conformers above 15 kJ/mol with respect to the global minimum; (4) geometric calculation with the ωB97X-D/6-31G* model and removal of conformers with energies higher than 10 kJ/mol from that of the global minimum; (5) energy calculation with the ωB97X-V/6-311+G(2df,2p)[6-311G*]; and finally (6) the NMR calculations (following calculation of Boltzmann weights for conformationally flexible molecules) using the ωB97X-D/6-31G* method that has been corrected empirically based on the comparison of calculated and experimental ^13^C shifts for ∼2000 rigid molecules. These corrections are on the order of 1–3 ppm.

## Supporting Information Available

Chemical shift search in NAPROC-13. Cartesian coordinates for optimized structures, computed chemical shifts, and deviations between calculated and experimental values (max absolute and rms terms) (PDF)
