# Supplementary material for: Synergistic Combination of NAPROC-13 and NMR 13C DFT Calculations: A Powerful Approach for Revising the Structure of Natural Products
Source: J Nat Prod. 2023 Sep 7;86(10):2294–303. doi: 10.1021/acs.jnatprod.3c00437 (PMC10616860; doi:10.1021/acs.jnatprod.3c00437)
Supplement: Supplementary file 1 — np3c00437_si_001.pdf [file np3c00437_si_001.pdf]

Supporting Information

Synergistic Combination of NAPROC-13 and NMR ^{13}C DFT Calculations: A Powerful Approach for Revising the Structure of Natural Products.

Hugo A Sánchez-Martínez,[†] Juan A. Morán-Pinzón,[†] Esther del Olmo Fernández,[‡] David López Eguiluz,[§] José F. Adserias Vistué,[§] José L. López-Pérez[*,‡] and Estela Guerrero de León[†,*]

[†] CIPFAR. Departamenteo de Farmacología. Facultad de Medicina. Universidad de Panamá. Ave. Octavio Mendez Pereira, Panama City, Panama

[‡] Departamento de Ciencias Farmacéuticas, Área de Química Farmacéutica, Facultad de Farmacia, CIETUS, IBSAL. Campus Miguel de Unamuno, University of Salamanca, 37007-Salamanca, Spain.

[§] Departamento de Sistemas, Fundación General, University of Salamanca, Fonseca 2, 37002-Salamanca, Spain.

CONTENTS

Supporting Information 1

Synergistic Combination of NAPROC-13 and NMR ^{13}C DFT Calculation: A Powerful Approach for Natural Products Structural Revision...1

I. Revised structure of (Z)-5-amino-5-(1,1,2-trihydroxybuta-1,3-dienyloxy)pentane-6,7,8,9-tetraol (A1) by chemical shift search in NAPROC-13.......................................5

I.1. Search by the NMR ^{13}C chemical shifts of compound A1.................................5

The search is carried out with all ^{13}C NMR chemical shifts of A1 without specifying the multiplicity of each signal and with a tolerance of ±2 ppm........................................5

I.2. Search results using the ^{13}C NMR chemical shifts of A1..................................5

II. Revised structure of (5S,6R,7S,8R)-5-amino-(2Z,4Z)-1,2,3-trihydroxybuta-2,4-dienyloxy-pentane-6,7,8,9-tetraol (B1)...6

II.1. Search by the NMR ^{13}C chemical shifts of compound B1 specifying multiplicity. 6

II.2. Search results using the ^{13}C NMR chemical shifts B1 specifying multiplicity.....7

II.3. Molecular weight search for a range 267 - 268...7

II.4. Boolean search Weight (AND) the NMR ^{13}C chemical shifts of compound B1...8

II.5. Search results using boolean search Weight (AND) the NMR ^{13}C chemical shifts of compound B1..8

II.6. Structure of Adenosine (B2) solved by X-Ray diffraction.................................9

II.7. ^{13}C NMR data for compounds 1- 4. The chemical shifts are presented in order from lowest to highest value.*..10

II.8. ^{13}C NMR data for compounds revised 5, 6, 7a, 7b and 11. The chemical shifts are presented in order from lowest to highest value.*..11

III. Cartesian coordinates and ^{13}C NMR data obtained by computational calculation..12

III.1. Cartesian coordinates of the global minimum conformer of 3β,19α-Dihydroxyursan-28-oic acid (1P)...12

III.2. Cartesian coordinates of the global minimum conformer of licanolide (2P)...14

III.3. Cartesian coordinates of the global minimum conformer of 11α,12α-epoxy-3β-hydroxyolean-28(13β)-olide (1R)..17

III.4. Cartesian coordinates of the global minimum conformer of 3β-acetoxyurs-11-en-30(13α)-olide (3P) 19

III.5. Cartesian coordinates of the global minimum conformer of 3β-acetoxyurs-11-en-28(13β)-olide (3R) 21

III.6. Cartesian coordinates of the global minimum conformer of urs-21-en-3β,20,28-triol (4P) 24

III.7. The four stereoisomers of ursane 4P and the revised structure 4R................26

III.8. Cartesian coordinates of the global minimum conformer of 4P-a.................27

III.9. Cartesian coordinates of the global minimum conformer of 4P-b.................29

III.10. Cartesian coordinates of the global minimum conformer of 4P-c..............31

III.11. Cartesian coordinates of the global minimum conformer of 20β,28-epoxytaraxast-21-en-3β-ol (4R)..34

III.12. Cartesian coordinates of the global minimum conformer of oleandenic acid (5P) 36

III.13. Cartesian coordinates of the global minimum conformer of 3β-hydroxyurs-21-en-28(20β)-olide (6P) 38

III.14. The four stereoisomers of ursane 6P and the revised structure 5R............41

III.15. Cartesian coordinates of the global minimum conformer of 6P-a 41

III.16. Cartesian coordinates of the global minimum conformer of 6P-b 43

III.17. Cartesian coordinates of the global minimum conformer of 3β-hydroxytaraxast-21-en-28(20β)-olide (5R)...46

III.18. Cartesian coordinates of the global minimum conformer and chemical shifts published by Giner et al.{Giner, 1993 #15} for kokoonol [27-OH] (7a)......................48

III.19. Cartesian coordinates of the global minimum conformer and chemical shifts published by Giner et al.{Giner, 1993 #15} for kokoonol[26-OH] (7b).......................50

III.20. Cartesian coordinates of the global minimum conformer and chemical shifts published by Gunatilaka et al.{Gunatilaka, 1982 #23} for kokoonol [26-OH] (7b).....53

III.21. Cartesian coordinates of the global minimum conformer of Trichadenic acid B (9a). 55

III.22. Cartesian coordinates of the global minimum conformer of Pristimeronol (10a). 57

III.23. Cartesian coordinates of the global minimum conformer of salasone A (10b). {Morikawa, 2003 #36}...60

III.24. Cartesian coordinates of the global minimum conformer of 3β-hydroxyolean-28(19β)-olide (11P) 62

III.25. Cartesian coordinates of the global minimum conformer of 28-oxyallobetulin (11R)..64

III.26. Cartesian coordinates of the global minimum conformer of 1β,30-Dihydroxy-3-oxo-D:A-friedooleanane (13a)..67

III.27. Cartesian coordinates of the global minimum conformer of 3β-hydroxy-12-oxo-13Hα-olean-28(19β)-olide (13b) 69

III.28. Cartesian coordinates of the global minimum conformer of 3β,6β-dihydroxy-12-oxo-13Hβ-olean-28(19β)-olide (14a)..71

III.29. Cartesian coordinates of the global minimum conformer of 3β,6β-dihydroxy-12-oxo-13Hα-olean-28(19β)-olide (14b)..74

I. Revised structure of (*Z*)-5-amino-5-(1,1,2-trihydroxybuta-1,3-dienyloxy)pentane-6,7,8,9-tetraol (A1) by chemical shift search in NAPROC-13

I.1. Search by the NMR ^{13}C chemical shifts of compound **A1**

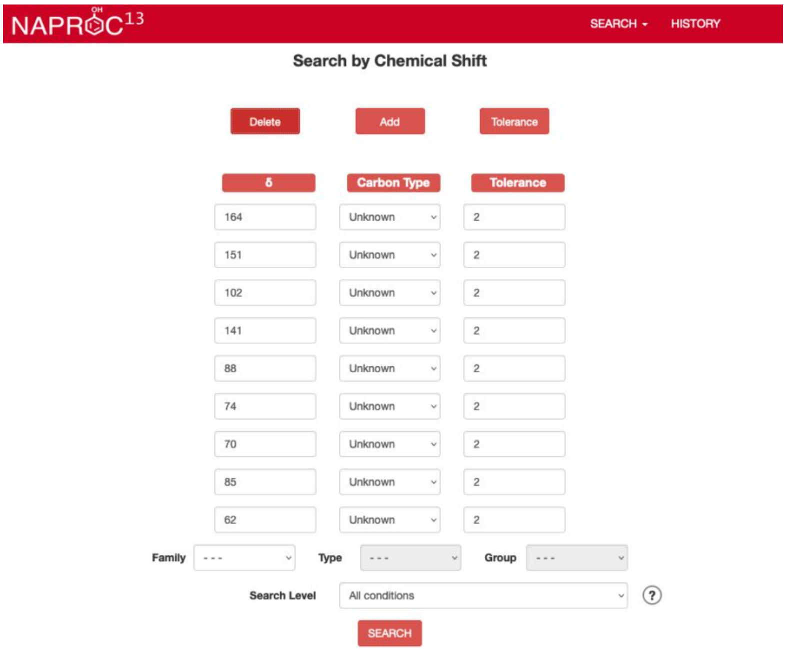

The search is carried out with all ^{13}C NMR chemical shifts of **A1** without specifying the multiplicity of each signal and with a tolerance of ±2 ppm.

I.2. Search results using the ^{13}C NMR chemical shifts of **A1**

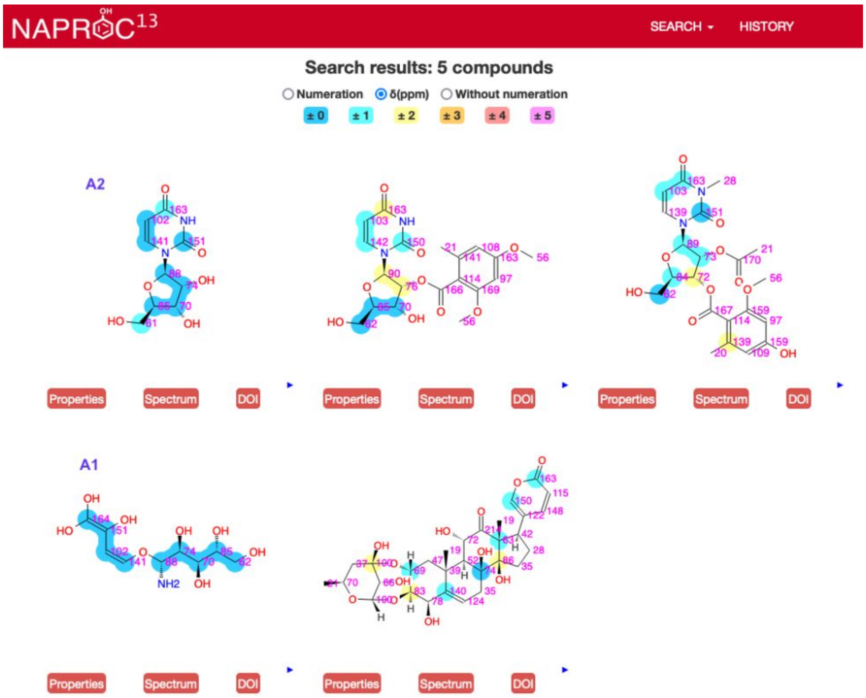

As a result of the ^{13}C NMR chemical shift search of compound **A1**, in addition to compound **A1** itself, uridine (**A2**), two other compounds possessing a uridine moiety as well as a withanolide can be observed. If the search had been carried out specifying the multiplicity of the signals, the latter

compound would not be present since its signals at 150, 140, 86, 74, and 63 ppm do not match the multiplicities of the corresponding signals present in the ^{13}C NMR spectrum of **A1**.

Siebatcheu et al.{Siebatcheu, 2023 #54} provide an HR-ESIMS m/z 268.1041 [M + H]$^+$ value for **A1**. Probably this molecular ion actually corresponds to HR-ESIMS m/z 268.1041 [M + Na]$^+$, matching that of **A2**. In addition, in its mass spectrum (see supplementary material in the original publication){Siebatcheu, 2023 #54} another peak is observed at 245.0769 which could also correspond to A2.

II. Revised structure of (5*S*,6*R*,7*S*,8*R*)-5-amino-(2*Z*,4*Z*)-1,2,3-trihydroxybuta-2,4-dienyloxy-pentane-6,7,8,9-tetraol (B1)

II.1. Search by the NMR ^{13}C chemical shifts of compound **B1** specifying multiplicity.

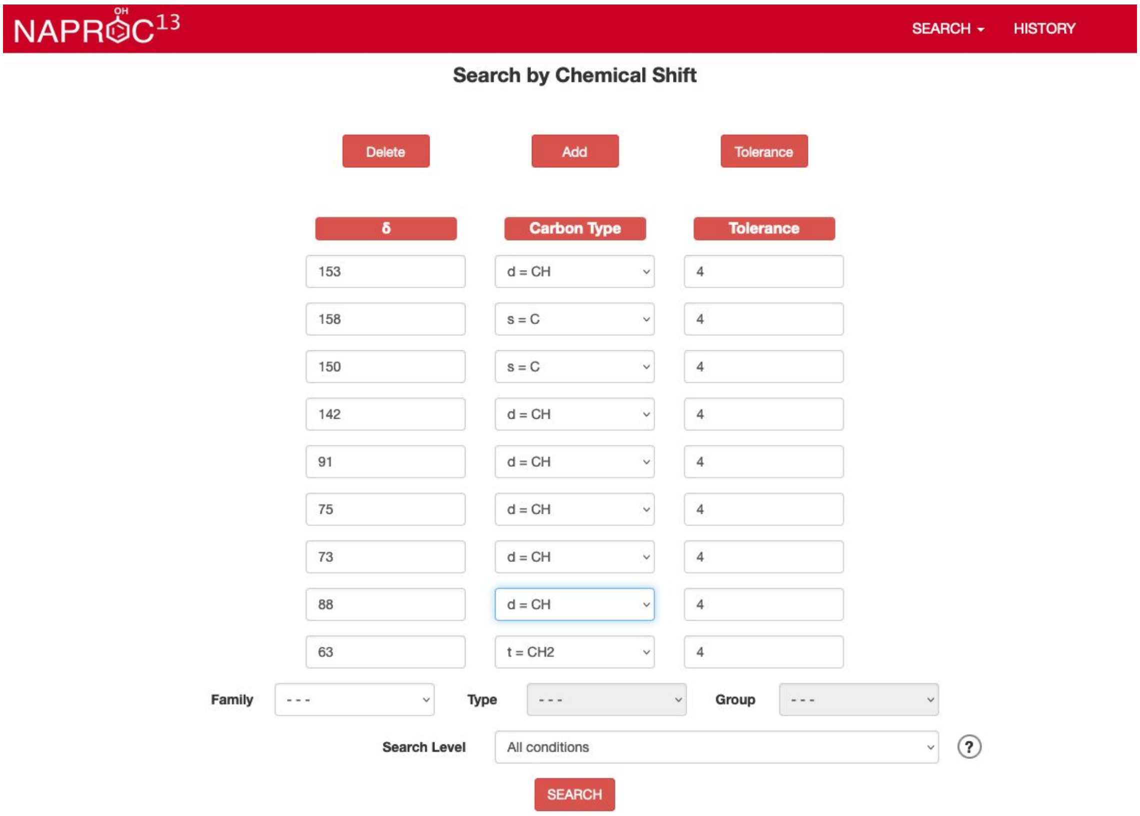

For the **B1** chemical shift search, multiplicity was considered. In this way, we can appreciate the versatility of NAPROC-13.

II.2. Search results using the ^{13}C NMR chemical shifts **B1** specifying multiplicity.

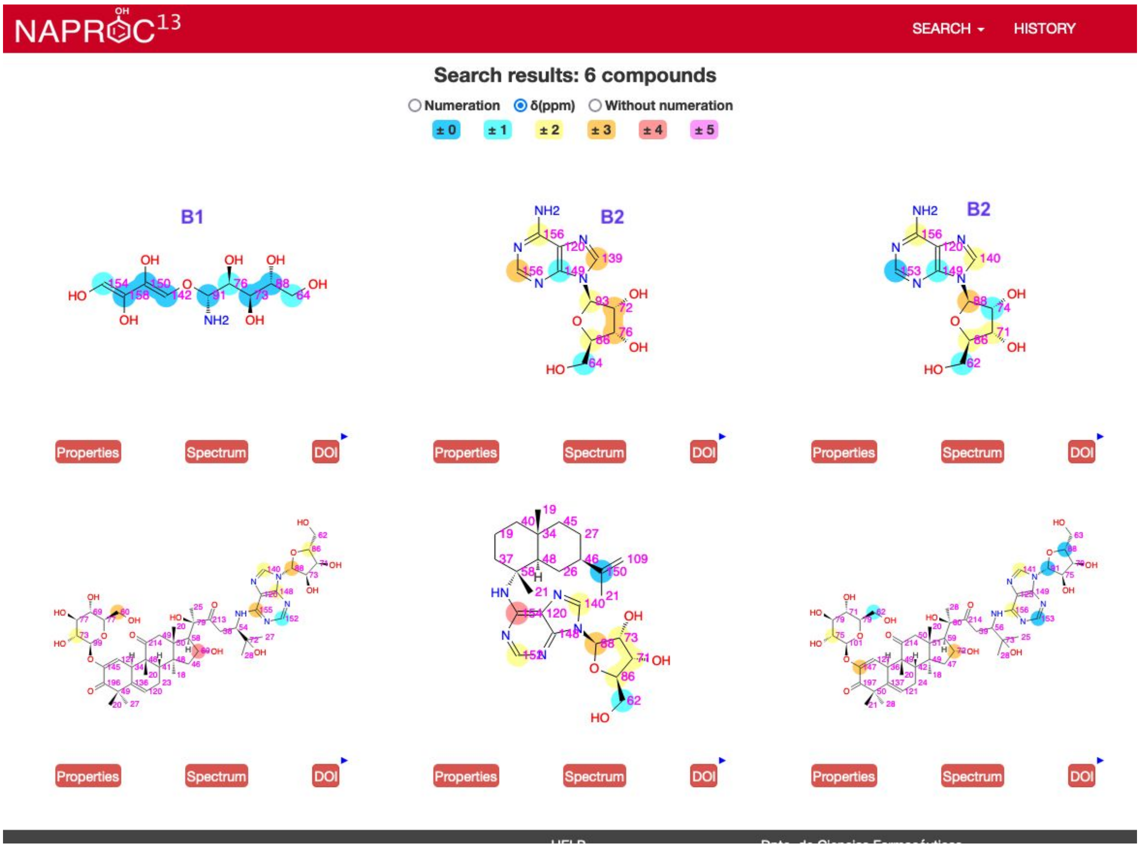

II.3. Molecular weight search for a range 267 - 268

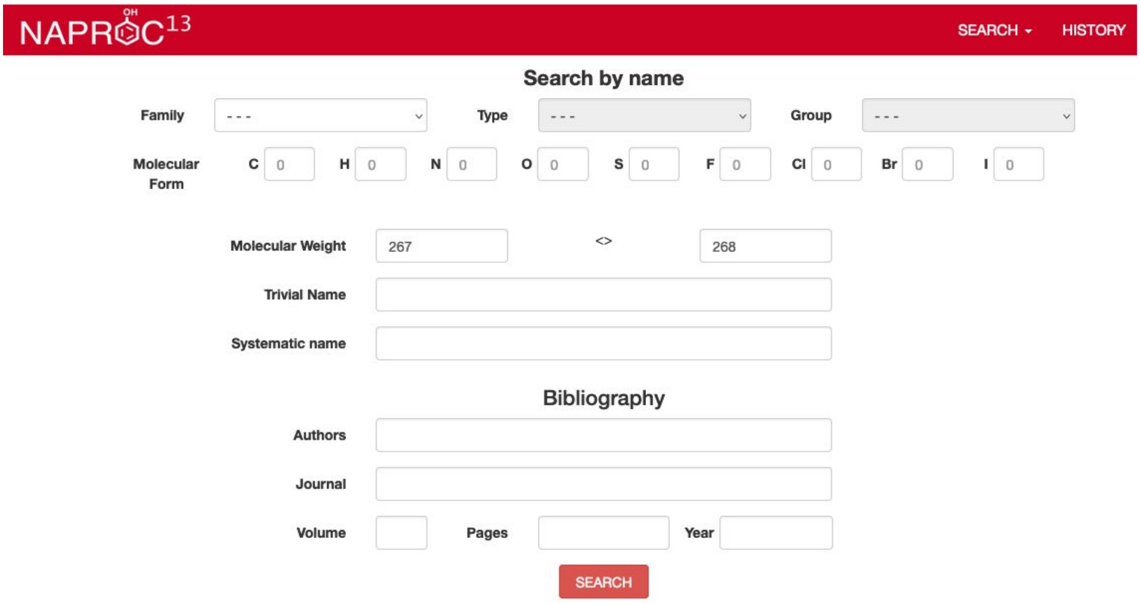

II.4. Boolean search Weight (AND) the NMR ^{13}C chemical shifts of compound **B1**

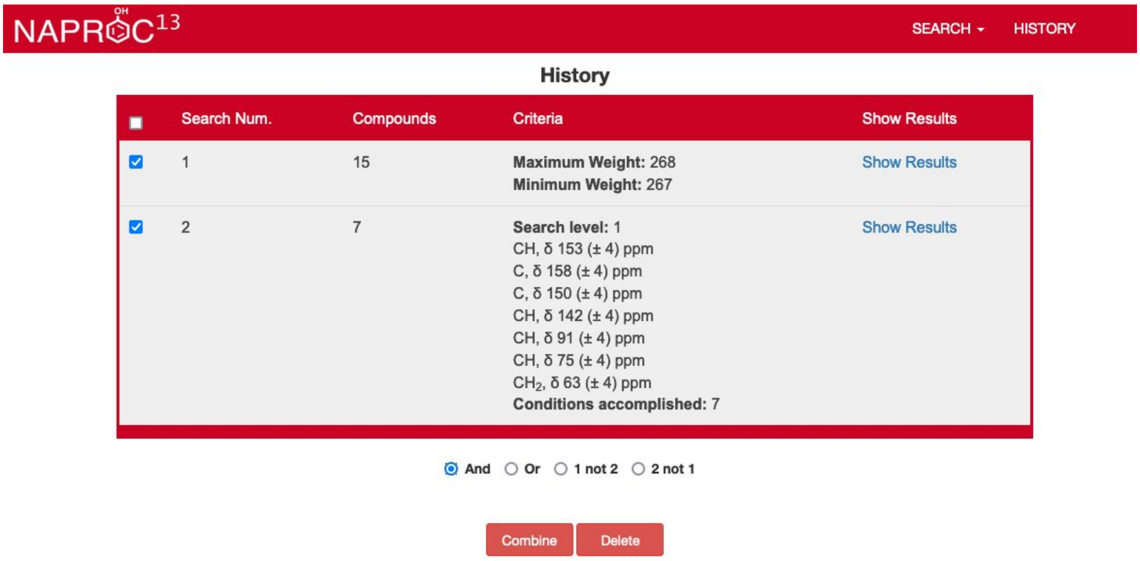
NAPROC13 SEARCH HISTORY

History

	Search Num.	Compounds	Criteria	Show Results
☑	1	15	**Maximum Weight:** 268<br>**Minimum Weight:** 267	Show Results
☑	2	7	**Search level:** 1<br>CH, δ 153 (± 4) ppm<br>C, δ 158 (± 4) ppm<br>C, δ 150 (± 4) ppm<br>CH, δ 142 (± 4) ppm<br>CH, δ 91 (± 4) ppm<br>CH, δ 75 (± 4) ppm<br>CH_2, δ 63 (± 4) ppm<br>**Conditions accomplished:** 7	Show Results

And Or 1 not 2 2 not 1

Combine Delete

II.5. Search results using boolean search Weight (AND) the NMR ^{13}C chemical shifts of compound **B1**

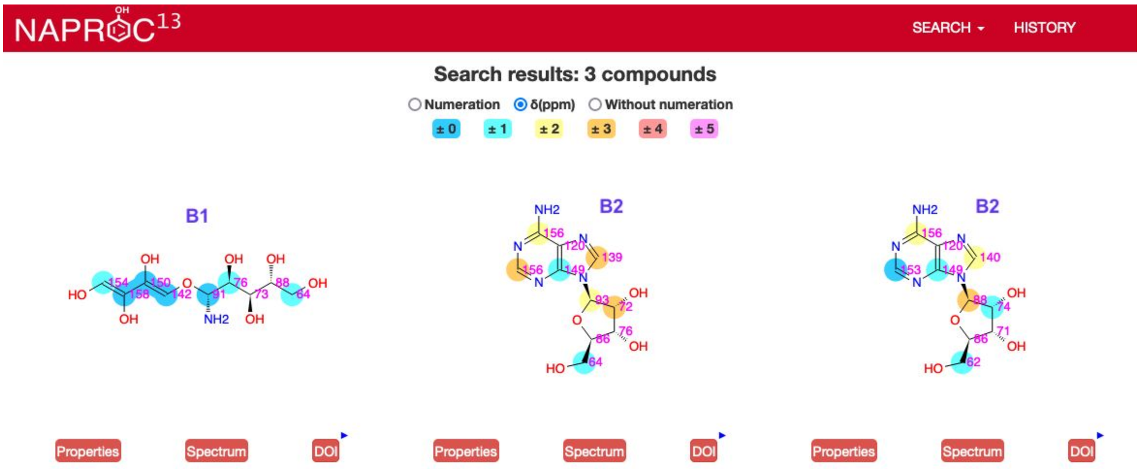

A combined search taking also into account the molecular weight deduced from their mass spectra allows us to narrow down the result to only **B1** and **B2**. All the carbons of both substances are color-coded according to the deviations between the chemical shifts searched for and those of the compound found, except for the C-5 of compound **B2** which resonates at 121 ppm, which is not present in **B1**. The availability of the ^{13}C NMR spectrum in the SI in both publications that describe it allows us to observe the presence of a signal at 121 ppm, which has been neglected by the authors.

II.6. Structure of Adenosine (B2) solved by X-Ray diffraction.

NAPROC[13] SEARCH HISTORY

Properties

Trivial Name: Adenosine

Systematic Name:

Family : Nitrogenous-bases | **Group :** Nucleosides | **Type :** Purines

Solvent : X | **Molecular Formula :** $C_{10}H_{13}N_5O_4$ | **Molecular Weight :** 267.2421

Bibliography : Chang, C.J., Diaz, L.E., Woolfenden, W.R., Grant, D.M. *J Org Chem* (1982) *47*, 5318-21

±0 ±1 ±2 ±3 ±4 ±5

^{13}C NMR Chemical Shifts

Num	Num2	Types	δ(ppm)
2	2	CH	155.6
4	4	C	148.9
5	5	C	120.4
6	6	C	155.6
8	8	CH	138.6
1'	101	CH	92.7
2'	102	CH	72.1
3'	103	CH	75.8
4'	104	CH	85.6
5'	105	CH2	63.6

Structure

Numeration δ(ppm) Without numeration

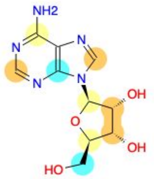

Comments: Recorded in solid-state. Structure solved by X-ray diffraction: Caminiti, R., Ortaggi, G., Mazzei, R.A., Ballirano, P., Rizzi, R.; Powder Diffraction (2000) 15: 112-5. 10.1017/S0885715600010940

Back

II.6 ^{13}C NMR data for compounds 1- 4.* The chemical shifts are ordered from higher to lower values.

C	1P[13]		2P[14]		1R[15]		1R		3P[20]		3R[21]		3R		4P[22]		4R[23]		4R
solv.	C	num.	C	num.	P	num.	calculated	num.	C	num.	C	num.	calculated	num.	C	num.	C	num.	calculated
28	179.4, C	28	179.3, C	28	178.9, C	28	178.4, C	30	179.9, C	28	179.7, C	28	178.3, C	22	140.6, CH	22	140.6, CH	22	140.0, CH
19	87.5, C	20	87.5, C	13	87.6, C	13	86.7, C	11	133.4, CH	11	133.2, CH	11	134.1, CH	21	133.0, CH	21	133.0, CH	21	134.9, CH
3	78.8, CH	3	75.8, CH	3	77.9, CH	3	77.4, CH	12	129.0, CH	12	129.0, CH	12	131.5, CH	3	79.4, CH	3	79.0, CH	3	78.1, CH
18	57.1, CH	19	56.9, CH	12	57.3, CH	12	58.0, CH	13	89.7, C	13	89.6, C	13	88.1, C	20	74.2, C	20	74.2, C	20	74.9, C
5	54.5, CH	9	52.6, CH	5	55.0, CH	5	53.9, CH	3	80.7, CH	3	80.6, CH	3	80.4, CH	28	65.9, CH_2	28	65.9, CH_2	28	66.7, CH_2
9	52.7, CH	18	50.2, CH	11	52.8, CH	11	53.6, CH	20	60.7, CH	18	60.6, CH	18	58.4, CH	5	55.4, CH	5	55.4, CH	5	53.7, CH
13	50.6, CH	5	49.4, CH	18	51.2, CH	9	51.4, CH	5	55.0, CH	5	54.9, CH	5	53.8, CH	9	50.6, CH	9	50.6, CH	9	51.0, CH
20	49.6, CH	13	48.0, CH	9	49.8, CH	18	48.7, CH	9	53.0, CH	9	53.0, CH	9	53.4, CH	18	45.4, CH	18	45.4, CH	19	45.7, CH
17	43.8, C	17	43.7, C	17	44.1, C	17	43.1, C	17	45.2, C	17	45.1, C	17	43.9, C	19	44.2, CH	19	44.2, CH	18	45.3, CH
8	41.2, C	14	41.3, C	14	41.7, C	8	41.6, C	14	42.0, C	14	41.9, C	14	42.6, C	14	41.6, C	14	41.6, C	14	42.0, C
14	40.5, C	8	40.4, C	8	40.9, C	14	41.2, C	8	41.8, C	8	41.7, C	8	42.3, C	8	40.6, C	8	40.7, C	8	40.8, C
4	38.8, C	1	37.5, CH_2	4	39.4, C	4	38.7, C	18	40.4, CH	20	40.3, CH	20	39.5, CH	1	38.9, CH_2	4	38.9, C	4	38.9, C
1	38.1, CH_2	4	37.2, C	1	38.7, CH_2	1	37.8, CH_2	19	38.2, CH	1	38.1, CH_2	1	37.4, CH_2	13	38.9, CH	1	38.8, CH_2	1	38.8, CH_2
22	37.7, CH_2	10	36.4, C	19	38.0, CH_2	19	36.7, CH_2	1	38.1, CH_2	19	38.0, CH	4	37.3, C	4	38.8, C	13	38.7, CH	13	38.6, CH
10	36.4, C	12	34.1, CH_2	10	36.8, C	10	36.1, C	4	37.9, C	4	37.9, C	19	36.6, CH	17	37.6, C	17	37.6, C	17	37.5, C
7	34.2, CH_2	29	33.0, CH_3	21	34.4, CH_2	21	33.7, CH_2	10	36.4, C	10	36.3, C	10	35.8, C	10	37.2, C	10	37.2, C	10	36.8, C
27	33.2, CH_3	22	32.5, CH_2	29	33.0, CH_3	29	32.8, CH_3	7	31.4, CH_2	22	31.4, CH_2	22	31.6, CH_2	7	34.0, CH_2	7	33.9, CH_2	7	34.1, CH_2
12	31.4, CH_2	16	30.3, CH_2	20	31.5, C	7	31.3, CH_2	22	31.3, CH_2	7	31.2, CH_2	7	31.3, CH_2	23	28.0, CH_3	23	28.0, CH_3	16	28.2, CH_2
21	31.1, CH_2	15	29.5, CH_2	7	31.4, CH_2	20	30.8, C	16	30.9, CH_2	21	30.8, CH_2	21	30.2, CH_2	2	27.5, CH_2	16	27.5, CH_2	12	27.9, CH_2
15	29.7, CH_2	2	27.8, CH_2	23	28.4, CH_3	23	27.8, CH_3	23	27.8, CH_3	23	27.9, CH_3	23	27.9, CH_3	12	27.3, CH_2	2	27.4, CH_2	15	27.9, CH_2
23	27.7, CH_3	23	26.8, CH_3	22	27.7, CH_2	22	27.7, CH_2	15	25.6, CH_2	15	25.6, CH_2	15	25.9, CH_2	16	27.3, CH_2	12	27.3, CH_2	23	27.8, CH_3
2	26.9, CH_2	7	26.4, CH_2	2	27.6, CH_2	2	27.4, CH_2	2	23.4, CH_2	2	23.4, CH_2	16	23.5, CH_2	15	26.5, CH_2	15	26.6, CH_2	2	25.3, CH_2
16	26.7, CH_2	21	24.8, CH_2	15	27.0, CH_2	15	26.9, CH_2	21	22.9, CH_2	16	22.8, CH_2	2	22.9, CH_2	30	22.2, CH_3	30	22.2, CH_3	30	22.9, CH_3
29	23.6, CH_3	25	23.4, CH_3	30	23.4, CH_3	30	23.5, CH_3	25	19.2, CH_3	30	19.1, CH_3	26	20.6, CH_3	29	21.9, CH_3	11	21.3, CH_2	11	22.2, CH_2
11	21.2, CH_2	11	21.6, CH_2	16	21.6, CH_2	16	22.2, CH_2	26	19.0, CH_3	26	18.9, CH_3	25	20.0, CH_3	11	21.3, CH_2	29	20.9, CH_3	29	20.9, CH_3
30	20.0, CH_3	26	21.1, CH_3	27	20.4, CH_3	26	21.3, CH_3	28	18.1, CH_3	6	17.9, CH_2	30	19.5, CH_3	6	18.2, CH_2	6	18.2, CH_2	6	19.8, CH_2
24	18.8, CH_3	6	20.9, CH_2	26	18.9, CH_3	27	20.5, CH_3	29	17.9, CH_3	27	17.8, CH_3	6	19.0, CH_2	25	16.3, CH_3	25	16.3, CH_3	25	19.4, CH_3
6	17.5, CH_2	30	18.9, CH_3	6	18.8, CH_2	25	19.4, CH_3	6	17.7, CH_2	29	17.6, CH_3	29	19.0, CH_3	26	15.7, CH_3	26	15.7, CH_3	24	17.5, CH_3
26	17.2, CH_3	27	17.3, CH_3	25	17.3, CH_3	6	19.0, CH_2	27	16.2, CH_3	24	16.0, CH_3	27	18.3, CH_3	24	15.3, CH_3	24	15.4, CH_3	26	16.2, CH_3

25	15.1, CH_3	24	17.0, CH_3	24	16.0, CH_3	24	16.1, CH_3	24	16.1, CH_3	25	16.0, CH_3	24	17.4, CH_3	27	14.3, CH_3	27	14.3, CH_3	27	15.5, CH_3
								1'	171.1, C	1'	170.9, C	1'	171.2, C						
								2'	21.4, CH_3	2'	21.4, CH_3	2'	20.5, CH_3						

*The chemical shifts correspond to those given in the publications describing each product. After the computational calculation, some chemical shifts have been swapped (To see these swaps for each compound, see: III.1 – III.29). Results of ^{13}C NMR data obtained by DFT in green color

II.7. ^{13}C NMR data for compounds revised 5, 6, 7a, 7b and 11.* The chemical shifts are ordered from higher to lower values.

C	5P[24]		6P[22]		5R		7a[25]		7b[26]		7a		11P[27]		11R[28]		11R
solv.	C	num.	C		calculated	num.	C	num.	C		calculated	num.	C	num.	C	num.	calculated
28	175.8, C	28	175.5, C	28	174.7, C	3	213.3, C	3	213.2, C	3	210.3, C	28	179.8, C	28	179.9, C	28	178.8, C
22	138.7, CH	22	138.4, CH	22	139.9, CH	27	63.4, CH_2	27	64.3, CH_2	27	64.6, CH_2	19	85.9, CH	19	86.0, CH	19	85.8, CH
21	134.0, CH	21	133.7, CH	21	134.0, CH	10	59.7, CH	4	60.1, CH	10	58.7, CH	3	78.8, CH	3	78.9, CH	3	77.3, CH
20	84.0, C	20	83.7, C	20	82.6, C	4	58.4, CH	10	58.2, CH	4	57.5, CH	5	55.4, CH	5	55.5, CH	5	55.0, CH
3	79.2, CH	3	79.5, CH	3	77.4, CH	8	53.8, CH	8	53.1, CH	8	53.0, CH	9	51.2, CH	9	51.2, CH	9	51.9, CH
5	55.6, CH	5	55.4, CH	5	55.0, CH	13	45.4, C	18	43.7, CH	13	45.6, C	18	46.6, CH	18	46.7, CH	18	47.2, CH
9	50.7, CH	9	50.4, CH	9	51.3, CH	18	43.3, CH	2	42.6, CH_2	18	43.4, CH	17	46.0, C	20	46.1, C	17	44.9, C
17	48.4, C	17	48.1, C	17	48.3, C	5	42.3, C	14	42.5, C	5	41.6, C	8	40.7, C	8	40.5, C	8	41.0, C
18	47.5, CH	18	47.3, CH	18	47.2, CH	2	41.6, CH_2	5	42.4, C	6	40.9, CH_2	4	40.5, C	14	39.9, C	14	40.2, C
19	44.8, CH	19	44.6, CH	19	46.3, CH	6	41.5, CH_2	13	42.4, C	2	40.1, CH_2	1	38.9, CH_2	1	38.9, CH_2	1	39.0, CH_2
13	42.5, CH	13	42.2, CH	13	41.8, CH	22	40.1, CH_2	6	41.7, CH_2	22	39.5, CH_2	14	38.8, C	4	38.9, C	4	38.8, C

14	41.5, C	14	41.2, C	14	41.6, C	14	38.4, C	22	39.3, CH_2	14	38.6, C	10	37.2, C	10	37.2, C	10	36.9, C
8	40.8, C	8	40.6, C	8	41.0, C	11	37.8, CH_2	9	37.6, C	11	37.5, CH_2	13	35.9, CH	13	36.0, CH	13	36.7, CH
1	39.1, CH_2	1	38.9, CH_2	1	39.0, CH_2	9	37.6, C	16	36.2, CH_2	9	36.7, C	7	33.7, CH_2	7	33.7, CH_2	7	34.2, CH_2
4	39.1, C	4	38.8, C	4	38.7, C	19	37.1, CH_2	11	35.6, CH_2	19	36.6, CH_2	20	33.5, C	17	33.5, C	20	33.6, C
10	37.4, C	10	37.1, C	10	36.9, C	16	36.3, CH_2	19	35.6, CH_2	16	35.5, CH_2	22	32.2, CH_2	21	32.3, CH_2	22	31.7, CH_2
7	34.2, CH_2	7	34.0, CH_2	7	34.6, CH_2	29	35.8, CH_3	29	34.7, CH_3	29	33.9, CH_3	21	31.9, CH_2	16	31.9, CH_2	21	31.1, CH_2
23	28.2, CH_3	23	28.0, CH_3	15	28.4, CH_2	28	32.8, CH_3	21	33.1, CH_2	28	32.2, CH_3	29	28.7, CH_3	30	28.7, CH_3	15	29.6, CH_2
2	27.6, CH_2	2	27.4, CH_2	12	28.3, CH_2	21	32.6, CH_2	28	32.1, CH_3	15	31.9, CH_2	30	27.9, CH_3	23	27.9, CH_3	29	28.1, CH_3
12	27.6, CH_2	12	27.3, CH_2	2	28.0, CH_2	15	32.2, CH_2	30	31.9, CH_3	21	31.9, CH_2	15	27.8, CH_2	15	27.9, CH_2	2	28.0, CH_2
15	27.1, CH_2	15	26.8, CH_2	23	27.9, CH_3	30	30.6, CH_3	17	30.6, C	17	30.6, C	2	27.3, CH_2	2	27.3, CH	23	27.9, CH_3
16	25.7, CH_2	16	25.4, CH_2	16	26.5, CH_2	17	30.3, C	15	30.3, CH_2	30	30.6, CH_3	16	26.4, CH_2	11	26.5, CH_2	12	27.7, CH_2
11	21.3, CH_2	11	21.1, CH_2	11	22.3, CH_2	20	28.5, C	20	28.5, C	20	28.2, C	12	25.5, CH_2	22	25.5, CH_2	16	26.4, CH_2
30	21.3, CH_3	30	21.0, CH_3	30	21.7, CH_3	12	24.1, CH_2	12	24.2, CH_2	12	25.8, CH_2	25	25.4, CH_3	29	23.9, CH_3	30	24.0, CH_3
29	19.9, CH_3	29	19.7, CH_3	29	19.9, CH_3	1	22.5, CH_2	1	22.5, CH_2	1	23.1, CH_2	23	23.9, CH_3	12	20.9, CH_2	11	22.2, CH_2
6	18.5, CH_2	6	18.2, CH_2	6	19.6, CH_2	26	22.3, CH_3	7	20.8, CH_2	26	22.5, CH_3	11	20.8, CH_2	6	18.1, CH_2	6	19.6, CH_2
26	16.5, CH_3	25	16.2, CH_3	25	19.3, CH_3	7	18.6, CH_2	25	19.8, CH_3	25	20.8, CH_3	6	18.2, CH_2	25	16.5, CH_3	25	19.6, CH_3
25	15.9, CH_3	26	15.7, CH_3	24	16.5, CH_3	25	18.2, CH_3	26	17.9, CH_3	7	19.9, CH_2	27	16.4, CH_3	26	15.5, CH_3	24	16.6, CH_3
24	15.6, CH_3	24	15.3, CH_3	26	16.4, CH_3	24	14.9, CH_3	24	14.7, CH_3	24	15.7, CH_3	26	15.3, CH_3	24	15.3, CH_3	26	16.4, CH_3
27	14.3, CH_3	27	14.1, CH_3	27	16.4, CH_3	23	7.1, CH_3	23	7.0, CH_3	23	8.8, CH_3	24	13.6, CH_3	27	13.7, CH_3	27	16.2, CH_3

*The chemical shifts correspond to those given in the publications describing each product. After the computational calculation, some chemical shifts have been swapped (To see these swaps for each compound, see: III.1 – III.29). Results of ^{13}C NMR data obtained by DFT in green color

III. Cartesian coordinates and ^{13}C NMR data obtained by computational calculation

In this section, the 2D structure of each of the triterpenoids under study is depicted. The 2D representation on the left shows the differences in chemical shifts for each carbon between the calculated and experimental values with positive or negative values depending on whether the calculated value is higher or lower than the experimental values (δ_{cal}-δ_{exp}). The 2D representation on the right shows the numbering recommended by IUPAC for triterpenoids, which is used in this publication. The column "no" shows the numbering according to the mol2 coordinates. "IUPAC no" indicates the numbering recommended by IUPAC. An asterisk to the right of the table indicates that the experimental chemical shift value has been swapped according to the calculated value. ^{13}C MNR chemical shifts calculated values were obtained from Boltzmann averaging. RMS, Max Absolute related to the fit between calculated and experimental values, are reported. In addition, cartesian coordinates of the lower energy conformers are displayed in mol2 format. The names that appear in each of the headings are those that appear in the original publication or the name that reflects the revisions carried out.

III.1. Cartesian coordinates of the global minimum conformer of 3β,19α-Dihydroxyursan-28-oic acid (1P)

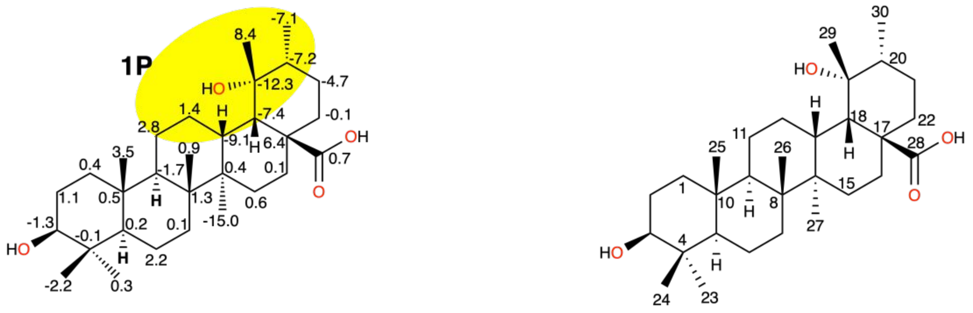

no	δ_{cal}	δ_{exp}	diff	IUPAC no
C17	38.5	38.1	0.4	1
C13	28.1	27.0	1.1	2
C16	77.5	78.8	-1.3	3
C28	38.7	38.8	-0.1	4
C30	54.7	54.5	0.2	5
C19	19.7	17.5	2.2	6
C21	34.3	34.2	0.1	7
C34	42.5	41.2	1.3	8
C29	51.0	52.7	-1.7	9
C33	36.9	36.4	0.5	10
C14	24.0	21.2	2.8	11
C12	32.8	31.4	1.4	12
C25	41.6	50.6	-9.0	13
C31	40.9	40.5	0.4	14
C20	30.3	29.7	0.6	15
C22	26.8	26.7	0.1	16
C35	50.2	43.8	6.4	17
C32	49.7	57.1	-7.4	18
C27	75.2	87.5	-12.3	19
C26	42.4	49.6	-7.2	20
C15	26.3	31.0	-4.7	21
C18	37.6	37.7	-0.1	22
C7	28.1	27.8	0.3	23
C6	16.6	18.8	-2.2	24
C9	18.6	15.1	3.5	25

C10	18.1	17.2	0.9	26
C8	18.2	33.2	-15.0	27
C24	180.1	179.4	0.7	28
C5	28.4	20.0	8.4	29
C4	16.5	23.6	-7.1	30

RMSD	5.1
Max Abs	15.0

mol2 coordinates for lowest energy conformer

1	H1	0.818378928	0.372507821	-1.577546222
2	H2	1.276449942	-1.202222033	-3.068424721
3	H3	-1.134599636	2.039120827	3.024267360
4	C4	1.713704889	3.646655489	5.472439198
5	C5	0.975195455	3.607637529	2.566708378
6	C6	-1.017048074	-2.769858916	-5.167183504
7	C7	1.425906537	-3.079449219	-4.897373690
8	C8	1.914950166	0.007332234	0.700103820
9	C9	-2.167405358	-0.632817835	-3.017311719
10	C10	-1.748924780	-1.372713172	-0.006786720
11	O11	-2.171755937	-1.616061917	3.676971206
12	C12	-0.365556742	1.943804880	0.322916131
13	C13	-0.318771146	0.340144655	-5.353358309
14	C14	-0.931739250	1.414806534	-0.997121364
15	C15	0.642799991	1.403014504	5.739238158
16	C16	0.596179470	-0.850777371	-5.611259054
17	C17	-0.302380979	0.779061657	-3.889575969
18	C18	-0.520495549	0.519538444	5.326687645
19	C19	0.060196961	-2.698398164	-2.204928755
20	C20	0.483409594	-1.456847646	2.090788843
21	C21	0.455879543	-2.231216349	-0.804434392
22	C22	0.559876556	-0.924619076	3.524455244
23	O23	-2.854843784	0.493119067	3.443927954
24	C24	-1.890653217	-0.441082752	3.611852994
25	C25	-0.383616491	0.874223611	1.422235632
26	C26	0.674796725	2.676979127	4.897189000
27	C27	0.905963225	2.318128217	3.392953532
28	C28	0.275514418	-2.071728815	-4.710905893
29	C29	-0.242034794	0.118571370	-1.455009386
30	C30	0.251525595	-1.578902259	-3.232168017
31	C31	0.444581788	-0.374971123	0.976407651
32	C32	-0.258979985	1.388202063	2.902418073
33	C33	-0.655573582	-0.353532181	-2.895520887
34	C34	-0.284767400	-0.963580831	-0.325969265
35	C35	-0.497193402	0.151323630	3.821545664
36	O36	0.625884639	-1.181633950	-6.991332888
37	H37	1.668662899	4.643661488	5.027143260
38	H38	1.546488446	3.768369441	6.547402344
39	H39	2.732522914	3.258058046	5.352878449
40	H40	0.015708331	4.133791425	2.557505628
41	H41	1.722214580	4.291267039	2.982648819
42	H42	1.269480880	3.398818767	1.541376956
43	H43	-1.865420680	-2.090507612	-5.282200568
44	H44	-0.852842474	-3.269872990	-6.128447666
45	H45	-1.313791635	-3.543507317	-4.452303243
46	H46	1.625568627	-3.207113621	-5.965249059

47	H47	2.346872032	-2.724991965	-4.418554902
48	H48	1.184498066	-4.062335772	-4.480711042
49	H49	2.346356684	0.438493930	1.602858186
50	H50	2.509558823	-0.870468542	0.432783366
51	H51	2.047102085	0.739502755	-0.099303861
52	H52	-2.458873929	-1.613351858	-2.639176649
53	H53	-2.744220108	0.116192130	-2.467926060
54	H54	-2.503357188	-0.576937679	-4.054565761
55	H55	-1.876385970	-1.706243764	1.024514064
56	H56	-2.066522950	-2.206696281	-0.636083951
57	H57	-2.469324254	-0.568872718	-0.169418712
58	H58	0.649785648	2.301411393	0.135295328
59	H59	-1.341693786	0.088608923	-5.667470664
60	H60	0.001032665	1.168034510	-5.995695442
61	H61	-2.012108103	1.268848911	-0.884133458
62	H62	-0.811048061	2.193239866	-1.757087877
63	H63	0.539193369	1.660988228	6.800191035
64	H64	1.597406773	0.876334903	5.628432285
65	H65	1.626073492	-0.544619565	-5.383386127
66	H66	-0.994494253	1.619547025	-3.768018798
67	H67	0.700806207	1.161538748	-3.651482910
68	H68	-1.452398072	1.057154741	5.546738113
69	H69	-0.539411912	-0.407056373	5.912127378
70	H70	-0.972770497	-3.065086466	-2.219064324
71	H71	0.684478343	-3.559437390	-2.463395477
72	H72	-0.388096563	-2.109519972	2.037524753
73	H73	1.348362261	-2.106911845	1.917800099
74	H74	0.290374061	-3.042730355	-0.085771905
75	H75	1.535148656	-2.047044618	-0.823313315
76	H76	0.420217252	-1.764298115	4.213592190
77	H77	1.543733063	-0.494215087	3.720818338
78	H78	-3.688142126	-0.000981531	3.389520739
79	H79	-0.311373719	3.161109591	4.967185388
80	H80	-0.285030761	-1.343561845	-7.266758375
81	H81	-0.951296305	2.815020219	0.640850675
82	H82	-1.424687522	0.530880619	1.426593917
83	O83	2.159357964	1.640815224	3.283982126
84	H84	2.857432432	2.300917307	3.362860981

---

11 lowest energy conformers were considered for Boltzmann average to contribute to the ^{13}C NMR data

III.2. Cartesian coordinates of the global minimum conformer of licanolide (2P)

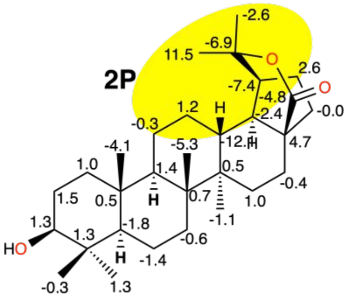

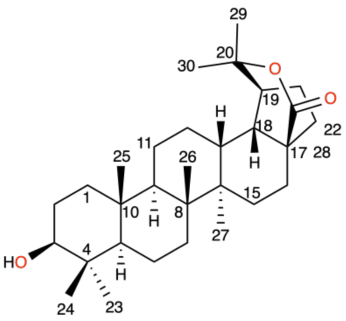

no	δ_{calc}	δ_{exp}	diff	IUPAC no

C30	38.5	37.5	1.0	1	
C32	29.3	27.8	1.5	2	
C36	77.1	75.8	1.3	3	
C31	38.5	37.2	1.3	4	
C29	55.1	56.9	-1.8	5	*
C28	19.5	20.9	-1.4	6	
C26	33.5	34.1	-0.6	7	*
C23	41.1	40.4	0.7	8	
C34	51.2	52.6	-1.4	9	
C27	36.9	36.4	0.5	10	
C24	21.3	21.6	-0.3	11	
C21	27.6	26.4	1.2	12	*
C20	35.9	48.0	-12.1	13	
C1	41.8	41.3	0.5	14	
C17	30.5	29.5	1.0	15	
C12	29.9	30.3	-0.4	16	
C10	48.4	43.7	4.7	17	
C9	47.8	50.2	-2.4	18	
C16	42.0	49.4	-7.4	19	*
C22	80.6	87.5	-6.9	20	
C18	27.4	24.8	2.6	21	
C13	32.5	32.5	0.0	22	
C8	28.1	26.8	1.3	23	
C7	16.7	17.0	-0.3	24	
C6	19.3	23.4	-4.1	25	
C5	15.8	21.1	-5.3	26	
C2	16.2	17.3	-1.1	27	
C14	174.5	179.3	-4.8	28	
C4	30.4	33.0	-2.6	29	
C3	30.4	18.9	11.5	30	
RMSD		4.1			
Max Abs		12.1			

mol2 coordinates for lowest energy conformer

1	O1	-1.336819071	0.719069087	4.843206093
2	C2	1.963207964	-1.070700752	1.033524999
3	C3	-0.555143493	4.171068673	2.103792417
4	C4	0.149406357	4.712717985	4.423575753
5	C5	-1.889582344	-0.426312031	0.418614885
6	C6	-2.067405140	-0.124969534	-2.709138548
7	C7	-2.324062619	-2.873856865	-4.355077610
8	C8	-0.399441536	-4.381315641	-3.923993896
9	O9	-0.318800994	-2.797128490	-6.326486114
10	C10	0.900883094	0.878347556	3.943937749
11	C11	1.384249200	1.510079842	2.629799343
12	C12	0.886129871	-0.643783708	3.849521433
13	C13	1.868086461	1.497039694	4.988777832
14	C14	-0.510118294	1.381135291	4.265144338
15	C15	0.581444906	1.060638171	1.408892248
16	C16	1.485753183	2.984082862	3.073144871
17	C17	0.065886587	-1.097448877	2.633083365
18	C18	2.373866363	2.804758976	4.325215354
19	O19	-0.843361508	2.629394270	3.885295211
20	C20	0.544645853	-0.499511344	1.283474621
21	C21	1.084931783	1.696755159	0.113970411
22	C22	0.097275532	3.605941040	3.367588831

23	C23	-0.448573206	-0.873584516	0.086432812
24	C24	0.206362568	1.319858258	-1.078744488
25	C25	0.071969940	-0.203096318	-1.233542358
26	C26	-0.478534508	-2.404281906	-0.116168747
27	C27	-0.619955537	-0.652589710	-2.572833555
28	C28	-1.157523934	-2.854085762	-1.410442000
29	C29	-0.493622110	-2.208074626	-2.629214281
30	C30	0.205908081	-0.075747105	-3.748931314
31	C31	-0.833076929	-2.903003835	-3.982500226
32	C32	-0.131353772	-0.710061655	-5.097437123
33	C33	0.023020426	-2.225616028	-5.073031724
34	H34	2.422483515	1.184574996	2.479610826
35	H35	-0.455902378	1.377745218	1.564384187
36	H36	1.979469495	3.622780897	2.333325967
37	H37	1.097329394	-0.571083121	-1.363633326
38	H38	0.588027779	-2.392644972	-2.504509201
39	H39	2.328073551	-0.924828900	0.015583295
40	H40	2.702979134	-0.615862095	1.696203915
41	H41	1.988864153	-2.144992098	1.238170962
42	H42	-1.557571880	4.537125417	2.342878984
43	H43	-0.648839403	3.423284958	1.315571432
44	H44	0.037306282	5.007805571	1.719344447
45	H45	0.401738985	4.310940922	5.407973739
46	H46	0.889668194	5.472972066	4.152114480
47	H47	-0.830145137	5.192511226	4.502447405
48	H48	-2.597581949	-0.815298396	-0.311776593
49	H49	-2.020474573	0.658308979	0.435858593
50	H50	-2.211393223	-0.808836581	1.388974631
51	H51	-2.819589501	-0.828798419	-2.347528510
52	H52	-2.317194501	0.089176006	-3.751566608
53	H53	-2.201199060	0.809527181	-2.158051065
54	H54	-2.692073818	-1.871942870	-4.582869292
55	H55	-2.931193264	-3.276101387	-3.537016180
56	H56	-2.494395138	-3.493478663	-5.239254262
57	H57	-0.379074850	-4.795133632	-4.935709964
58	H58	0.602666473	-4.486396830	-3.489447239
59	H59	-1.091296090	-4.986503332	-3.330347867
60	H60	0.240900250	-2.388012403	-6.996234203
61	H61	1.922227261	-0.997274131	3.781415799
62	H62	0.452686259	-1.072646140	4.757160522
63	H63	2.691110627	0.801537357	5.181904854
64	H64	1.371636888	1.676436218	5.947800249
65	H65	-0.970104643	-0.818952587	2.836011752
66	H66	0.088639636	-2.191249689	2.577869722
67	H67	3.410145891	2.682011193	3.994035915
68	H68	2.363837911	3.660843619	5.001175618
69	H69	2.120486925	1.379999634	-0.069976017
70	H70	1.118433919	2.788046371	0.211732567
71	H71	0.640271756	1.753660864	-1.984357388
72	H72	-0.776813198	1.791792736	-0.956538708
73	H73	-0.975455549	-2.878455647	0.738427376
74	H74	0.545344893	-2.794246527	-0.139465321
75	H75	-1.079127660	-3.943748918	-1.471299120
76	H76	-2.230635457	-2.633559099	-1.386799066
77	H77	1.275573008	-0.230866932	-3.544244908
78	H78	0.055415783	1.006563574	-3.824355284

79	H79	0.532879485	-0.291826013	-5.866642358
80	H80	-1.152151873	-0.464220334	-5.411276695
81	H81	1.078362525	-2.456403447	-4.836528641

---

1 lowest energy conformers were considered for Boltzmann average to contribute to the ^{13}C NMR data

III.3. Cartesian coordinates of the global minimum conformer of 11α,12α-epoxy-3β-hydroxyolean-28(13β)-olide (1R)

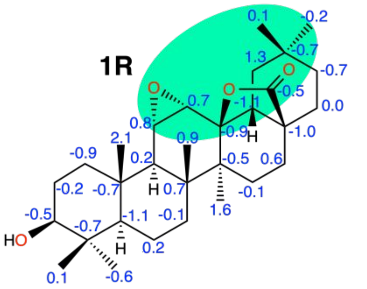

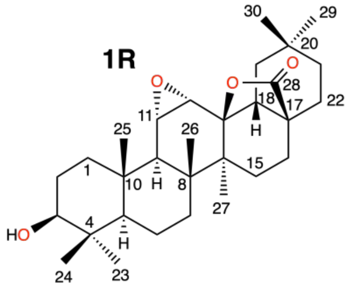

no	δ_{calc}	δ_{exp}	diff	IUPAC no	
C17	37.8	38.7	-0.9	1	
C13	27.4	27.6	-0.2	2	
C16	77.4	77.9	-0.5	3	
C28	38.7	39.4	-0.7	4	
C30	53.9	55.0	-1.1	5	
C19	19.0	18.8	0.2	6	
C21	31.3	31.4	-0.1	7	
C34	41.6	40.9	0.7	8	
C29	51.4	51.2	0.2	9	*
C33	36.1	36.8	-0.7	10	
C14	53.6	52.8	0.8	11	
C12	58.0	57.3	0.7	12	
C25	86.7	87.6	-0.9	13	
C31	41.2	41.7	-0.5	14	
C20	26.9	27.0	-0.1	15	
C22	22.2	21.6	0.6	16	
C35	43.1	44.1	-1.0	17	
C32	48.7	49.8	-1.1	18	*
C27	36.7	38.0	-1.3	19	
C26	30.8	31.5	-0.7	20	
C15	33.7	34.4	-0.7	21	
C18	27.7	27.7	0.0	22	
C7	27.8	28.4	-0.6	23	
C6	16.1	16.0	0.1	24	
C9	19.4	17.3	2.1	25	
C10	21.3	20.4	0.9	26	*
C8	20.5	18.9	1.6	27	*
C24	178.4	178.9	-0.5	28	
C4	32.8	33.0	-0.2	29	
C1	23.5	23.4	0.1	30	
RMSD		0.8			
Max Abs		2.1			

mol2 coordinates for lowest energy conformer

1	H1	0.683106229	-0.470567394	1.291095609
2	H2	1.497908008	0.932784687	2.808213342
3	H3	-1.435219726	-1.976089039	-3.101378725

4	C4	1.989387876	-3.464661634	-4.860288463
5	C5	-0.382817246	2.533888439	5.263520495
6	C6	2.038846604	2.644095004	4.737397835
7	C7	1.500980126	-0.225763170	-0.919003080
8	C8	-1.977921345	0.732048335	3.149003175
9	C9	-1.811111255	1.669461695	0.159402218
10	O10	-2.848785058	1.019078146	-4.027612197
11	C11	-1.477375488	-1.496565162	-0.545241289
12	C12	0.015857316	-0.629292816	5.148152334
13	C13	-1.215306674	-1.242561803	0.875112496
14	C14	0.517774486	-1.580873760	-5.612017945
15	C15	1.069753636	0.448930198	5.378957370
16	C16	-0.170135748	-0.937798581	3.663551679
17	C17	-0.682981088	-0.654833187	-5.359015460
18	C18	0.371535494	2.612620038	2.190806134
19	C19	0.277868817	1.523399525	-2.215300536
20	C20	0.536308381	2.227658114	0.717122131
21	C21	0.388666465	0.917452536	-3.628832168
22	C22	-2.003498745	0.411542465	-3.429584901
23	C23	-0.929622478	-0.587566236	-1.623724189
24	C24	0.651468484	-2.756092255	-4.613876902
25	C25	0.656074937	-2.207919873	-3.163280127
26	C26	0.779889405	1.766522683	4.611658378
27	C27	-0.337464216	-0.078137655	1.318968442
28	C28	0.546611501	1.402264671	3.114482653
29	C29	0.117581106	0.453121941	-1.107993248
30	C30	-0.563616821	-1.327838815	-2.928564057
31	C31	-0.527080647	0.309444353	2.819954453
32	C32	-0.390104075	1.074861980	0.269515911
33	C33	-0.686651243	-0.159343830	-3.916987056
34	O34	-2.109509311	0.138575605	-2.101498082
35	O35	1.285118286	0.662831196	6.764711751
36	O36	-0.558686730	-2.305791825	0.170176543
37	H37	2.121204634	-4.303081454	-4.166355662
38	H38	2.042240077	-3.860924103	-5.881027969
39	H39	2.832375913	-2.777382232	-4.722063913
40	H40	-1.278535712	1.925811581	5.413248631
41	H41	-0.071283165	2.922170994	6.239376294
42	H42	-0.671687633	3.397158973	4.655953559
43	H43	2.362595600	2.664691838	5.782778164
44	H44	2.864492119	2.245690176	4.133668280
45	H45	1.854328737	3.676527786	4.422102627
46	H46	2.093477830	-0.162224410	-1.832674233
47	H47	2.088179287	0.275851388	-0.147600067
48	H48	1.420321142	-1.276623164	-0.639754896
49	H49	-2.135270369	1.810443838	3.087548407
50	H50	-2.694980485	0.265890942	2.467164952
51	H51	-2.264155204	0.427940889	4.159122380
52	H52	-1.913642522	2.303217104	-0.723077120
53	H53	-2.027315200	2.295900398	1.025249576
54	H54	-2.592249216	0.911677616	0.094802697
55	H55	-2.400225021	-1.999820083	-0.838396790
56	H56	-0.935282064	-0.314111219	5.599905483
57	H57	0.322592781	-1.533913346	5.683823472
58	H58	-1.960253934	-1.609073029	1.579830805
59	H59	0.456566974	-1.982465062	-6.631273811

60	H60	1.442568001	-0.988758652	-5.575374144
61	H61	2.031416192	0.067237052	5.009876160
62	H62	-0.936846367	-1.712770749	3.547114124
63	H63	0.764354765	-1.369997269	3.279525615
64	H64	-1.620241018	-1.189701575	-5.553281477
65	H65	-0.663705265	0.191538244	-6.054035199
66	H66	-0.601369992	3.088487258	2.357339228
67	H67	1.119643223	3.375665861	2.424944149
68	H68	-0.563130361	2.222251121	-2.200956553
69	H69	1.173962760	2.119339428	-2.011445265
70	H70	0.344253638	3.106091720	0.089265234
71	H71	1.589145818	1.967207596	0.563838832
72	H72	0.285395864	1.718538022	-4.369148048
73	H73	1.380734390	0.479698336	-3.780990093
74	H74	1.587641912	-1.657321124	-3.023500028
75	H75	0.429311584	0.865393187	7.162158013
76	C76	-0.477192007	-3.777787341	-4.825148894
77	H77	-0.397975614	-4.594996286	-4.098841535
78	H78	-0.411648358	-4.213019538	-5.828870071
79	H79	-1.475999341	-3.341925117	-4.726526999
80	H80	0.669336349	-3.037410173	-2.445898438

---

3 lowest energy conformers were considered for Boltzmann average to contribute to the ^{13}C NMR data

III.4. Cartesian coordinates of the global minimum conformer of 3β-acetoxyurs-11-en-30(13α)-olide (3P)

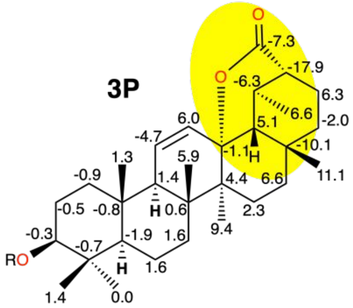

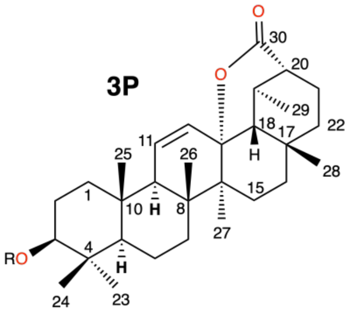

no	δ_{calc}	δ_{exp}	diff	IUPAC no
C33	37.2	38.1	-0.9	1
C35	22.9	23.4	-0.5	2
C36	80.4	80.7	-0.3	3
C34	37.2	37.9	-0.7	4
C32	53.1	55.0	-1.9	5
C30	19.3	17.7	1.6	6
C25	32.9	31.4	1.6	7
C18	42.4	41.8	0.6	8
C24	54.4	53.0	1.4	9
C29	35.6	36.4	-0.8	10
C22	128.7	133.4	-4.7	11
C16	135.0	129.0	6.0	12
C13	88.6	89.7	-1.1	13
C14	46.4	42.0	4.4	14
C19	27.9	25.6	2.3	15
C26	37.5	30.9	6.6	16
C20	35.1	45.2	-10.1	17

C15	45.5	40.4	5.1	18
C21	31.9	38.2	-6.3	19
C28	42.8	60.7	-17.9	20
C31	29.2	22.9	6.3	21
C27	29.3	31.3	-2.0	22
C8	27.8	27.8	0.0	23
C7	17.5	16.1	1.4	24
C6	20.5	19.2	1.3	25
C2	24.9	19.0	5.9	26
C1	25.6	16.2	9.4	27
C3	29.2	18.1	11.1	28
C4	24.5	17.9	6.6	29
C23	172.6	179.9	-7.3	30
C10	171.1	171.1	0.0	3a
C11	20.6	21.4	-0.8	3b
RMSD		5.6		
Max Abs		17.9		

mol2 coordinates for lowest energy conformer.

1	C1	1.798682884	0.252288458	2.046222323
2	C2	-1.731408452	-0.444707057	0.350060453
3	C3	-3.602118631	0.816614520	4.113095596
4	C4	-2.284903759	4.211848402	1.746459857
5	O5	1.147763807	4.121754401	4.149335145
6	C6	-0.932558377	-0.102180536	-2.614511919
7	C7	-0.048043914	-2.830795941	-4.048291725
8	C8	1.929899953	-3.862767068	-2.956111259
9	O9	2.396583802	-2.248134601	-5.323463037
10	C10	3.581349580	-2.050184733	-5.926741447
11	C11	3.622641539	-2.757672656	-7.257150606
12	O12	4.480789429	-1.394822524	-5.457204482
13	C13	-0.315550939	1.631762620	2.078807866
14	C14	0.250023243	0.164678189	1.981345749
15	C15	-1.774241704	1.755649417	2.610553613
16	C16	-0.102394237	2.318426698	0.739589546
17	O17	0.580986133	2.340761776	2.991406381
18	C18	-0.206458852	-0.502481735	0.592256255
19	C19	-0.154471865	-0.664984097	3.222152780
20	C20	-2.080145537	0.888556914	3.884895484
21	C21	-2.154509659	3.222925323	2.914988654
22	C22	0.269062233	1.729944440	-0.396826166
23	C23	0.257426080	3.449089730	3.692514584
24	C24	0.549859821	0.260691403	-0.531769586
25	C25	0.186443722	-1.994668448	0.527890667
26	C26	-1.597659830	-0.559824788	3.695251878
27	C27	-1.400537381	1.465985307	5.152549007
28	C28	-1.205820298	3.713773994	4.008657014
29	C29	0.463981596	-0.303359002	-1.987713994
30	C30	0.117105041	-2.584743565	-0.886932331
31	C31	-1.538819386	2.980326299	5.321971131
32	C32	0.954375970	-1.782833246	-1.888448728
33	C33	1.490674898	0.463817987	-2.856142846
34	C34	1.219552813	-2.524257837	-3.235151336
35	C35	1.752148845	-0.201279187	-4.207397324
36	C36	2.202598859	-1.642407041	-4.030395807
37	H37	-2.435083674	1.402360139	1.818162762

38	H38	1.616328846	0.142854685	-0.299785486
39	H39	-1.296322985	4.792833250	4.162789099
40	H40	1.955878202	-1.699771186	-1.431501852
41	H41	2.115226992	0.516661963	3.054943611
42	H42	2.256388139	-0.709557815	1.798644500
43	H43	2.213602323	1.017395165	1.388302648
44	H44	-2.018987491	-1.139775495	-0.437844722
45	H45	-2.071685791	0.544024478	0.038539081
46	H46	-2.294784813	-0.739231278	1.232760087
47	H47	-4.125618986	0.543027653	3.189107227
48	H48	-3.824092967	0.046881791	4.861099356
49	H49	-4.035119203	1.751632566	4.479569148
50	H50	-2.637240777	3.722716154	0.832572702
51	H51	-1.348576910	4.727713227	1.516436403
52	H52	-3.008212233	4.990025029	2.012352095
53	H53	-1.616969588	-0.932896698	-2.433259227
54	H54	-1.396789849	0.805324304	-2.214737151
55	H55	-0.872764722	0.027319024	-3.697643720
56	H56	-0.780788571	-3.362201134	-3.432118929
57	H57	0.202533207	-3.472718747	-4.897539157
58	H58	-0.530792681	-1.936403470	-4.445509412
59	H59	1.241522021	-4.612669491	-2.555295856
60	H60	2.753958967	-3.738801974	-2.243184186
61	H61	2.342736318	-4.265978336	-3.885754181
62	H62	2.830964506	-2.373341817	-7.906873808
63	H63	4.595086604	-2.600302103	-7.723032958
64	H64	3.442963872	-3.827622133	-7.118000793
65	H65	-0.178055291	3.397567474	0.750514738
66	H66	0.107415262	-1.714945589	3.059574669
67	H67	0.488154162	-0.341042402	4.047034043
68	H68	-3.154033002	3.180803785	3.357355490
69	H69	0.443031762	2.368655923	-1.259146796
70	H70	-0.470700229	-2.579420082	1.182543965
71	H71	1.207244352	-2.140588656	0.898919702
72	H72	-2.279026792	-1.090506824	3.023842168
73	H73	-1.684434830	-1.075893425	4.661156197
74	H74	-1.818493961	0.957350505	6.030367298
75	H75	-0.333639324	1.221955776	5.138523720
76	H76	-0.922254049	-2.656153136	-1.227104263
77	H77	0.482149116	-3.614591800	-0.833309989
78	H78	-2.563381916	3.250074589	5.606015869
79	H79	-0.880945214	3.328729551	6.124575341
80	H80	1.153266697	1.490919108	-3.033240282
81	H81	2.441786072	0.537529358	-2.310352998
82	H82	0.859685748	-0.176110126	-4.842865932
83	H83	2.537039805	0.344930509	-4.739684065
84	H84	3.169525452	-1.645554100	-3.513667545

-----

1 lowest energy conformers were considered for Boltzmann average to contribute to the ^{13}C NMR data

III.5. Cartesian coordinates of the global minimum conformer of 3β-acetoxyurs-11-en-28(13β)-olide (3R)

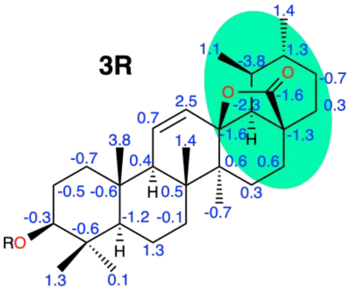

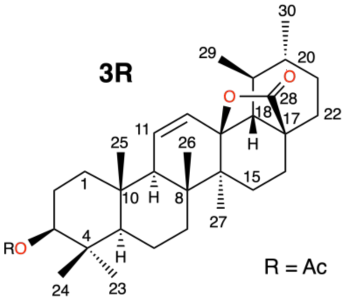

no	δ_{calc}	δ_{exp}	diff	IUPAC no	
C12	37.4	38.1	-0.7	1	
C11	22.9	23.4	-0.5	2	
C22	80.4	80.7	-0.3	3	
C25	37.3	37.9	-0.6	4	
C20	53.8	55.0	-1.2	5	
C9	19.0	17.7	1.3	6	
C13	31.3	31.4	-0.1	7	
C27	42.3	41.8	0.5	8	
C21	53.4	53.0	0.4	9	
C26	35.8	36.4	-0.6	10	
C10	134.1	133.4	0.7	11	
C15	131.5	129.0	2.5	12	
C30	88.1	89.7	-1.6	13	
C28	42.6	42.0	0.6	14	
C16	25.9	25.6	0.3	15	
C17	23.5	22.9	0.6	16	*
C29	43.9	45.2	-1.3	17	
C23	58.4	60.7	-2.3	18	*
C19	36.6	40.4	-3.8	19	*
C18	39.5	38.2	1.3	20	*
C8	30.2	30.9	-0.7	21	*
C14	31.6	31.3	0.3	22	
C4	27.9	27.8	0.1	23	
C3	17.4	16.1	1.3	24	
C5	20.0	16.2	3.8	25	*
C6	20.6	19.2	1.4	26	*
C7	18.3	19.0	-0.7	27	*
C24	178.3	179.9	-1.6	28	
C2	19.0	17.9	1.1	29	*
C1	19.5	18.1	1.4	30	*
C31	171.2	171.1	0.1	31	
C32	20.5	21.4	-0.9	32	
RMSD		1.4			
Max Abs		3.8			

(The chemical shifts come from the compound 3P)

mol2 coordinates for lowest energy conformer

1	H1	0.066075544	0.826383501	0.553513127
2	H2	-1.609243014	2.217524414	0.209318830
3	H3	3.200537985	-3.184189094	-0.171073708
4	C4	6.696892726	-1.299624959	-1.115561007
5	C5	4.344774431	-0.932318293	0.769721353
6	C6	-4.926454587	1.593229037	1.074694257
7	C7	-3.884066165	3.530292537	-0.082583863
8	C8	-2.801922450	-0.743221918	1.659957718

9	C9	-1.490559195	-1.935360018	-0.894198007
10	C10	1.662224417	0.440783922	-1.164963636
11	O11	1.274705907	-4.851637592	-2.493193555
12	O12	-4.214889048	3.711870981	2.791793603
13	O13	0.894594836	-3.289127099	-0.929694259
14	C14	0.274240711	-1.048490814	1.463615029
15	C15	1.182636312	-1.886880561	0.964791491
16	C16	5.248810654	-2.721460272	-2.597110465
17	C17	-2.594259663	1.967200133	3.216510404
18	C18	-1.456094766	1.142097317	2.616893485
19	C19	4.029947335	-3.639270564	-2.725234983
20	C20	-2.687509743	0.917045095	-1.060680189
21	C21	-1.394826019	0.400506149	-1.702460382
22	C22	0.907525433	-1.251496835	-2.829796114
23	C23	2.272187665	-1.843019458	-3.230217698
24	C24	1.599993618	-3.828676274	-1.951823074
25	C25	5.435620160	-2.164706037	-1.175307860
26	C26	4.180025736	-1.398144325	-0.680583368
27	C27	-3.118977742	2.978718727	2.210927788
28	C28	-3.609668974	2.363340480	0.885195333
29	C29	-0.535520576	-0.090601279	0.625164542
30	C30	-2.434762689	1.495009063	0.336527993
31	C31	2.972507625	-2.332495621	-0.834244337
32	C32	-1.848350940	0.418862125	1.306223538
33	C33	-0.655407882	-0.636168401	-0.830841400
34	C34	0.838048280	-0.859126099	-1.336057461
35	C35	1.488351926	-2.009169079	-0.499067862
36	C36	2.778640833	-2.920651600	-2.240928947
37	H37	6.565401977	-0.380650984	-1.700775201
38	H38	7.553853927	-1.839368001	-1.532653362
39	H39	6.955673361	-1.013755347	-0.092041098
40	H40	3.418445741	-0.508635127	1.167617772
41	H41	4.619519729	-1.778258673	1.413631293
42	H42	5.124899333	-0.171226235	0.862637335
43	H43	-5.732202051	2.290169725	1.321370985
44	H44	-4.878580935	0.850376349	1.872684107
45	H45	-5.204123202	1.074313572	0.151435041
46	H46	-2.954416189	4.027279144	-0.384817189
47	H47	-4.523984619	4.272933389	0.403355843
48	H48	-4.402513718	3.197491116	-0.986126597
49	H49	-3.382053985	-0.528165499	2.560691903
50	H50	-3.518112338	-0.971395504	0.869200271
51	H51	-2.232268079	-1.656092225	1.859086551
52	H52	-2.553147712	-1.710427501	-0.803659251
53	H53	-1.359912718	-2.445617436	-1.850053210
54	H54	-1.230608419	-2.649931238	-0.115177200
55	H55	2.490163464	0.476819209	-1.873410215
56	H56	2.083563057	0.552780952	-0.163634420
57	H57	1.057120482	1.326618833	-1.366612920
58	H58	0.127312268	-1.039388892	2.540619646
59	H59	1.735606849	-2.552783415	1.623832422
60	H60	5.166221554	-1.881892923	-3.301582375
61	H61	6.151415128	-3.272633120	-2.886251290
62	H62	-2.232905965	2.505616119	4.097954210
63	H63	-3.417554669	1.325999597	3.550503532
64	H64	-1.115959498	0.420199230	3.367447281

65	H65	-0.605899033	1.810133702	2.418716554
66	H66	4.184171783	-4.536524298	-2.112521856
67	H67	3.899628072	-3.983289349	-3.756820633
68	H68	-3.100946460	1.688350097	-1.716661327
69	H69	-3.442742498	0.125235286	-1.018407288
70	H70	-1.625886893	-0.045290804	-2.677391938
71	H71	-0.761833810	1.271730080	-1.904761847
72	H72	0.129070883	-1.980099672	-3.072570941
73	H73	0.702779613	-0.369230237	-3.446325086
74	H74	3.024965191	-1.052073402	-3.314518565
75	H75	2.185970573	-2.296934336	-4.223771234
76	H76	5.577374307	-3.024147577	-0.500431671
77	H77	4.077206972	-0.517952479	-1.321665555
78	H78	-2.320837512	3.696982644	1.989451051
79	C79	-3.912545900	4.778644307	3.551366047
80	O80	-2.786808122	5.149116174	3.783676104
81	C81	-5.169380765	5.439618690	4.057054803
82	H82	-5.763939379	5.798921858	3.212267316
83	H83	-4.907229301	6.274467642	4.706386406
84	H84	-5.779799181	4.714939272	4.602429485

---

1 lowest energy conformers were considered for Boltzmann average to contribute to the ^{13}C NMR data

III.6. Cartesian coordinates of the global minimum conformer of urs-21-en-3β,20,28-triol (4P)

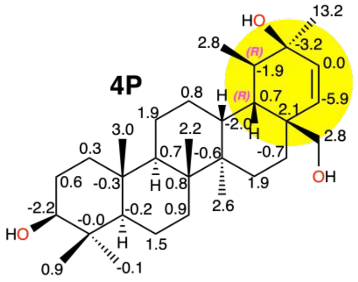

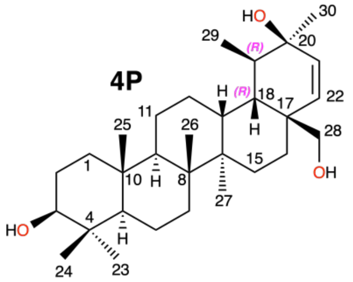

no	δ_{calc}	δ_{exp}	diff	IUPAC no
C12	39.2	38.9	0.3	1
C11	28.1	27.5	0.6	2
C23	77.2	79.4	-2.2	3
C25	38.8	38.8	0.0	4
C21	55.2	55.4	-0.2	5
C10	19.7	18.2	1.5	6
C13	34.9	34.0	0.9	7
C28	41.4	40.6	0.8	8
C22	51.3	50.6	0.7	9
C26	36.9	37.2	-0.3	10
C9	23.2	21.3	1.9	11
C8	28.1	27.3	0.8	12
C20	36.9	38.9	-2.0	13
C27	41.0	41.6	-0.6	14
C14	28.4	26.5	1.9	15
C16	26.6	27.3	-0.7	16
C30	39.7	37.6	2.1	17
C24	46.1	45.4	0.7	18
C19	42.3	44.2	-1.9	19
C29	71.0	74.2	-3.2	20

C15	133.0	133.0	0.0	21
C17	134.7	140.6	-5.9	22
C2	27.9	28.0	-0.1	23
C3	16.2	15.3	0.9	24
C4	19.3	16.3	3.0	25
C6	17.9	15.7	2.2	26
C5	16.9	14.3	2.6	27
C18	68.7	65.9	2.8	28
C1	24.7	21.9	2.8	29
C7	35.4	22.2	13.2	30
RMSD		3.1		
Max Abs		13.2		

mol2 coordinates for lowest energy conformer

1	H1	-1.320581646	2.608515790	1.522404039
2	H2	1.764919807	-1.489697706	-0.869324148
3	H3	-0.670524455	1.357559317	-0.261454094
4	H4	1.284615696	-2.796378710	-2.613951196
5	C5	-0.245916106	-2.368702496	-4.346592559
6	C6	-2.978606116	-3.799590369	-2.131740306
7	C7	-1.623048734	4.169955888	3.625510913
8	C8	0.765275340	4.752345513	3.303127145
9	C9	2.100432814	3.048095674	0.964961163
10	C10	-1.564819728	-1.102566257	-0.012004181
11	C11	1.980669038	-0.149510222	1.418054986
12	O12	2.192574984	-5.279315079	0.143029666
13	O13	-1.126682710	6.538223709	1.964602803
14	O14	-2.168194817	-4.193720562	-4.333650384
15	C15	-0.969832167	-5.179461302	-2.607377030
16	C16	0.008797273	-5.195209058	-1.710952727
17	C17	2.045142741	-4.214345225	-0.777239954
18	C18	-0.047857014	5.202627773	0.249598635
19	C19	0.108854335	3.803467775	-0.346580611
20	C20	0.656302415	-0.136127476	-2.016519912
21	C21	1.166455742	1.095499042	-1.269430881
22	C22	-0.011784989	1.696682164	2.902555975
23	C23	-0.306867331	0.346616444	2.249894283
24	C24	0.213680999	-2.487286380	1.047829287
25	C25	-0.179465382	-3.776534436	0.329774104
26	C26	-0.800331543	-2.660937661	-2.942140076
27	C27	-0.948669349	5.216728816	1.478851767
28	C28	-1.716860406	-3.937534441	-3.000207443
29	C29	-0.489981832	4.230775240	2.583916585
30	C30	-0.315281014	2.831742763	1.920719517
31	C31	0.708474878	-1.393440748	-1.144881509
32	C32	0.387520018	1.325196498	0.030575893
33	C33	0.418201615	-2.709919150	-1.942427979
34	C34	0.612371987	2.747359341	0.669542574
35	C35	-0.042892170	-1.212256581	0.210899293
36	C36	0.518242417	0.080291861	0.972233485
37	C37	0.540747960	-3.962642678	-1.023660729
38	H38	0.429153632	-1.510811707	-4.321410164
39	H39	0.310710214	-3.233053807	-4.722398971
40	H40	-1.033161655	-2.144940104	-5.070049342
41	H41	-3.550392698	-2.907838974	-2.423937661
42	H42	-2.752494657	-3.719904165	-1.067888757

43	H43	-3.611348704	-4.680296436	-2.277802058
44	H44	-2.496903466	3.636041781	3.232953915
45	H45	-1.933760978	5.187167141	3.880650779
46	H46	-1.308880392	3.673384953	4.548701865
47	H47	0.511984922	5.631503493	3.907210698
48	H48	1.162028402	3.997333716	3.990052457
49	H49	1.573621632	5.031562821	2.622318095
50	H50	2.324272649	4.111599422	0.846686656
51	H51	2.404038274	2.778141362	1.979331227
52	H52	2.756560791	2.517326998	0.269905805
53	H53	-1.915972792	-1.888393066	-0.676293473
54	H54	-1.871214686	-0.153645747	-0.460023382
55	H55	-2.115136605	-1.220966822	0.926271667
56	H56	2.430666186	0.766251423	1.797716968
57	H57	2.027439389	-0.879810211	2.231033326
58	H58	2.630165470	-0.509476631	0.615662790
59	H59	3.130290004	-5.490366921	0.209127372
60	H60	-0.249590642	6.905476304	2.128555659
61	H61	-2.801815918	-3.502323113	-4.564820345
62	H62	-1.328911998	-6.098749252	-3.064210877
63	H63	0.461545535	-6.138151668	-1.414113737
64	H64	2.537813318	-3.310743019	-0.392905319
65	H65	2.511732040	-4.463193261	-1.743723112
66	H66	-0.472362665	5.880764649	-0.498648192
67	H67	0.933766207	5.620156131	0.514609057
68	H68	0.788259705	3.864139861	-1.203605677
69	H69	-0.865520220	3.481284178	-0.741218087
70	H70	-0.363180565	0.054459513	-2.376633656
71	H71	1.277723105	-0.288147979	-2.906337575
72	H72	1.083678367	1.964028988	-1.930189758
73	H73	2.237006981	0.967129640	-1.064236866
74	H74	-0.638156530	1.788030791	3.794878038
75	H75	1.025597850	1.743178168	3.254846574
76	H76	-0.141618667	-0.458283877	2.975491110
77	H77	-1.375937537	0.338810228	2.012753385
78	H78	1.272778504	-2.559057016	1.312527591
79	H79	-0.336088168	-2.431151331	1.994789877
80	H80	-1.259680774	-3.806459956	0.170320559
81	H81	0.057312178	-4.633111321	0.967925217
82	H82	-1.446872582	-1.820017270	-2.669167964
83	H83	-1.954225006	4.904615021	1.167347895

---

3 lowest energy conformers were considered for Boltzmann average to contribute to the ^{13}C NMR data

III.7. The four stereoisomers of ursane 4P and the revised structure 4R

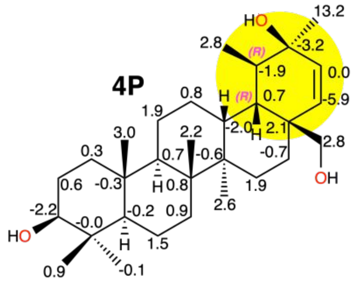

Max absolute = 13.2 ; rms = 3.1
urs-21-en-3β,20β,28-triol

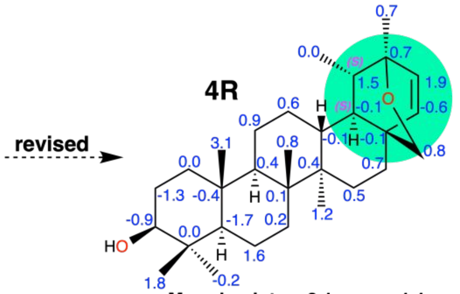

Max absolute = 3.1; rms = 1.1
20β,28-epoxytaraxast-21-en-3β-ol

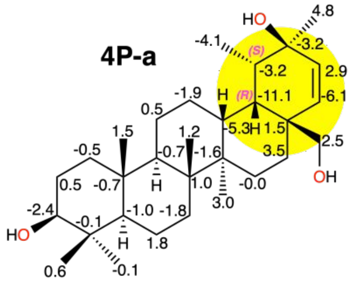

Max absolute = 11.1; rms = 3.2

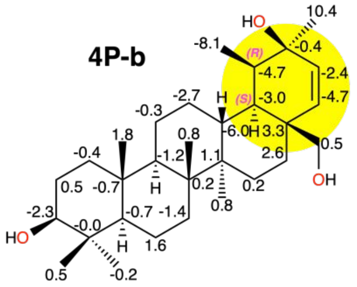

Max absolute = 10.4; rms = 3.2

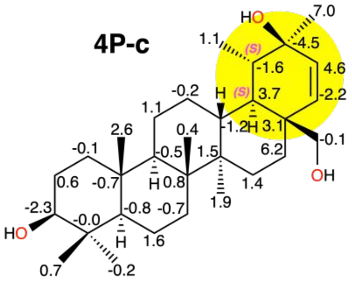

Max absolute = 6.9; rms = 2.5

III.8. Cartesian coordinates of the global minimum conformer of 4P-a

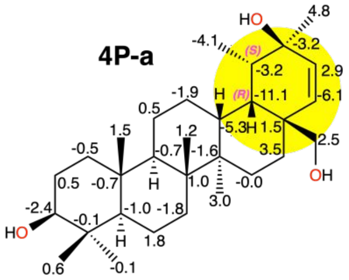

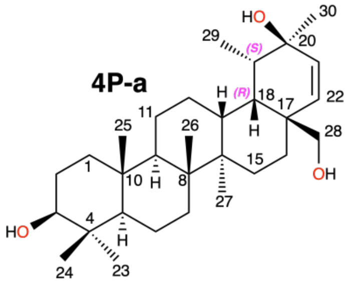

no	δ_{calc}	δ_{exp}	diff	IUPAC no
C12	38.4	38.9	-0.5	1
C11	28.0	27.5	0.5	2
C23	77.0	79.4	-2.4	3
C25	38.7	38.8	-0.1	4
C21	54.4	55.4	-1.0	5
C10	20.0	18.2	1.8	6
C13	32.2	34.0	-1.8	7
C28	41.6	40.6	1.0	8
C22	49.9	50.6	-0.7	9
C26	36.5	37.2	-0.7	10
C9	21.8	21.3	0.5	11
C16	30.8	27.3	3.5	12
C20	33.6	38.9	-5.3	13
C27	40.0	41.6	-1.6	14
C14	26.5	26.5	0.0	15
C8	25.4	27.3	-1.9	16
C30	39.1	37.6	1.5	17
C24	34.3	45.4	-11.1	18
C19	41.0	44.2	-3.2	19

C29	71.0	74.2	-3.2	20
C15	135.9	133.0	2.9	21
C17	134.5	140.6	-6.1	22
C2	27.9	28.0	-0.1	23
C3	15.9	15.3	0.6	24
C4	17.8	16.3	1.5	25
C6	16.9	15.7	1.2	26
C5	17.3	14.3	3.0	27
C18	68.4	65.9	2.5	28
C1	17.8	21.9	-4.1	29
C7	27.0	22.2	4.8	30
RMSD		3.2		
Max Abs		11.1		

mol2 coordinates for lowest energy conformer

1	H1	-1.090943411	2.707612895	1.884338495
2	H2	1.389080492	-1.380750363	-0.926609174
3	H3	-0.932832955	1.442639130	0.069618122
4	H4	0.910140320	-2.809607733	-2.424586018
5	C5	-2.541451014	-2.699571177	-2.286447230
6	C6	-1.928578771	-4.427279892	-4.664816971
7	C7	-0.895564132	4.323788082	3.947748802
8	C8	1.372671632	4.847659932	3.096784807
9	C9	2.108543378	3.052602079	0.528599758
10	C10	-1.768628221	-0.895975900	0.404498537
11	C11	2.068116698	-0.064391678	0.974106762
12	O12	2.211066473	-4.673685161	-1.865713885
13	O13	-0.727301079	6.660414702	2.180088329
14	O14	0.361301130	-4.220058632	-4.211218252
15	C15	-0.954522616	-5.351881727	-2.550098236
16	C16	-0.461181223	-5.210644469	-1.324366610
17	C17	1.675318529	-4.257439377	-0.616549554
18	C18	-0.081346854	5.280114709	0.292043135
19	C19	-0.104395665	3.872641899	-0.300819681
20	C20	0.017905515	-0.177091100	-1.976346354
21	C21	0.556422506	1.142326524	-1.413580241
22	C22	0.517151308	1.824240541	2.935266089
23	C23	0.121211579	0.441854162	2.415513541
24	C24	0.023826523	-2.365264842	1.248682617
25	C25	-0.444471388	-3.665667387	0.594533261
26	C26	-1.110449252	-2.869651037	-2.796450382
27	C27	-0.689872039	5.329913564	1.687720254
28	C28	-0.907288281	-4.218089730	-3.549514023
29	C29	-0.019903247	4.344934941	2.680877423
30	C30	-0.018980561	2.931633393	2.024238781
31	C31	0.300710917	-1.368400050	-1.058105581
32	C32	0.163397404	1.392523689	0.053543157
33	C33	0.039088572	-2.735728627	-1.766809078
34	C34	0.583259141	2.809784637	0.589642993
35	C35	-0.241433218	-1.113903856	0.380649394
36	C36	0.536758769	0.166281979	0.951748095
37	C37	0.173053479	-3.944587521	-0.790903644
38	H38	-2.694522950	-1.698727025	-1.881760581
39	H39	-3.261832871	-2.821483569	-3.100091336
40	H40	-2.792119553	-3.428898213	-1.508626999
41	H41	-1.951749916	-3.556983224	-5.328700481

42	H42	-2.935232545	-4.603888107	-4.277459284
43	H43	-1.627584329	-5.298048160	-5.253866640
44	H44	-1.839902707	3.794931016	3.769238119
45	H45	-1.130828173	5.351242161	4.240138047
46	H46	-0.390684152	3.841257613	4.790905353
47	H47	1.275057171	5.743482843	3.719527850
48	H48	1.893366760	4.095743924	3.698372838
49	H49	2.018645547	5.092911631	2.249552290
50	H50	2.337384883	4.109514456	0.369742667
51	H51	2.632044573	2.752479829	1.438569677
52	H52	2.564479855	2.511301190	-0.304222956
53	H53	-2.288499032	-1.823443424	0.168560297
54	H54	-2.120392991	-0.147057208	-0.307127804
55	H55	-2.116987480	-0.591872842	1.394615630
56	H56	2.554397522	0.649172045	1.640262755
57	H57	2.320564715	-1.056861521	1.355752069
58	H58	2.548837229	0.041541468	-0.000001690
59	H59	3.158948347	-4.513279236	-1.870885711
60	H60	0.172304042	7.005234283	2.141570238
61	H61	1.030000699	-4.479695946	-3.557526243
62	H62	-1.360335537	-6.307381304	-2.876265190
63	H63	-0.462664430	-6.055529833	-0.634354472
64	H64	1.798508601	-5.056168204	0.130894805
65	H65	2.207707708	-3.369292864	-0.250608475
66	H66	-0.639165181	5.966904997	-0.353821579
67	H67	0.947638388	5.665505490	0.329970876
68	H68	0.365417416	3.900003312	-1.289892794
69	H69	-1.152127852	3.579879777	-0.460221168
70	H70	-1.058499682	-0.086610853	-2.162698311
71	H71	0.477298516	-0.356310319	-2.956549939
72	H72	0.189955179	1.959328243	-2.042456676
73	H73	1.647049802	1.146537766	-1.524655742
74	H74	0.108489679	1.934228711	3.944516982
75	H75	1.604362167	1.904664941	3.044682046
76	H76	0.552620934	-0.329362574	3.065730236
77	H77	-0.964607568	0.365496023	2.527106715
78	H78	1.088195349	-2.462852383	1.482963783
79	H79	-0.486086819	-2.257044551	2.213719916
80	H80	-1.534470998	-3.669894353	0.491485741
81	H81	-0.209045163	-4.505640219	1.262672774
82	H82	-0.939350888	-2.095405137	-3.555360480
83	H83	-1.746464701	5.039072754	1.609295412

---

3 lowest energy conformers were considered for Boltzmann average to contribute to the ^{13}C NMR data

III.9. Cartesian coordinates of the global minimum conformer of 4P-b

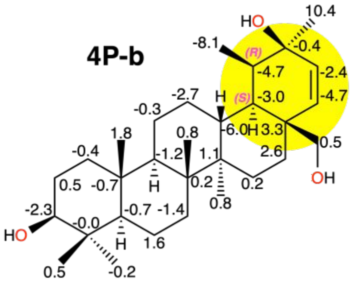

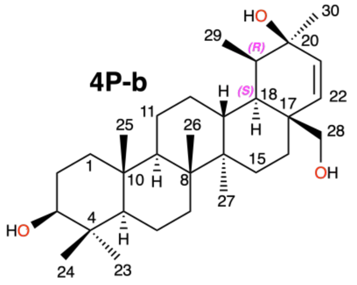

no	δ_{calc}	δ_{exp}	diff	IUPAC no
C12	38.5	38.9	-0.4	1
C11	28.0	27.5	0.5	2
C23	77.1	79.4	-2.3	3
C25	38.8	38.8	0.0	4
C21	54.7	55.4	-0.7	5
C10	19.8	18.2	1.6	6
C13	32.6	34.0	-1.4	7
C28	40.8	40.6	0.2	8
C22	49.4	50.6	-1.2	9
C26	36.5	37.2	-0.7	10
C9	21.0	21.3	-0.3	11
C8	24.6	27.3	-2.7	12
C20	32.9	38.9	-6.0	13
C27	42.7	41.6	1.1	14
C14	26.7	26.5	0.2	15
C16	29.9	27.3	2.6	16
C30	40.9	37.6	3.3	17
C24	42.4	45.4	-3.0	18
C19	39.5	44.2	-4.7	19
C29	73.8	74.2	-0.4	20
C15	130.6	133.0	-2.4	21
C17	135.9	140.6	-4.7	22
C2	27.8	28.0	-0.2	23
C3	15.8	15.3	0.5	24
C4	18.1	16.3	1.8	25
C6	16.5	15.7	0.8	26
C5	15.1	14.3	0.8	27
C18	66.4	65.9	0.5	28
C1	13.8	21.9	-8.1	29
C7	32.6	22.2	10.4	30
RMSD		3.2		
Max Abs		10.4		

mol2 coordinates for lowest energy conformer

1	H1	-2.668153303	-1.191992136	2.000163607
2	H2	1.420617464	1.172452378	-1.093143031
3	H3	-1.423524554	-1.129742753	0.177727136
4	H4	2.720591021	-1.522320814	-1.450153152
5	C5	2.989095761	1.105594499	-3.704808907
6	C6	3.884379272	-2.649462249	-3.623434251
7	C7	-4.253560442	-0.873123216	4.075935821
8	C8	-4.803711575	1.333002260	3.085699663
9	C9	-3.050151792	1.920726103	0.461649776
10	C10	0.945259309	-1.905874131	0.456715199
11	C11	0.066796950	1.937107149	0.854436355
12	O12	5.004953826	1.924100498	0.300506496
13	O13	-6.610537367	-0.847246905	2.333699973
14	O14	4.690624307	-0.739601725	-4.833345119
15	C15	5.335631593	-0.912576599	-2.596439620
16	C16	5.212533321	-0.409271960	-1.370829030
17	C17	4.012005473	1.616615113	-0.667606819
18	C18	-5.265356077	-0.298353500	0.389475129
19	C19	-3.866159310	-0.343418201	-0.222248061
20	C20	0.169959687	-0.293453168	-1.960959990
21	C21	-1.154873608	0.249865867	-1.410823356

22	C22	-1.772781237	0.474893426	2.941941017
23	C23	-0.396810427	0.060944746	2.419852863
24	C24	2.386591101	-0.069768527	1.261094481
25	C25	3.718623449	-0.505536036	0.639717880
26	C26	2.938655552	-0.324292587	-3.154731274
27	C27	-5.288158616	-0.821535060	1.820376981
28	C28	4.195465776	-1.145668152	-3.556869837
29	C29	-4.297441126	-0.080903792	2.755916476
30	C30	-2.893274223	-0.114328962	2.080360202
31	C31	1.374148789	0.078934934	-1.089551964
32	C32	-1.382450137	-0.038023146	0.082708655
33	C33	2.723121578	-0.440835289	-1.629662037
34	C34	-2.796059095	0.403505902	0.610492193
35	C35	1.135328574	-0.369609627	0.391638045
36	C36	-0.148329878	0.406619154	0.935050816
37	C37	3.912523239	0.075998341	-0.775384981
38	H38	2.252767252	1.747400859	-3.208257078
39	H39	3.979415062	1.549843832	-3.581667137
40	H40	2.771125648	1.119271792	-4.775955504
41	H41	3.113001718	-2.846141714	-4.380229935
42	H42	3.525031787	-3.039454597	-2.667526713
43	H43	4.786775513	-3.202193804	-3.901071203
44	H44	-3.726019947	-1.826554571	3.949860816
45	H45	-5.274878888	-1.091013565	4.401293755
46	H46	-3.757412287	-0.314737843	4.876185534
47	H47	-5.696178585	1.273715169	3.718010630
48	H48	-4.051379119	1.892870614	3.649030451
49	H49	-5.054920495	1.922786261	2.201096245
50	H50	-4.110797523	2.134196776	0.304324392
51	H51	-2.739795075	2.498944334	1.334223840
52	H52	-2.522570013	2.328567121	-0.404663412
53	H53	1.839450376	-2.424157127	0.102664966
54	H54	0.113018230	-2.282273011	-0.139126337
55	H55	0.788450549	-2.240649387	1.485347153
56	H56	-0.646857182	2.466336963	1.487520554
57	H57	1.060546192	2.222214239	1.209504616
58	H58	-0.046802330	2.341486982	-0.153871401
59	H59	5.162604121	2.873273601	0.264960112
60	H60	-6.973983841	0.041976941	2.238847301
61	H61	4.024840621	-0.983237053	-5.489301511
62	H62	6.310551498	-1.218767189	-2.969782270
63	H63	6.095927429	-0.286522020	-0.748374225
64	H64	3.049851877	2.057593165	-0.372827224
65	H65	4.285883731	2.036719758	-1.640424403
66	H66	-5.955016046	-0.901133000	-0.211463464
67	H67	-5.660763309	0.726963426	0.369777012
68	H68	-3.912928390	0.068613810	-1.236253717
69	H69	-3.565674558	-1.396125014	-0.325624584
70	H70	0.113174464	-1.383059360	-2.080779087
71	H71	0.301754899	0.112451709	-2.970055476
72	H72	-1.969758258	-0.179167260	-2.001751225
73	H73	-1.186186705	1.328879058	-1.601667587
74	H74	-1.863158905	0.120082247	3.973213191
75	H75	-1.856809645	1.566416613	2.995263189
76	H76	0.382357976	0.524484272	3.036968582
77	H77	-0.309696744	-1.020177655	2.572270983

78	H78	2.465348352	0.996536512	1.487902088
79	H79	2.273810323	-0.570019093	2.230020681
80	H80	3.783995454	-1.598496214	0.576396747
81	H81	4.536451657	-0.179555158	1.288129772
82	H82	2.083677021	-0.824739651	-3.629000483
83	H83	-4.983797183	-1.876873603	1.801694032

---

2 lowest energy conformers were considered for Boltzmann average to contribute to the ^{13}C NMR data

III.10. Cartesian coordinates of the global minimum conformer of 4P-c

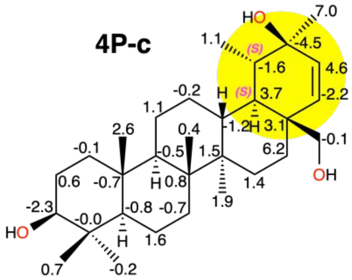

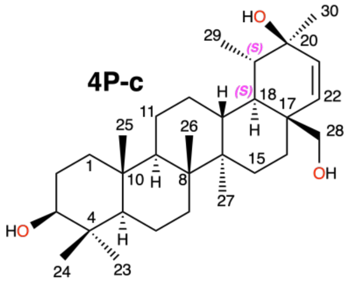

no	δ_{calc}	δ_{exp}	diff	IUPAC no
C12	38.8	38.9	-0.1	1
C11	28.1	27.5	0.6	2
C23	77.1	79.4	-2.3	3
C25	38.8	38.8	0.0	4
C21	54.6	55.4	-0.8	5
C10	19.8	18.2	1.6	6
C13	33.3	34.0	-0.7	7
C28	41.4	40.6	0.8	8
C22	50.1	50.6	-0.5	9
C26	36.5	37.2	-0.7	10
C9	22.4	21.3	1.1	11
C8	27.1	27.3	-0.2	12
C20	37.7	38.9	-1.2	13
C27	43.1	41.6	1.5	14
C14	27.9	26.5	1.4	15
C16	33.5	27.3	6.2	16
C30	40.7	37.6	3.1	17
C24	49.1	45.4	3.7	18
C19	42.6	44.2	-1.6	19
C29	69.7	74.2	-4.5	20
C15	137.6	133.0	4.6	21
C17	138.4	140.6	-2.2	22
C2	27.8	28.0	-0.2	23
C3	16.0	15.3	0.7	24
C4	18.9	16.3	2.6	25
C6	16.1	15.7	0.4	26
C5	16.2	14.3	1.9	27
C18	65.8	65.9	-0.1	28
C1	23.0	21.9	1.1	29
C7	29.2	22.2	7.0	30
RMSD		2.5		
Max Abs		6.9		

mol2 coordinates for lowest energy conformer

1	H1	-2.761622196	-0.993654566	2.071138304

2	H2	1.508137234	0.968017589	-1.101297472
3	H3	-1.456934121	-1.080431701	0.246601168
4	H4	2.825643876	-1.740696215	-1.132091581
5	C5	2.396015752	-2.150337086	-3.460885136
6	C6	4.820668527	-1.511844610	-4.707465699
7	C7	-4.379908599	-0.467464274	4.086312281
8	C8	-4.815493395	1.683258982	2.932229396
9	C9	-2.984266091	2.034510463	0.340667720
10	C10	0.896057229	-1.953153864	0.698814649
11	C11	0.143549669	1.940933407	0.859154513
12	O12	3.657051352	2.061510321	-1.917336911
13	O13	-6.685952489	-0.470363967	2.276056281
14	O14	4.617906659	0.701565426	-4.071568884
15	C15	5.473794984	-0.793306754	-2.424627817
16	C16	5.243132057	-0.501829341	-1.148874541
17	C17	3.896974690	1.523458197	-0.624536128
18	C18	-5.267556281	-0.109497560	0.340754651
19	C19	-3.854382333	-0.242644498	-0.225588885
20	C20	0.183554916	-0.486602540	-1.836075854
21	C21	-1.082013482	0.235850410	-1.368816538
22	C22	-1.810993977	0.679842101	2.940039688
23	C23	-0.450285086	0.155902835	2.479579243
24	C24	2.358418560	-0.102011960	1.413859268
25	C25	3.720852501	-0.481022790	0.820213846
26	C26	2.975665016	-0.798106905	-3.010061128
27	C27	-5.349839498	-0.532914910	1.801994339
28	C28	4.452734868	-0.606140507	-3.529509636
29	C29	-4.356212338	0.232067519	2.714139706
30	C30	-2.937566469	0.096144151	2.082626044
31	C31	1.410453328	-0.116961836	-0.991412779
32	C32	-1.370808194	0.006631890	0.121857798
33	C33	2.773187747	-0.695860200	-1.462422742
34	C34	-2.781508621	0.521217996	0.589559062
35	C35	1.128389748	-0.431605802	0.522286582
36	C36	-0.139502695	0.425845215	0.991002539
37	C37	3.906562942	-0.021294921	-0.641303876
38	H38	1.392409162	-2.307835931	-3.065117696
39	H39	2.333275809	-2.232585281	-4.548802232
40	H40	3.017705482	-2.975426147	-3.091826294
41	H41	4.139581816	-1.347725123	-5.546880219
42	H42	4.817290693	-2.572143481	-4.441299883
43	H43	5.827223639	-1.240124808	-5.039867604
44	H44	-3.882311726	-1.444417418	4.044124615
45	H45	-5.418004243	-0.628858898	4.391895148
46	H46	-3.889490040	0.129478378	4.862071688
47	H47	-5.719343398	1.700295322	3.551881565
48	H48	-4.051592855	2.256510710	3.467336532
49	H49	-5.033110523	2.216337499	2.003106800
50	H50	-4.024924823	2.262540162	0.094535721
51	H51	-2.724607829	2.653941229	1.202465721
52	H52	-2.384021358	2.380866697	-0.505008003
53	H53	1.785112112	-2.513009376	0.396414789
54	H54	0.064062751	-2.351681023	0.116988813
55	H55	0.713950646	-2.209534663	1.745346399
56	H56	-0.675097072	2.526639643	1.273787675
57	H57	1.034857628	2.234140025	1.419097927

58	H58	0.279942106	2.275221948	-0.170419269
59	H59	3.711588228	3.022380228	-1.865464040
60	H60	-7.009724671	0.423935048	2.108195290
61	H61	4.399064467	1.320096505	-3.353897142
62	H62	6.472076688	-1.095241813	-2.737190632
63	H63	6.049495780	-0.570376345	-0.417653165
64	H64	4.868609504	1.865190394	-0.238672510
65	H65	3.122530062	1.896950976	0.053079032
66	H66	-5.961160148	-0.727576514	-0.239551634
67	H67	-5.625820694	0.924949525	0.241337717
68	H68	-3.859475520	0.103366479	-1.264847865
69	H69	-3.589004998	-1.309320768	-0.252163571
70	H70	-0.000863143	-1.565930957	-1.810654246
71	H71	0.363003065	-0.230162318	-2.884911954
72	H72	-1.920626257	-0.119209179	-1.975037225
73	H73	-0.982137572	1.304747135	-1.589684516
74	H74	-1.946981554	0.394275784	3.987526586
75	H75	-1.832985750	1.775795893	2.924667039
76	H76	0.339557738	0.589891963	3.104632643
77	H77	-0.442245404	-0.921802187	2.673779753
78	H78	2.380097199	0.959172376	1.667132268
79	H79	2.247888111	-0.619173771	2.374082214
80	H80	3.867414509	-1.567380095	0.850394965
81	H81	4.513085632	-0.053421385	1.450974516
82	H82	2.418794965	0.005214503	-3.505084176
83	H83	-5.088994000	-1.598010635	1.864166988

---

2 lowest energy conformers were considered for Boltzmann average to contribute to the ^{13}C NMR data

III.11. Cartesian coordinates of the global minimum conformer of 20β,28-epoxytaraxast-21-en-3β-ol (4R)

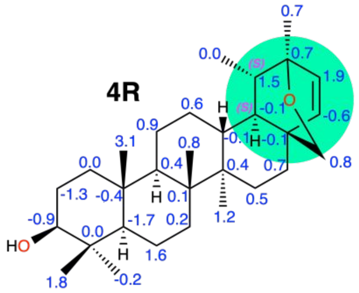

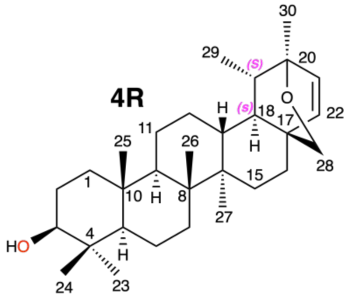

no	δ_{calc}	δ_{exp}	diff	IUPAC no	
C12	38.8	38.8	0.0	1	
C11	25.3	26.6	-1.3	2	*
C23	78.1	79.0	-0.9	3	
C25	38.9	38.9	0.0	4	
C21	53.7	55.4	-1.7	5	
C10	19.8	18.2	1.6	6	
C13	34.1	33.9	0.2	7	
C28	40.8	40.7	0.1	8	
C22	51.0	50.6	0.4	9	
C26	36.8	37.2	-0.4	10	
C9	22.2	21.3	0.9	11	

C8	27.9	27.3	0.6	12	
C20	38.6	38.7	-0.1	13	
C27	42.0	41.6	0.4	14	
C14	27.9	27.4	0.5	15	*
C16	28.2	27.5	0.7	16	
C30	37.5	37.6	-0.1	17	
C24	45.3	45.4	-0.1	18	
C19	45.7	44.2	1.5	19	
C29	74.9	74.2	0.7	20	
C15	134.9	133.0	1.9	21	
C17	140.0	140.6	-0.6	22	
C2	27.8	28.0	-0.2	23	
C3	17.5	15.7	1.8	24	*
C4	19.4	16.3	3.1	25	
C6	16.2	15.4	0.8	26	*
C5	15.5	14.3	1.2	27	
C18	66.7	65.9	0.8	28	
C1	20.9	20.9	0.0	29	
C7	22.9	22.2	0.7	30	
RMSD		1.1			
Max Abs		3.1			

mol2 coordinates for lowest energy conformer

1	H1	-1.152226219	2.190317199	2.395472574
2	H2	-1.151213166	1.213304335	0.389727423
3	H3	1.036428366	-1.382721977	-1.459426047
4	H4	-1.733576360	-2.500391237	-1.982214441
5	C5	-1.483247640	-2.158477564	-4.442904254
6	C6	-0.736207569	3.468785649	4.657314690
7	C7	1.380170820	4.244079865	3.626235085
8	C8	0.658135192	-4.124474543	-5.391746802
9	C9	1.860412798	2.892661850	0.764790863
10	C10	1.970194573	-0.283902857	0.756320715
11	C11	-1.841851072	-1.293463099	0.501416623
12	O12	-0.781933880	6.094031210	3.175252667
13	C13	-0.721469625	-5.025098995	-3.466392907
14	C14	-0.954951780	-4.984892731	-2.156384287
15	C15	-0.417570478	5.007787349	1.119478195
16	C16	-0.459877739	3.704255018	0.323625147
17	C17	0.163189910	1.204663537	-1.261166868
18	C18	0.577962181	1.215378173	3.110178023
19	C19	-0.499839920	0.001898153	-1.933757680
20	C20	0.136692575	-0.075539110	2.418721252
21	C21	0.184072515	-2.699672834	0.783062014
22	C22	-0.420763344	-3.918593732	0.081792546
23	C23	1.285494253	-4.135296511	-1.786315105
24	O24	1.484426310	-4.124252120	-3.185882435
25	C25	-0.856241791	4.816991428	2.561049153
26	C26	-0.291564027	-2.569916056	-3.578512096
27	C27	-0.034221830	3.734502115	3.311153684
28	C28	0.262387785	-3.975481341	-3.934477430
29	C29	-0.076171756	2.433702629	2.451512870
30	C30	-0.061859095	1.214967646	0.256116712
31	C31	-0.051070730	-1.326655181	-1.318841971
32	C32	0.363461883	2.557004114	0.958797449
33	C33	0.441408710	-0.119793839	0.905819925

34	C34	-0.320543067	-1.348513853	0.220140483
35	C35	-0.640302484	-2.543287542	-2.061102488
36	C36	-0.200258311	-3.898974525	-1.429745857
37	H37	-2.286000400	-2.901865784	-4.369868276
38	H38	-1.207515497	-2.052454183	-5.496978964
39	H39	-1.891434906	-1.199881027	-4.107137060
40	H40	-1.722301503	3.009771058	4.514560434
41	H41	-0.872220367	4.402612977	5.215742108
42	H42	-0.147473944	2.813755247	5.305810165
43	H43	1.989767561	3.446496712	4.064878772
44	H44	1.326583404	5.065250794	4.346064321
45	H45	1.904066529	4.623012811	2.748123937
46	H46	1.328606210	-3.313960431	-5.693320031
47	H47	-0.222924790	-4.113386931	-6.040248706
48	H48	1.180634003	-5.075404632	-5.530645827
49	H49	2.020496900	3.971855794	0.703756788
50	H50	2.495287973	2.529833674	1.575594054
51	H51	2.241750891	2.468475134	-0.167741631
52	H52	2.309008152	-0.303148033	-0.281878787
53	H53	2.503802769	0.529531181	1.244742358
54	H54	2.314674533	-1.204815655	1.232988818
55	H55	-2.384917643	-2.023449001	-0.101768139
56	H56	-2.293383481	-0.322773204	0.290105798
57	H57	-2.056832935	-1.534318692	1.546807572
58	H58	-1.260564553	6.057354172	4.010174001
59	H59	-1.159933882	-5.743549473	-4.153295248
60	H60	-1.607468352	-5.672429678	-1.623130134
61	H61	-1.078216992	5.746167752	0.653220899
62	H62	0.584818867	5.450393686	1.111455362
63	H63	-0.106102701	3.907283401	-0.692877782
64	H64	-1.507738207	3.382919762	0.231512792
65	H65	-0.239090914	2.116913021	-1.711277357
66	H66	1.233921822	1.193737460	-1.499238114
67	H67	0.283322017	1.143483962	4.161168989
68	H68	1.669922505	1.307716325	3.111817683
69	H69	-1.592789563	0.093938758	-1.866252952
70	H70	-0.256556968	0.022908901	-3.001356626
71	H71	0.606117813	-0.931491158	2.918088646
72	H72	-0.941928918	-0.169550201	2.584474036
73	H73	1.271361216	-2.765257565	0.692169894
74	H74	-0.038175118	-2.763292900	1.854309030
75	H75	-1.496917415	-3.982632099	0.283327028
76	H76	0.020625373	-4.832401984	0.501378328
77	H77	1.929821932	-3.363987036	-1.345900555
78	H78	1.600791972	-5.107708411	-1.380783627
79	H79	-1.906463610	4.472312367	2.549530320
80	H80	0.544094232	-1.882897489	-3.767209743

---

3 lowest energy conformers were considered for Boltzmann average to contribute to the ^{13}C NMR data

III.12. Cartesian coordinates of the global minimum conformer of oleandenic acid (5P)

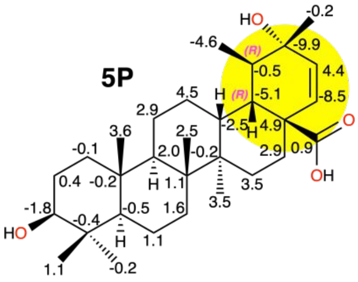

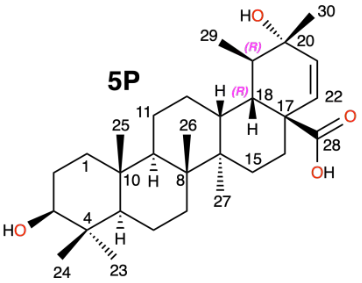

no	δ_{calc}	δ_{exp}	diff	IUPAC no
C1	39.0	39.1	-0.1	1
C2	28.0	27.6	0.4	2
C3	77.4	79.2	-1.8	3
C4	38.7	39.1	-0.4	4
C5	55.1	55.6	-0.5	5
C6	19.6	18.5	1.1	6
C7	35.8	34.2	1.6	7
C8	41.9	40.8	1.1	8
C9	52.7	50.7	2.0	9
C10	37.2	37.4	-0.2	10
C11	24.2	21.3	2.9	11
C12	32.1	27.6	4.5	12
C13	40.0	42.5	-2.5	13
C14	41.3	41.5	-0.2	14
C15	30.6	27.1	3.5	15
C16	28.6	25.7	2.9	16
C17	53.3	48.4	4.9	17
C18	42.4	47.5	-5.1	18
C19	44.3	44.8	-0.5	19
C20	74.1	84.0	-9.9	20
C21	138.4	134.0	4.4	21
C22	130.2	138.7	-8.5	22
C23	28.0	28.2	-0.2	23
C24	16.7	15.6	1.1	24
C25	19.5	15.9	3.6	25
C26	19.0	16.5	2.5	26
C27	17.8	14.3	3.5	27
C28	176.7	175.8	0.9	28
C29	15.3	19.9	-4.6	29
C30	21.1	21.3	-0.2	30
RMSD		3.5		
Max Abs		9.9		

mol2 coordinates for lowest energy conformer

1	C1	-0.115209335	-3.769267391	0.150672001
2	C2	-0.158500437	-5.092370737	0.916519862
3	C3	0.480190324	-4.989932963	2.296627243
4	C4	-0.131049408	-3.865071933	3.171087854
5	C5	-0.093095764	-2.551050112	2.335441783
6	C6	-0.511885080	-1.295263526	3.105137509
7	C7	0.000481348	-0.050418116	2.382723942
8	C8	-0.548916854	0.096571314	0.948192551
9	C9	-0.326106542	-1.255895194	0.187336353
10	C10	-0.749546620	-2.583969139	0.921823954
11	C11	-0.840631434	-1.145278612	-1.253266747
12	C12	-0.073144679	-0.060740549	-2.004602086

13	C13	-0.230086893	1.297606828	-1.315732192
14	C14	0.225759929	1.270768379	0.175589102
15	C15	-0.135156085	2.637998102	0.804550426
16	C16	0.337981558	3.840655609	-0.007777895
17	C17	-0.217817051	3.823877013	-1.448938731
18	C18	0.210765569	2.523674249	-2.180867177
19	C19	1.660802608	2.683521785	-2.712976182
20	C20	1.727279705	3.833935345	-3.758772319
21	C21	1.072032372	5.072618439	-3.204398640
22	C22	0.235724238	5.076496286	-2.172749090
23	C23	0.775975910	-3.717838567	4.407322331
24	C24	-1.529806868	-4.256617022	3.676522758
25	C25	-2.280116945	-2.800277497	0.940060751
26	C26	-2.054542698	0.435155133	1.069636501
27	C27	1.755222556	1.095727090	0.281308179
28	C28	-1.747404208	3.928988315	-1.445431458
29	C29	2.326611821	1.429167156	-3.280365959
30	C30	1.126785802	3.478318321	-5.118786394
31	H31	-1.314751977	1.444735829	-1.281038182
32	O32	3.095198250	4.141621831	-4.041116967
33	O33	0.484688607	-6.249300690	2.950751126
34	O34	-2.196420399	4.837899230	-0.554161899
35	O35	-2.507183181	3.334829559	-2.172606669
36	H36	0.763938461	-1.364971816	0.104027405
37	H37	-0.427041261	2.444686214	-3.069271344
38	H38	0.982131401	-2.413903054	2.124995628
39	H39	-1.195271948	-5.442204110	1.021603160
40	H40	0.366113151	-5.868539690	0.348940677
41	H41	1.543349362	-4.750153424	2.162125734
42	H42	-0.612914565	-3.907777838	-0.815204247
43	H43	0.934719920	-3.529517309	-0.070642358
44	H44	-0.086376440	-1.307659170	4.113046496
45	H45	-1.599921674	-1.251981331	3.235343195
46	H46	-0.239504311	0.846012005	2.966985709
47	H47	1.092760323	-0.127140815	2.361229984
48	H48	-0.440561109	0.014039685	-3.035741459
49	H49	0.973303337	-0.368638366	-2.067084369
50	H50	-0.720036801	-2.095831953	-1.781844402
51	H51	-1.912679161	-0.913777312	-1.271448961
52	H52	0.279589173	2.706324348	1.816962349
53	H53	-1.220131229	2.729051232	0.911069737
54	H54	1.431082520	3.868188098	-0.068972269
55	H55	0.034413688	4.764608576	0.493036519
56	H56	-0.167563458	6.014935818	-1.797170016
57	H57	1.349620517	6.004700024	-3.693544579
58	H58	2.297368529	3.040216791	-1.890620408
59	H59	3.496593860	4.443104196	-3.215877688
60	H60	0.077523897	3.185875787	-5.028515866
61	H61	1.184556409	4.348052372	-5.779733152
62	H62	1.688921732	2.663682327	-5.583712997
63	H63	1.667749434	0.887130973	-3.965037217
64	H64	3.225313883	1.723405849	-3.829823259
65	H65	2.630290643	0.742622831	-2.487476918
66	H66	-2.637216568	-0.435085513	1.365201665
67	H67	-2.221088018	1.194677976	1.839854304
68	H68	-2.495747855	0.807624037	0.141437975

69	H69	-2.533943038	-3.863001195	0.912027454
70	H70	-2.765163487	-2.389462295	1.828900910
71	H71	-2.752235729	-2.348481725	0.063450840
72	H72	-2.015469153	-3.408691028	4.171198197
73	H73	-2.200756466	-4.601100267	2.884978876
74	H74	-1.447257812	-5.056428469	4.421469974
75	H75	0.308595762	-3.113940203	5.191340086
76	H76	1.734520242	-3.252925115	4.146169707
77	H77	0.981157313	-4.708111214	4.824013188
78	H78	-0.427623571	-6.562684178	2.977195471
79	H79	-3.159864694	4.866548345	-0.668744924
80	H80	2.274844453	1.799789868	-0.368527786
81	H81	2.094536921	0.096157826	-0.002539396
82	H82	2.111245278	1.289698445	1.297212739

---

13 lowest energy conformers were considered for Boltzmann average to contribute to the ^{13}C NMR data

III.13. Cartesian coordinates of the global minimum conformer of 3β-hydroxyurs-21-en-28(20β)-olide (6P)

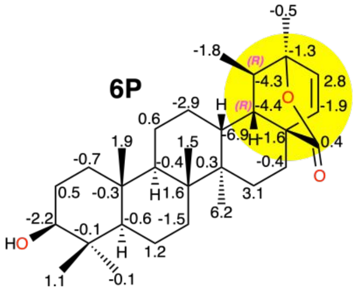

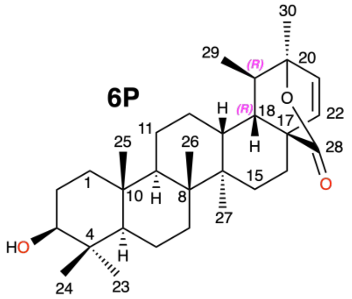

no	δ_{calc}	δ_{exp}	diff	IUPAC no
C12	38.2	38.9	-0.7	1
C11	27.9	27.4	0.5	2
C23	77.3	79.5	-2.2	3
C25	38.7	38.8	-0.1	4
C21	54.8	55.4	-0.6	5
C10	19.4	18.2	1.2	6
C13	32.5	34.0	-1.5	7
C28	42.2	40.6	1.6	8
C22	50.0	50.4	-0.4	9
C26	36.8	37.1	-0.3	10
C9	21.7	21.1	0.6	11
C8	24.4	27.3	-2.9	12
C20	35.3	42.2	-6.9	13
C27	41.5	41.2	0.3	14
C14	29.9	26.8	3.1	15
C16	25.0	25.4	-0.4	16
C30	46.5	48.1	-1.6	17
C24	42.9	47.3	-4.4	18
C19	40.3	44.6	-4.3	19
C29	82.4	83.7	-1.3	20
C31	136.5	133.7	2.8	21
C32	136.5	138.4	-1.9	22

C2	27.9	28.0	-0.1	23
C3	16.4	15.3	1.1	24
C4	18.1	16.2	1.9	25
C6	17.2	15.7	1.5	26
C5	20.3	14.1	6.2	27
C15	175.9	175.5	0.4	28
C1	17.9	19.7	-1.8	29
C7	20.5	21.0	-0.5	30
RMSD		2.5		
Max Abs		6.9		

mol2 coordinates for lowest energy conformer

1	H1	1.614696579	-2.063523677	1.888901247
2	H2	0.937310495	-1.140305953	-0.002277353
3	H3	-1.879444737	1.262100877	-0.985031342
4	H4	-1.836017090	2.661646160	-2.572760961
5	C5	-0.770203923	2.066764695	-4.741265308
6	C6	2.012894074	-3.254361166	4.202439744
7	C7	-0.320103043	-4.059532994	3.985169479
8	C8	1.033756664	4.366988017	-5.150908453
9	C9	-1.755966086	-2.822248859	1.384693366
10	C10	-1.968280464	0.285423766	1.282923066
11	C11	1.495814909	1.362998011	-0.291319381
12	O12	1.715822244	-5.884302022	2.938237416
13	C13	1.471217975	4.393671556	-2.669785149
14	C14	0.936682817	4.299239867	-1.458561141
15	C15	0.544365350	-4.926590893	1.040575769
16	C16	0.294422607	-3.657812563	0.225390213
17	C17	-0.831539798	-1.230597709	-1.159719663
18	C18	0.211453391	-1.065909006	3.111229649
19	C19	-0.553764924	-0.002894151	-2.031050928
20	C20	0.339609018	0.202376675	2.267559147
21	C21	-0.316190980	2.754345804	0.760747480
22	C22	-1.107814406	3.789875362	-0.050538435
23	C23	-1.201886545	4.925596912	-2.351456693
24	O24	-0.677627226	4.912512860	-3.599783891
25	C25	1.437502134	-4.675469780	2.249905531
26	C26	0.037027326	2.578305746	-3.545684242
27	C27	0.897580393	-3.564609780	3.186725332
28	C28	0.497473393	4.057455338	-3.767656864
29	C29	0.623482311	-2.304313050	2.311439236
30	C30	-0.138991295	-1.171047521	0.208419526
31	C31	-0.830944220	1.313231579	-1.299519320
32	C32	-0.285873174	-2.487729460	1.055855267
33	C33	-0.459342448	0.170075380	0.947136977
34	C34	-0.017112527	1.399702109	0.025808202
35	C35	-0.794937594	2.567891719	-2.236106583
36	C36	-0.526206958	3.903736967	-1.446477037
37	H37	-0.147834003	1.966518864	-5.635428394
38	H38	-1.588003563	2.759383709	-4.968133039
39	H39	-1.208779637	1.088089885	-4.539726105
40	H40	2.845840223	-2.720231484	3.729792815
41	H41	2.399558616	-4.191958159	4.610572876
42	H42	1.651897921	-2.647981103	5.038628560
43	H43	-0.782322020	-3.237705414	4.539620633
44	H44	-0.004278004	-4.804338772	4.723603506

45	H45	-1.097670689	-4.511138493	3.364510400
46	H46	0.261541410	4.242298974	-5.912598323
47	H47	1.871511446	3.701115919	-5.384246020
48	H48	1.388236090	5.401027052	-5.188169543
49	H49	-1.894984271	-3.892691827	1.550237990
50	H50	-2.128162136	-2.311969460	2.274380803
51	H51	-2.413552153	-2.553994840	0.553777166
52	H52	-2.612541645	-0.189965922	0.541060745
53	H53	-2.198036542	-0.178874026	2.243836749
54	H54	-2.292744251	1.326089486	1.358720853
55	H55	1.740161483	2.109923882	-1.048958506
56	H56	1.842373179	0.401048811	-0.673441377
57	H57	2.098195766	1.600358591	0.590429905
58	H58	0.868848051	-6.286103588	3.167989232
59	H59	1.019503826	-5.686684397	0.411059917
60	H60	-0.408569856	-5.360332120	1.375025737
61	H61	-0.375635142	-3.902541125	-0.606161140
62	H62	1.246414868	-3.337982772	-0.222924390
63	H63	-0.505732358	-2.119759084	-1.707721904
64	H64	-1.916968118	-1.331457856	-1.043095017
65	H65	0.854820843	-0.953748751	3.989035775
66	H66	-0.807753518	-1.174526018	3.500171700
67	H67	0.481644042	-0.027939070	-2.391491978
68	H68	-1.188903016	-0.071139518	-2.919560316
69	H69	0.039870859	1.068973472	2.868664700
70	H70	1.404705727	0.332949456	2.045694557
71	H71	-0.852187589	2.587142060	1.697395987
72	H72	0.636154202	3.201552475	1.066167134
73	H73	-1.083858094	4.761150624	0.454712970
74	H74	-2.164601029	3.508299698	-0.124796124
75	H75	2.417271267	-4.339057248	1.884732964
76	H76	0.946910591	1.977151853	-3.436138986
77	H77	2.496430153	4.674382497	-2.887949378
78	H78	1.461390177	4.524542744	-0.536161502
79	O79	-2.129027349	5.637430184	-2.066375539

---

6 lowest energy conformers were considered for Boltzmann average to contribute to the ^{13}C NMR data

III.14. The four stereoisomers of ursane 6P and the revised structure 5R

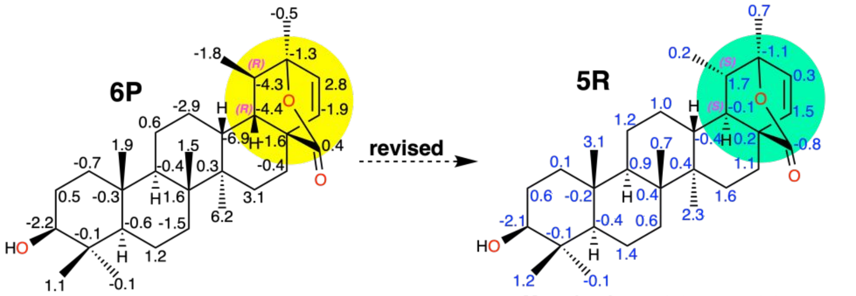

Max absolute = 6.9; rms = 2.5
3β-hydroxyurs-21-en-28→20β-olide

Max absolute = 3.1; rms = 1.1
3β-hydroxytaraxast-21-en-28→20β-olide

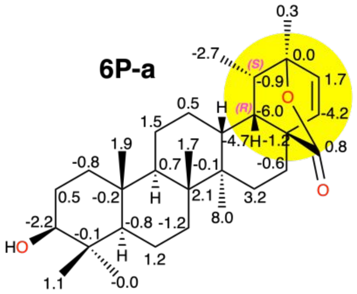

Max absolute = 8.0; rms = 2.5

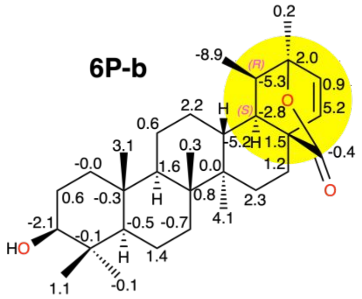

Max absolute = 8.9; rms = 2.8

III.15. Cartesian coordinates of the global minimum conformer of 6P-a

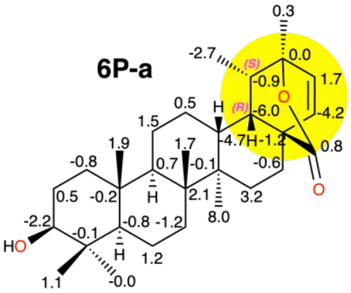

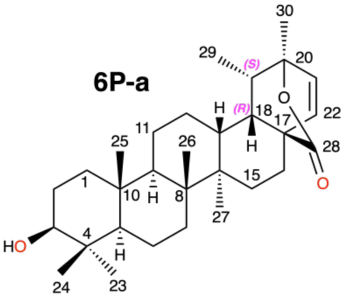

no	δ_{calc}	δ_{exp}	diff	IUPAC no
C12	38.1	38.9	-0.8	1
C11	27.9	27.4	0.5	2
C23	77.3	79.5	-2.2	3
C25	38.7	38.8	-0.1	4
C21	54.6	55.4	-0.8	5
C10	19.4	18.2	1.2	6
C13	32.8	34.0	-1.2	7
C28	42.7	40.6	2.1	8
C22	51.1	50.4	0.7	9
C26	36.9	37.1	-0.2	10
C9	22.6	21.1	1.5	11
C8	27.8	27.3	0.5	12
C20	37.5	42.2	-4.7	13
C27	41.1	41.2	-0.1	14
C14	30.0	26.8	3.2	15
C16	24.8	25.4	-0.6	16
C30	46.9	48.1	-1.2	17
C24	41.3	47.3	-6.0	18
C19	43.7	44.6	-0.9	19
C29	83.7	83.7	0.0	20
C31	135.4	133.7	1.7	21

C32	134.2	138.4	-4.2	22
C2	28.0	28.0	0.0	23
C3	16.4	15.3	1.1	24
C4	18.1	16.2	1.9	25
C6	17.4	15.7	1.7	26
C5	22.1	14.1	8.0	27
C15	176.3	175.5	0.8	28
C1	17.0	19.7	-2.7	29
C7	21.3	21	0.3	30
RMSD		2.5		
Max Abs		8.0		

mol2 coordinates for lowest energy conformer

1	H1	1.424366666	-2.071597075	1.968984816
2	H2	0.912598022	-1.135067808	0.010100769
3	H3	-1.798353095	1.184542183	-1.197985091
4	H4	-1.702774664	2.524553765	-2.773149189
5	C5	1.553401705	1.959437477	-3.680600999
6	C6	1.631207391	-3.293591667	4.287504239
7	C7	-0.656747377	-4.138056390	3.844950849
8	C8	0.962344671	4.678495382	-5.127591146
9	C9	-1.863455803	-2.890130160	1.128869855
10	C10	-2.117802398	0.226845243	1.052234010
11	C11	1.464296948	1.430370891	-0.124069087
12	O12	1.498125719	-5.912216641	2.971507985
13	C13	1.303307992	4.691708756	-2.607520709
14	C14	0.727282241	4.473073322	-1.429675625
15	C15	0.498689894	-4.945591858	0.982275410
16	C16	0.306715060	-3.669066924	0.164107296
17	C17	-0.752962330	-1.247218604	-1.290464221
18	C18	-0.116456443	-1.126630879	3.061411328
19	C19	-0.347098676	-0.024395987	-2.109667185
20	C20	0.079677034	0.155628372	2.253741972
21	C21	-0.497009844	2.727557816	0.703465490
22	C22	-1.325873851	3.683211827	-0.158933350
23	C23	-1.431296280	4.820441418	-2.433168887
24	O24	-0.851128650	4.906719253	-3.649363846
25	C25	1.266216612	-4.698364830	2.274963428
26	C26	0.219226916	2.709659322	-3.605712415
27	C27	0.619890082	-3.609474362	3.169960019
28	C28	0.447330536	4.249348189	-3.767084242
29	C29	0.402926371	-2.341970447	2.289508067
30	C30	-0.178074212	-1.191631228	0.133440890
31	C31	-0.727656139	1.282687642	-1.409596307
32	C32	-0.375419374	-2.522406227	0.949122256
33	C33	-0.582449153	0.136350535	0.858033285
34	C34	-0.072197098	1.390221470	0.002285900
35	C35	-0.694829145	2.540944025	-2.343202688
36	C36	-0.665313731	3.883246847	-1.511143696
37	H37	2.128369467	2.034766555	-2.757393252
38	H38	2.172332340	2.355331822	-4.491346877
39	H39	1.395725534	0.904200526	-3.900245430
40	H40	2.491692772	-2.735024834	3.900288066
41	H41	1.999121895	-4.230201295	4.715149104
42	H42	1.183559760	-2.708412283	5.096717752
43	H43	-1.180314280	-3.333909837	4.369909160

44	H44	-0.397177614	-4.892525078	4.595226664
45	H45	-1.366697151	-4.587431427	3.146886329
46	H46	0.319775201	4.288215437	-5.921388434
47	H47	1.983381390	4.326413728	-5.294372717
48	H48	0.963186745	5.769938011	-5.187863149
49	H49	-1.995492932	-3.965818044	1.267863644
50	H50	-2.330664090	-2.399701636	1.984815908
51	H51	-2.443816957	-2.623453691	0.241056584
52	H52	-2.684994807	-0.216526284	0.231980147
53	H53	-2.431454104	-0.286604009	1.962747101
54	H54	-2.458496081	1.260201219	1.148417671
55	H55	1.754010978	2.284642650	-0.741580674
56	H56	1.898512866	0.536675239	-0.578487265
57	H57	1.945638639	1.573739201	0.847275368
58	H58	0.640600759	-6.332815686	3.109222764
59	H59	1.045527452	-5.688328742	0.391367615
60	H60	-0.474007570	-5.400070003	1.217204918
61	H61	-0.273199800	-3.914210747	-0.732521348
62	H62	1.291982007	-3.325489116	-0.183714329
63	H63	-0.394966864	-2.143267288	-1.806102656
64	H64	-1.846537621	-1.322264304	-1.275373886
65	H65	0.426334217	-1.017069424	4.005335466
66	H66	-1.169200208	-1.259966296	3.336500242
67	H67	0.730665730	-0.069618587	-2.282212135
68	H68	-0.823425755	-0.065054942	-3.097901473
69	H69	-0.288977101	1.010316086	2.833119494
70	H70	1.160384110	0.297111292	2.146894998
71	H71	-1.050663236	2.536873547	1.625302126
72	H72	0.407958782	3.251466332	1.030798472
73	H73	-1.436893338	4.647397182	0.348666678
74	H74	-2.342129658	3.301428395	-0.310201934
75	H75	2.270700741	-4.341537123	2.010266374
76	H76	-0.373899813	2.400118337	-4.474792096
77	H77	2.289039971	5.120319974	-2.755849913
78	H78	1.167476959	4.734829672	-0.472692447
79	O79	-2.463674931	5.387682822	-2.186511807

---

3 lowest energy conformers were considered for Boltzmann average to contribute to the ^{13}C NMR data

III.16. Cartesian coordinates of the global minimum conformer of 6P-b

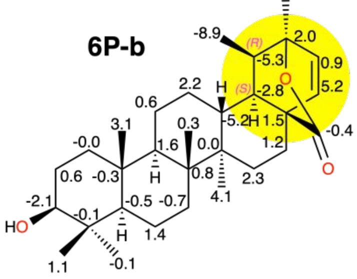

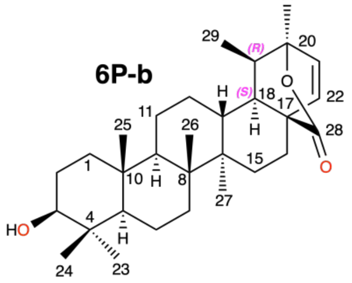

no	δ_{calc}	δ_{exp}	diff	IUPAC no
C12	38.9	38.9	0.0	1
C11	28.0	27.4	0.6	2
C23	77.4	79.5	-2.1	3

C25	38.7	38.8	-0.1	4
C21	54.9	55.4	-0.5	5
C10	19.6	18.2	1.4	6
C13	33.3	34.0	-0.7	7
C28	41.4	40.6	0.8	8
C22	52.0	50.4	1.6	9
C26	36.8	37.1	-0.3	10
C9	21.7	21.1	0.6	11
C8	29.5	27.3	2.2	12
C20	37.0	42.2	-5.2	13
C27	41.2	41.2	0.0	14
C14	29.1	26.8	2.3	15
C16	26.6	25.4	1.2	16
C30	46.6	48.1	-1.5	17
C24	44.5	47.3	-2.8	18
C19	39.3	44.6	-5.3	19
C29	81.7	83.7	-2.0	20
C31	134.6	133.7	0.9	21
C32	143.6	138.4	5.2	22
C2	27.9	28.0	-0.1	23
C3	16.4	15.3	1.1	24
C4	19.3	16.2	3.1	25
C6	16.0	15.7	0.3	26
C5	18.2	14.1	4.1	27
C15	175.1	175.5	-0.4	28
C1	10.8	19.7	-8.9	29
C7	21.2	21.0	0.2	30
RMSD		2.8		
Max Abs		8.9		

mol2 coordinates for lowest energy conformer

1	H1	-2.061034381	-1.268024030	2.434085141
2	H2	-1.083894964	-1.317519589	0.452469520
3	H3	1.276816706	0.965697812	-1.537451035
4	H4	2.569845959	-1.700032638	-2.118454432
5	C5	1.823412177	1.050023944	-4.032131130
6	C6	-3.344230283	-0.797100960	4.691381376
7	C7	-4.230446983	1.217082749	3.548255474
8	C8	4.450774806	0.400094549	-5.440861117
9	C9	-2.937289558	1.604180359	0.642201057
10	C10	0.197847135	1.918519998	0.605551116
11	C11	1.477383003	-1.817458491	0.544780703
12	O12	-5.917384881	-1.165918254	3.333495682
13	C13	5.026504884	-1.032413663	-3.451816243
14	C14	4.972256934	-1.159656852	-2.131045471
15	C15	-4.915048817	-0.770973071	1.159353843
16	C16	-3.618983040	-0.784806924	0.349252306
17	C17	-1.173850800	-0.149478387	-1.297259035
18	C18	-1.172319240	0.544718179	3.050203527
19	C19	0.079597956	-0.775664533	-1.907323513
20	C20	0.138519249	0.158224420	2.363050697
21	C21	2.738097438	0.318111175	0.680013119
22	C22	3.971671510	-0.337136742	0.062141129
23	C23	4.364193493	1.179317452	-1.908680597
24	O24	4.506121125	1.293947066	-3.241527380
25	C25	-4.687773812	-1.109845693	2.627430889

26	C26	2.660776415	-0.178796435	-3.642057857
27	C27	-3.646767259	-0.185750533	3.310384832
28	C28	4.162390282	0.088706517	-3.983778981
29	C29	-2.357743617	-0.204294345	2.435501686
30	C30	-1.157808922	-0.244311291	0.235450110
31	C31	1.352595376	-0.114055880	-1.366770762
32	C32	-2.518456376	0.142693304	0.922165749
33	C33	0.143561094	0.392665829	0.836577201
34	C34	1.420541910	-0.311341673	0.179528511
35	C35	2.595931984	-0.604861727	-2.131140620
36	C36	3.977032217	-0.234435168	-1.459549997
37	H37	1.886336562	1.222071162	-5.110576018
38	H38	2.171507338	1.956994738	-3.530864547
39	H39	0.766305383	0.919329396	-3.796900772
40	H40	-2.753284339	-1.717099762	4.598696238
41	H41	-4.285503888	-1.047050600	5.190295861
42	H42	-2.794695634	-0.104532008	5.337074580
43	H43	-3.461668086	1.899908634	3.924630544
44	H44	-5.015172459	1.169614854	4.311944026
45	H45	-4.658816652	1.669450514	2.650149528
46	H46	3.946490438	1.313086183	-5.763401654
47	H47	4.120214976	-0.431882709	-6.071666243
48	H48	5.526706358	0.539809841	-5.583586108
49	H49	-4.024887288	1.704241465	0.584085069
50	H50	-2.599221954	2.305687891	1.407993080
51	H51	-2.541349711	1.949926371	-0.317040048
52	H52	0.081319331	2.207963661	-0.441883885
53	H53	-0.587213517	2.430274229	1.159841584
54	H54	1.144119575	2.334965000	0.957924610
55	H55	2.102446062	-2.377647821	-0.155309050
56	H56	0.504334048	-2.309800235	0.548513127
57	H57	1.915036237	-1.959073746	1.537559831
58	H58	-6.368411621	-0.324182599	3.194457678
59	H59	-5.623975539	-1.494263967	0.741687084
60	H60	-5.402462951	0.211467423	1.084305660
61	H61	-3.850972652	-0.505904539	-0.684434583
62	H62	-3.237292861	-1.815733594	0.318236455
63	H63	-2.053194020	-0.662431890	-1.697325191
64	H64	-1.247426621	0.893472707	-1.630955763
65	H65	-1.082068936	0.298437896	4.112556606
66	H66	-1.329080085	1.628847690	3.007724180
67	H67	0.105436352	-1.849286841	-1.678129829
68	H68	0.026930779	-0.712008405	-2.998398290
69	H69	0.963821587	0.711488552	2.825966565
70	H70	0.314993481	-0.902855227	2.574536936
71	H71	2.783547596	1.381081603	0.432787922
72	H72	2.804473421	0.237529772	1.770545923
73	H73	4.035297840	-1.392814419	0.348924778
74	H74	4.871672498	0.152501826	0.447224742
75	H75	-4.295283093	-2.133880965	2.684504257
76	H76	2.329661043	-1.024473067	-4.255181031
77	H77	5.639562780	-1.631430872	-4.117383597
78	H78	5.538549718	-1.878385224	-1.545972199
79	O79	4.550379784	2.126480609	-1.186583555

---

6 lowest energy conformers were considered for Boltzmann average to contribute to the ^{13}C NMR data

III.17. Cartesian coordinates of the global minimum conformer of 3β-hydroxytaraxast-21-en-28(20β)-olide (5R)

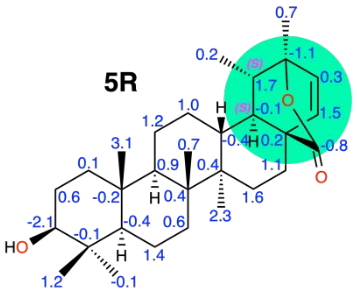

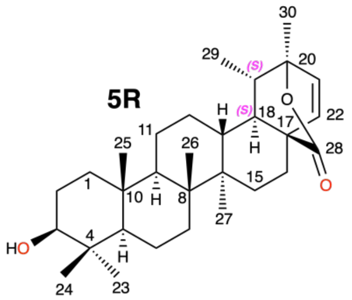

no	δ_{calc}	δ_{exp}	diff	IUPAC no
C12	39.0	38.9	0.1	1
C11	28.0	27.4	0.6	2
C23	77.4	79.5	-2.1	3
C25	38.7	38.8	-0.1	4
C21	55.0	55.4	-0.4	5
C10	19.6	18.2	1.4	6
C13	34.6	34.0	0.6	7
C28	41.0	40.6	0.4	8
C22	51.3	50.4	0.9	9
C26	36.9	37.1	-0.2	10
C9	22.3	21.1	1.2	11
C8	28.3	27.3	1.0	12
C20	41.8	42.2	-0.4	13
C27	41.6	41.2	0.4	14
C14	28.4	26.8	1.6	15
C16	26.5	25.4	1.1	16
C30	48.3	48.1	0.2	17
C24	47.2	47.3	-0.1	18
C19	46.3	44.6	1.7	19
C29	82.6	83.7	-1.1	20
C31	134.0	133.7	0.3	21
C32	139.9	138.4	1.5	22
C2	27.9	28.0	-0.1	23
C3	16.5	15.3	1.2	24
C4	19.3	16.2	3.1	25
C6	16.4	15.7	0.7	26
C5	16.4	14.1	2.3	27
C15	174.7	175.5	-0.8	28
C1	19.9	19.7	0.2	29
C7	21.7	21	0.7	30
RMSD		1.1		
Max Abs		3.1		

mol2 coordinates for lowest energy conformer

1	H1	-2.063245210	-1.126650225	2.447228899
2	H2	-1.070966558	-1.182641081	0.438287697
3	H3	1.392107037	1.038641992	-1.539747258
4	H4	2.689176674	-1.689168630	-1.849868594
5	C5	2.316745532	-1.670144653	-4.332973158
6	C6	-3.374787495	-0.651017951	4.686300101
7	C7	-4.279887675	1.337097322	3.513387668

8	C8	4.123338659	0.562804654	-5.444274453
9	C9	-2.963862089	1.716006384	0.625968304
10	C10	0.222014090	2.045653021	0.640038863
11	C11	1.493587354	-1.687421051	0.631451935
12	O12	-5.927678060	-1.072581548	3.301959487
13	C13	5.118106517	-0.634273721	-3.437047373
14	C14	5.105702470	-0.747648559	-2.112565111
15	C15	-4.903660903	-0.688581072	1.135918237
16	C16	-3.597506076	-0.686980992	0.341519474
17	C17	-1.139848914	0.036956371	-1.280507452
18	C18	-1.201272276	0.700523585	3.053785257
19	C19	0.096030645	-0.611532401	-1.900764016
20	C20	0.115639855	0.288830778	2.396052385
21	C21	2.736058674	0.469689795	0.734985975
22	C22	4.001482868	-0.098265271	0.090439797
23	C23	4.058741346	1.426755072	-1.935740682
24	O24	4.049498335	1.511728411	-3.278788209
25	C25	-4.689966237	-1.006328562	2.611039518
26	C26	2.637328630	-0.389389514	-3.564392335
27	C27	-3.671672018	-0.058673190	3.296043026
28	C28	3.999942501	0.230846965	-3.971243152
29	C29	-2.372994552	-0.066668237	2.435774313
30	C30	-1.145205802	-0.104434691	0.245809698
31	C31	1.392070760	-0.039077920	-1.322432219
32	C32	-2.519614469	0.264634495	0.918870658
33	C33	0.150331226	0.520369408	0.869610546
34	C34	1.433034479	-0.192720701	0.232129351
35	C35	2.641022698	-0.608529821	-2.026702978
36	C36	3.966694818	-0.017017769	-1.431626803
37	H37	3.110348350	-2.413257321	-4.194133796
38	H38	2.199856132	-1.484885006	-5.405490094
39	H39	1.384343107	-2.111426573	-3.968599819
40	H40	-2.762286232	-1.558100412	4.610653273
41	H41	-4.317049985	-0.916915293	5.174419639
42	H42	-2.850335118	0.059240816	5.333430871
43	H43	-3.521722634	2.039896803	3.872562014
44	H44	-5.059522861	1.289100397	4.281605732
45	H45	-4.721600570	1.765804253	2.610465186
46	H46	3.266067269	1.153137223	-5.780258651
47	H47	4.176457995	-0.352761334	-6.040173775
48	H48	5.034116067	1.142492651	-5.617866929
49	H49	-4.052278979	1.795591460	0.561445260
50	H50	-2.644409178	2.428147927	1.389716864
51	H51	-2.570629199	2.064269128	-0.332660395
52	H52	0.312874618	2.325558013	-0.411800519
53	H53	-0.663422424	2.547684564	1.024297759
54	H54	1.075067319	2.479602414	1.166522717
55	H55	2.195456174	-2.245056022	0.006692718
56	H56	0.533563106	-2.202674361	0.553568976
57	H57	1.843549767	-1.799770723	1.662359668
58	H58	-6.384418263	-0.234828589	3.155684426
59	H59	-5.593538469	-1.429896728	0.717952817
60	H60	-5.409242481	0.283120795	1.043260921
61	H61	-3.822109812	-0.423024686	-0.697660909
62	H62	-3.197921678	-1.711256289	0.325575261
63	H63	-2.031331790	-0.428742755	-1.710803393

64	H64	-1.170812387	1.092372253	-1.577776552
65	H65	-1.129652842	0.482326119	4.123522473
66	H66	-1.353556772	1.783242582	2.977603534
67	H67	0.066669320	-1.698184506	-1.737673471
68	H68	0.064013263	-0.461897308	-2.985532701
69	H69	0.944498268	0.824787732	2.872567646
70	H70	0.262191115	-0.774595697	2.615554640
71	H71	2.727513489	1.539505763	0.521395714
72	H72	2.816510501	0.357772983	1.822043227
73	H73	4.149855447	-1.146534420	0.377679603
74	H74	4.870475947	0.458865538	0.456376552
75	H75	-4.282826862	-2.023693840	2.685612551
76	H76	1.892440805	0.375950028	-3.817037852
77	H77	5.839745149	-1.100055119	-4.099977942
78	H78	5.826299179	-1.311307884	-1.527059295
79	O79	4.130269312	2.419604733	-1.255991343

---

lowest energy conformers were considered for Boltzmann average to contribute to the ^{13}C NMR data

III.18. Cartesian coordinates of the global minimum conformer and chemical shifts published by Giner et al.{Giner, 1993 #15} for kokoonol [27-OH] (7a)

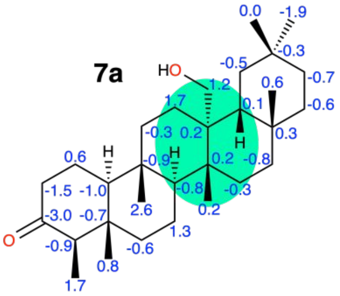

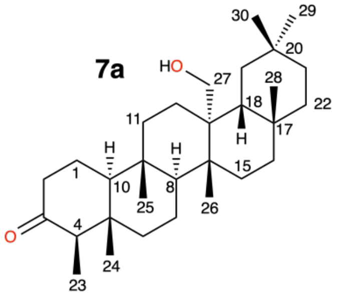

no	δ_{calc}	δ_{exp}	diff
C1	23.1	22.5	0.6
C2	40.1	41.6	-1.5
C3	210.3	213.3	-3.0
C4	57.5	58.4	-0.9
C5	41.6	42.3	-0.7
C6	40.9	41.5	-0.6
C7	19.9	18.6	1.3
C8	53.0	53.8	-0.8
C9	36.7	37.6	-0.9
C10	58.7	59.7	-1.0
C11	37.5	37.8	-0.3
C12	25.8	24.1	1.7
C13	45.6	45.4	0.2
C14	38.6	38.4	0.2
C15	31.9	32.2	-0.3
C16	35.5	36.3	-0.8
C17	30.6	30.3	0.3
C18	43.4	43.3	0.1
C19	36.6	37.1	-0.5
C20	28.2	28.5	-0.3
C21	31.9	32.6	-0.7
C22	39.5	40.1	-0.6

C23	8.8	7.1	1.7	
C24	15.7	14.9	0.8	
C25	20.8	18.2	2.6	
C26	22.5	22.3	0.2	
C27	64.6	63.4	1.2	
C28	32.2	32.8	0.6	*
C29	33.9	35.8	-1.9	
C30	30.6	30.6	0.0	*
RMSD		1.1		
Max Abs		2.9		

mol2 coordinates for lowest energy conformer

1	C1	-4.230251743	-0.042342085	1.259757866
2	C2	-5.183337442	-1.015885640	1.967314547
3	C3	-4.977188264	-1.002261681	3.466826059
4	C4	-3.533247112	-1.188122208	3.918201965
5	C5	-2.627937220	-0.088649464	3.258125043
6	C6	-1.163364482	-0.353787572	3.660537859
7	C7	-0.140907096	0.399410556	2.810618546
8	C8	-0.325949255	0.049445871	1.329487247
9	C9	-1.725152582	0.525884037	0.828852642
10	C10	-2.774566049	-0.244091699	1.706671495
11	C11	-1.883389700	0.067185354	-0.637007663
12	C12	-0.691922935	0.399862397	-1.540686957
13	C13	0.645122411	-0.180759201	-1.036888864
14	C14	0.897543534	0.375670673	0.416380614
15	C15	2.173608215	-0.293360891	1.006113770
16	C16	3.212823023	-0.798454591	-0.016812773
17	C17	3.268639887	-0.073429846	-1.380534601
18	C18	1.830652461	0.263878069	-1.958907419
19	C19	1.660606684	-0.275025810	-3.393634697
20	C20	2.800239769	0.044900018	-4.381023923
21	C21	4.166802274	-0.371436436	-3.759272328
22	C22	4.017756111	-0.992241358	-2.370325781
23	C23	-3.450166748	-1.302700115	5.438236809
24	C24	-3.056536623	1.292505214	3.784909848
25	C25	-1.947931357	2.057177250	0.869318856
26	C26	1.142599202	1.901370133	0.409781223
27	C27	0.534811749	-1.750096972	-1.046635129
28	C28	4.128461408	1.199312754	-1.200196506
29	C29	2.550457720	-0.759228369	-5.665168049
30	C30	2.794313052	1.537753095	-4.739220291
31	H31	-2.551672787	-1.306150301	1.511380956
32	H32	-0.400653839	-1.046479364	1.318171304
33	O33	-5.892542728	-0.847591662	4.247461962
34	H34	1.759795173	1.355112821	-2.034570856
35	H35	-2.425000571	-1.486498988	5.769982698
36	H36	-3.827634492	-0.405361829	5.934015984
37	H37	-4.071255599	-2.134917532	5.779912535
38	H38	3.603467305	1.768149469	-5.441690919
39	H39	1.847184759	1.823108332	-5.212586861
40	H40	1.572767321	-0.519773256	-6.100805582
41	H41	2.575974674	-1.837260760	-5.464182579
42	H42	3.316867770	-0.541220913	-6.417983783
43	H43	-2.417761769	2.091183447	3.407830706
44	H44	-4.089758098	1.540982410	3.523926507

45	H45	-2.987091722	1.315687179	4.876298504
46	H46	-1.673834389	2.522587912	-0.081342395
47	H47	-1.374280952	2.565172285	1.647881634
48	H48	0.381966794	2.464086497	-0.128730887
49	H49	1.160253055	2.276075747	1.438053892
50	H50	2.101038382	2.161975222	-0.032505943
51	H51	1.504687135	-2.191303393	-1.292517442
52	H52	0.264804869	-2.133567477	-0.062024549
53	H53	-6.230249703	-0.788633814	1.752039498
54	H54	-4.320553555	-0.196139134	0.181431856
55	H55	4.827799246	0.500599317	-3.695115636
56	H56	4.676541057	-1.083893259	-4.418269633
57	H57	0.861790255	0.125058490	3.152640878
58	H58	-0.230868539	1.477636142	2.981507830
59	H59	-0.960679457	-1.430894871	3.567695708
60	H60	-1.020533404	-0.100185721	4.716988125
61	H61	-2.776022460	0.530127119	-1.074081372
62	H62	-2.043216606	-1.015781417	-0.673266250
63	H63	-0.590758431	1.484002691	-1.666692812
64	H64	-0.927731636	0.011156782	-2.533709875
65	H65	1.887778686	-1.150701120	1.627804798
66	H66	2.650041418	0.416546685	1.691515687
67	H67	5.008717434	-1.223895548	-1.959270371
68	H68	3.495619420	-1.954833749	-2.451077042
69	H69	4.210960891	-0.739450858	0.435621223
70	H70	3.049024093	-1.866385480	-0.187896410
71	H71	0.726208311	0.108950110	-3.819484585
72	H72	1.566754106	-1.369286788	-3.368142306
73	H73	-3.202229153	-2.144915724	3.484519981
74	H74	3.902995857	1.720319430	-0.266593548
75	H75	5.190633079	0.928823090	-1.157201513
76	H76	4.003696141	1.916369482	-2.017179445
77	H77	2.929579290	2.173462954	-3.858815406
78	H78	-4.977289638	-2.035704852	1.613373787
79	H79	-4.563976198	0.982541292	1.453448301
80	H80	-3.002746155	2.294929697	1.034374122
81	O81	-0.444592705	-2.293450124	-1.915247087
82	H82	-0.186600825	-2.092848148	-2.821712798

---

7 lowest energy conformers were considered for Boltzmann average to contribute to the ^{13}C NMR data

III.19. Cartesian coordinates of the global minimum conformer and chemical shifts published by Giner et al.{Giner, 1993 #15} for kokoonol[26-OH] (7b)

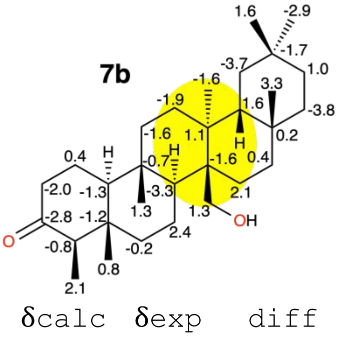

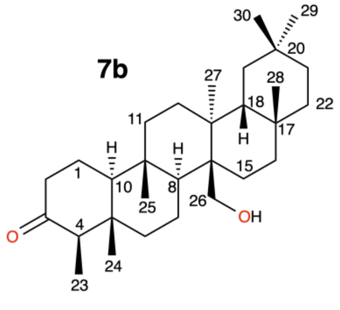

no δcalc δexp diff

C1	22.9	22.5	0.4	
C2	39.6	41.6	-2.0	
C3	210.5	213.3	-2.8	
C4	57.6	58.4	-0.8	
C5	41.1	42.3	-1.2	
C6	41.3	41.5	-0.2	
C7	21.0	18.6	2.4	
C8	50.5	53.8	-3.3	
C9	36.9	37.6	-0.7	
C10	58.4	59.7	-1.3	
C11	36.2	37.8	-1.6	
C12	30.3	32.2	-1.9	*
C13	39.5	38.4	1.1	*
C14	43.8	45.4	-1.6	*
C15	26.2	24.1	2.1	*
C16	36.7	36.3	0.4	
C17	30.5	30.3	0.2	
C18	44.9	43.3	1.6	
C19	33.4	37.1	-3.7	
C20	26.8	28.5	-1.7	
C21	33.6	32.6	1.0	
C22	36.3	40.1	-3.8	
C23	9.2	7.1	2.1	
C24	15.7	14.9	0.8	
C25	19.5	18.2	1.3	
C26	64.7	63.4	1.3	*
C27	20.7	22.3	-1.6	*
C28	33.9	30.6	3.3	*
C29	32.9	35.8	-2.9	
C30	34.4	32.8	1.6	*
RMSD		2.0		
Max Abs		3.8		

mol2 coordinates for lowest energy conformer

1	C1	4.214352246	-0.090848703	1.026286324
2	C2	5.237587333	0.933613130	1.537930587
3	C3	5.107733768	1.163538590	3.029080556
4	C4	3.694289365	1.472034552	3.507423644
5	C5	2.730097180	0.312993893	3.070590263
6	C6	1.298283323	0.682170873	3.502045905
7	C7	0.209764381	-0.157226357	2.834271933
8	C8	0.308664350	-0.041223892	1.307283215
9	C9	1.671369597	-0.614647859	0.803463381
10	C10	2.791329431	0.230622182	1.509213257
11	C11	1.772532399	-0.367891733	-0.720040865
12	C12	0.527146692	-0.761284055	-1.519599052
13	C13	-0.775794392	-0.106127911	-1.018084623
14	C14	-0.973201055	-0.467690395	0.509062122
15	C15	-2.177127315	0.354586014	1.015968893
16	C16	-3.462781994	0.314540175	0.153595303
17	C17	-3.399123135	-0.104879289	-1.354420148
18	C18	-2.009111478	-0.669209073	-1.799723664
19	C19	-1.918796398	-0.758039900	-3.353556381
20	C20	-2.499054310	0.351148529	-4.268325112
21	C21	-3.863893300	0.808506537	-3.727480202
22	C22	-3.839342839	1.097059749	-2.227796203

23	C23	3.684811674	1.830678818	4.991130904
24	C24	3.160802446	-0.985061566	3.776107693
25	C25	1.849651618	-2.131654474	1.030706034
26	C26	-1.227404768	-1.977011994	0.764493220
27	C27	-0.614323219	1.416203997	-1.204231529
28	C28	-4.445818747	-1.224728840	-1.552371997
29	C29	-1.578648759	1.571028637	-4.435574470
30	C30	-2.687449693	-0.266731099	-5.663592244
31	H31	2.588326634	1.260951825	1.171597965
32	H32	0.389793369	1.038236732	1.133621501
33	O33	6.057436560	1.101908786	3.781393793
34	H34	-2.024966387	-1.723520993	-1.501622863
35	H35	2.686670971	2.128744257	5.322587296
36	H36	4.030719296	1.001721537	5.613440195
37	H37	4.366762323	2.665608728	5.172260937
38	H38	-3.138622008	0.457097517	-6.352809869
39	H39	-1.725883118	-0.580564796	-6.088640861
40	H40	-0.538635000	1.267907058	-4.608725945
41	H41	-1.599975848	2.235233126	-3.570018297
42	H42	-1.899245961	2.164003756	-5.300485255
43	H43	2.493291178	-1.817176485	3.549002593
44	H44	4.176856017	-1.293386729	3.508805302
45	H45	3.143148520	-0.846173581	4.861387737
46	H46	1.375269161	-2.708663127	0.233085562
47	H47	1.433291537	-2.488744215	1.974121976
48	H48	-0.735574674	-2.591768650	0.000336381
49	H49	-0.781941645	-2.247697504	1.728307847
50	H50	0.136847653	1.850974679	-0.542620959
51	H51	-0.282666665	1.624024106	-2.222166774
52	H52	6.263532262	0.629279148	1.315140575
53	H53	4.253590311	-0.098623845	-0.066222288
54	H54	-4.616007722	0.039160294	-3.940920964
55	H55	-4.187552158	1.705658741	-4.272058116
56	H56	-0.760951616	0.203694400	3.184552573
57	H57	0.283692805	-1.196922843	3.172231248
58	H58	1.115944271	1.738859067	3.256112819
59	H59	1.208975953	0.596556867	4.591021335
60	H60	2.623285166	-0.926665524	-1.127571372
61	H61	1.992603569	0.692191674	-0.902193432
62	H62	0.418262308	-1.853039044	-1.540584011
63	H63	0.708911622	-0.468968918	-2.559022575
64	H64	-1.838052035	1.391038270	1.127013339
65	H65	-2.453480537	0.020784427	2.020242809
66	H66	-4.846103446	1.394870625	-1.906197682
67	H67	-3.200805174	1.965741539	-2.035163389
68	H68	-4.154821236	-0.368390329	0.647838960
69	H69	-3.928017568	1.306550304	0.213913783
70	H70	-2.467845406	-1.669720894	-3.619663571
71	H71	-0.888593519	-0.960111460	-3.665284853
72	H72	3.370717533	2.358663055	2.939644292
73	H73	-4.231755945	-2.050159885	-0.870688761
74	H74	-5.452956711	-0.847465259	-1.336019033
75	H75	-4.454901074	-1.620261850	-2.572708288
76	H76	-3.341629302	-1.145802656	-5.625005464
77	H77	5.056462526	1.897997200	1.041020593
78	H78	4.521889468	-1.090644457	1.352043672

79	H79	2.908868928	-2.403327169	1.020169689
80	O80	-2.611818247	-2.287406509	0.812499151
81	H81	-2.688418166	-3.230460631	0.991941322
82	H82	-1.540473176	1.963741098	-1.032797367

---

7 lowest energy conformers were considered for Boltzmann average to contribute to the 13C NMR data

III.20. Cartesian coordinates of the global minimum conformer and chemical shifts published by Gunatilaka et al.{Gunatilaka, 1982 #23} for kokoonol [26-OH] (7b)

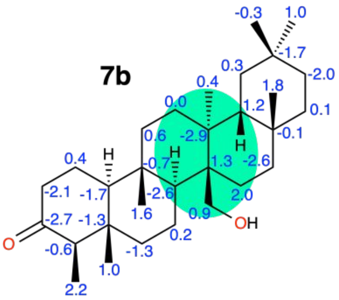

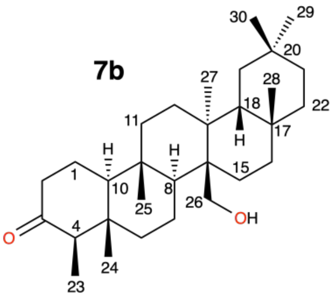

no	δ_{calc}	δ_{exp}	diff	
C1	22.9	22.5	0.4	
C2	39.6	41.7	-2.1	*
C3	210.5	213.2	-2.7	
C4	57.6	58.2	-0.6	*
C5	41.1	42.4	-1.3	
C6	41.3	42.6	-1.3	*
C7	21.0	20.8	0.2	
C8	50.5	53.1	-2.6	
C9	36.9	37.6	-0.7	
C10	58.4	60.1	-1.7	*
C11	36.2	35.6	0.6	
C12	30.3	30.3	0.0	*
C13	39.5	42.4	-2.9	
C14	43.8	42.5	1.3	
C15	26.2	24.2	2.0	*
C16	36.7	39.3	-2.6	*
C17	30.5	30.6	-0.1	
C18	44.9	43.7	1.2	
C19	33.4	33.1	0.3	*
C20	26.8	28.5	-1.7	
C21	33.6	35.6	-2.0	*
C22	36.3	36.2	0.1	*
C23	9.2	7.0	2.2	
C24	15.7	14.7	1.0	
C25	19.5	17.9	1.6	*
C26	64.7	64.3	0.4	
C27	20.7	19.8	0.9	*
C28	33.9	32.1	1.8	
C29	32.9	31.9	1.0	*
C30	34.4	34.7	-0.3	*
RMSD		1.5		
Max Abs		2.9		

mol2 coordinates for lowest energy conformer

1	C1	4.214352246	-0.090848703	1.026286324
2	C2	5.237587333	0.933613130	1.537930587
3	C3	5.107733768	1.163538590	3.029080556
4	C4	3.694289365	1.472034552	3.507423644
5	C5	2.730097180	0.312993893	3.070590263
6	C6	1.298283323	0.682170873	3.502045905
7	C7	0.209764381	-0.157226357	2.834271933
8	C8	0.308664350	-0.041223892	1.307283215
9	C9	1.671369597	-0.614647859	0.803463381
10	C10	2.791329431	0.230622182	1.509213257
11	C11	1.772532399	-0.367891733	-0.720040865
12	C12	0.527146692	-0.761284055	-1.519599052
13	C13	-0.775794392	-0.106127911	-1.018084623
14	C14	-0.973201055	-0.467690395	0.509062122
15	C15	-2.177127315	0.354586014	1.015968893
16	C16	-3.462781994	0.314540175	0.153595303
17	C17	-3.399123135	-0.104879289	-1.354420148
18	C18	-2.009111478	-0.669209073	-1.799723664
19	C19	-1.918796398	-0.758039900	-3.353556381
20	C20	-2.499054310	0.351148529	-4.268325112
21	C21	-3.863893300	0.808506537	-3.727480202
22	C22	-3.839342839	1.097059749	-2.227796203
23	C23	3.684811674	1.830678818	4.991130904
24	C24	3.160802446	-0.985061566	3.776107693
25	C25	1.849651618	-2.131654474	1.030706034
26	C26	-1.227404768	-1.977011994	0.764493220
27	C27	-0.614323219	1.416203997	-1.204231529
28	C28	-4.445818747	-1.224728840	-1.552371997
29	C29	-1.578648759	1.571028637	-4.435574470
30	C30	-2.687449693	-0.266731099	-5.663592244
31	H31	2.588326634	1.260951825	1.171597965
32	H32	0.389793369	1.038236732	1.133621501
33	O33	6.057436560	1.101908786	3.781393793
34	H34	-2.024966387	-1.723520993	-1.501622863
35	H35	2.686670971	2.128744257	5.322587296
36	H36	4.030719296	1.001721537	5.613440195
37	H37	4.366762323	2.665608728	5.172260937
38	H38	-3.138622008	0.457097517	-6.352809869
39	H39	-1.725883118	-0.580564796	-6.088640861
40	H40	-0.538635000	1.267907058	-4.608725945
41	H41	-1.599975848	2.235233126	-3.570018297
42	H42	-1.899245961	2.164003756	-5.300485255
43	H43	2.493291178	-1.817176485	3.549002593
44	H44	4.176856017	-1.293386729	3.508805302
45	H45	3.143148520	-0.846173581	4.861387737
46	H46	1.375269161	-2.708663127	0.233085562
47	H47	1.433291537	-2.488744215	1.974121976
48	H48	-0.735574674	-2.591768650	0.000336381
49	H49	-0.781941645	-2.247697504	1.728307847
50	H50	0.136847653	1.850974679	-0.542620959
51	H51	-0.282666665	1.624024106	-2.222166774
52	H52	6.263532262	0.629279148	1.315140575
53	H53	4.253590311	-0.098623845	-0.066222288
54	H54	-4.616007722	0.039160294	-3.940920964
55	H55	-4.187552158	1.705658741	-4.272058116
56	H56	-0.760951616	0.203694400	3.184552573

57	H57	0.283692805	-1.196922843	3.172231248
58	H58	1.115944271	1.738859067	3.256112819
59	H59	1.208975953	0.596556867	4.591021335
60	H60	2.623285166	-0.926665524	-1.127571372
61	H61	1.992603569	0.692191674	-0.902193432
62	H62	0.418262308	-1.853039044	-1.540584011
63	H63	0.708911622	-0.468968918	-2.559022575
64	H64	-1.838052035	1.391038270	1.127013339
65	H65	-2.453480537	0.020784427	2.020242809
66	H66	-4.846103446	1.394870625	-1.906197682
67	H67	-3.200805174	1.965741539	-2.035163389
68	H68	-4.154821236	-0.368390329	0.647838960
69	H69	-3.928017568	1.306550304	0.213913783
70	H70	-2.467845406	-1.669720894	-3.619663571
71	H71	-0.888593519	-0.960111460	-3.665284853
72	H72	3.370717533	2.358663055	2.939644292
73	H73	-4.231755945	-2.050159885	-0.870688761
74	H74	-5.452956711	-0.847465259	-1.336019033
75	H75	-4.454901074	-1.620261850	-2.572708288
76	H76	-3.341629302	-1.145802656	-5.625005464
77	H77	5.056462526	1.897997200	1.041020593
78	H78	4.521889468	-1.090644457	1.352043672
79	H79	2.908868928	-2.403327169	1.020169689
80	O80	-2.611818247	-2.287406509	0.812499151
81	H81	-2.688418166	-3.230460631	0.991941322
82	H82	-1.540473176	1.963741098	-1.032797367

---

7 lowest energy conformers were considered for Boltzmann average to contribute to the ^{13}C NMR data

III.21. Cartesian coordinates of the global minimum conformer of Trichadenic acid B (9a).

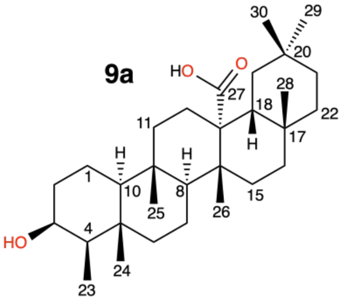

IUPAC	δ_{calc}	δ_{exp}	diff
C1	17.4	17.8	-0.4
C2	35.0	35.0	0.0
C3	72.2	72.7	-0.5
C4	48.6	49.0	-0.4
C5	37.5	37.3	0.2
C6	41.1	41.4	-0.3
C7	19.3	15.8	3.5
C8	51.7	53.2	-1.5
C9	36.5	37.9	-1.4
C10	60.3	61.2	-0.9
C11	38.6	37.9	0.7
C12	28.9	27.8	1.1
C13	53.8	54.8	-1.0

C14	40.9	39.3	1.6
C15	31.5	32.8	-1.3
C16	35.2	35.6	-0.4
C17	31.0	36.7	-5.7
C18	44.3	43.3	1.0
C19	34.8	35.7	-0.9
C20	28.4	28.5	-0.1
C21	32.6	32.5	0.1
C22	36.4	38.2	-1.8
C23	12.7	11.6	1.1
C24	17.7	16.4	1.3
C25	21.8	18.8	3.0
C26	21.7	22.5	-0.8
C27	179.6	179.4	0.2
C28	31.2	31.1	0.1
C29	33.0	35.3	-2.3
C30	32.0	30.6	1.4
RMSD		1.6	
Max Abs		5.7	

mol2 coordinates for lowest energy conformer

1	C1	-4.101286539	0.090227197	1.099691469
2	C2	-5.035498203	-0.942107712	1.727393049
3	C3	-4.951763409	-0.951358764	3.246569957
4	C4	-3.492861484	-1.051661952	3.739307969
5	C5	-2.527632888	0.007430500	3.112285715
6	C6	-1.080778678	-0.339966696	3.526695226
7	C7	-0.011644519	0.374059188	2.698527707
8	C8	-0.193169781	0.049610382	1.212135106
9	C9	-1.566594811	0.593790799	0.700585359
10	C10	-2.654143012	-0.140182810	1.558976395
11	C11	-1.732840653	0.162408799	-0.774599623
12	C12	-0.522344797	0.462415751	-1.664375183
13	C13	0.779149037	-0.186655627	-1.136315887
14	C14	1.055334317	0.339350048	0.320560501
15	C15	2.314752977	-0.373660334	0.904568061
16	C16	3.274086797	-0.991907548	-0.129327998
17	C17	3.408859278	-0.254310412	-1.474399761
18	C18	2.004062649	0.175263127	-2.060138311
19	C19	1.818802750	-0.286647819	-3.520286363
20	C20	3.002536888	-0.006038023	-4.465793746
21	C21	4.317407111	-0.566995398	-3.845212014
22	C22	4.096939933	-1.206430695	-2.473471219
23	C23	-3.450344577	-1.136716674	5.269766708
24	C24	-2.852941411	1.420484748	3.633669009
25	C25	-1.736294319	2.131473121	0.752089000
26	C26	1.354755923	1.855709851	0.317352913
27	C27	0.506342822	-1.706513036	-1.129513127
28	C28	4.341278330	0.958767160	-1.262310321
29	C29	2.716278732	-0.724286004	-5.793095550
30	C30	3.123549729	1.496967937	-4.755376177
31	H31	-2.455572767	-1.209077026	1.369336892
32	H32	-0.299469005	-1.041123929	1.177702770
33	H33	1.983691016	1.270685594	-2.085620834
34	H34	-2.448548914	-1.372594890	5.635402398
35	H35	-3.755824386	-0.204530799	5.757163437

36	H36	-4.119724982	-1.929961396	5.621282567
37	H37	3.965209286	1.694670019	-5.429845821
38	H38	2.212506720	1.874674974	-5.235829765
39	H39	1.771051494	-0.383430295	-6.233118563
40	H40	2.646618748	-1.808662395	-5.644270546
41	H41	3.515159583	-0.535410349	-6.520359238
42	H42	-2.115657894	2.153061328	3.303952355
43	H43	-3.842193664	1.762380190	3.328649534
44	H44	-2.827220220	1.429619997	4.728429796
45	H45	-1.460804021	2.597771935	-0.198047860
46	H46	-1.135814200	2.613154301	1.526084621
47	H47	0.610820497	2.454345535	-0.206711867
48	H48	1.391074902	2.215241425	1.350520431
49	H49	2.321842583	2.084961411	-0.126406394
50	H50	-6.074811085	-0.757641558	1.435254933
51	H51	-4.175325352	0.006880878	0.011944194
52	H52	5.060277065	0.234557687	-3.754315953
53	H53	0.973219296	0.045628726	3.045989755
54	H54	-0.049728623	1.453782350	2.883448334
55	H55	-0.927250632	-1.424042357	3.420312259
56	H56	-0.934168267	-0.105089701	4.587999117
57	H57	-2.604903883	0.660070675	-1.213818207
58	H58	-1.945369388	-0.915155836	-0.818019100
59	H59	2.005189226	-1.183933595	1.570231242
60	H60	2.857042963	0.348061980	1.527437912
61	H61	5.058789840	-1.528856895	-2.053821006
62	H62	3.494337241	-2.118338913	-2.581127403
63	H63	4.273785952	-1.068869991	0.318143262
64	H64	2.957053451	-2.018972656	-0.322336109
65	H65	0.926375096	0.190686608	-3.941010747
66	H66	1.616912997	-1.360106356	-3.548981454
67	H67	-3.143248745	-2.028806843	3.370459367
68	H68	4.117645933	1.491351307	-0.334816653
69	H69	5.380970299	0.619052215	-1.185411392
70	H70	4.292812323	1.684243022	-2.078904831
71	H71	3.284348333	2.084397022	-3.845683896
72	H72	-4.766876026	-1.942176729	1.362405157
73	H73	-4.448646575	1.094755618	1.358488821
74	H74	-2.780398319	2.398772125	0.936521518
75	O75	0.726907718	-2.503640784	-0.244249530
76	H76	4.761531348	-1.309398854	-4.519166871
77	H77	-0.743035301	0.101568912	-2.670376239
78	H78	-0.365501668	1.542092421	-1.755196876
79	O79	-0.092764345	-2.134040596	-2.264512096
80	H80	-0.229501338	-3.086744668	-2.134982434
81	H81	-5.481356089	-1.845059281	3.619566756
82	O82	-5.633852764	0.209826803	3.701558477
83	H83	-5.551603651	0.246852531	4.662696915

---

10 lowest energy conformers were considered for Boltzmann average to contribute to the ^{13}C NMR data

III.22. Cartesian coordinates of the global minimum conformer of Pristimeronol (10a).

10a

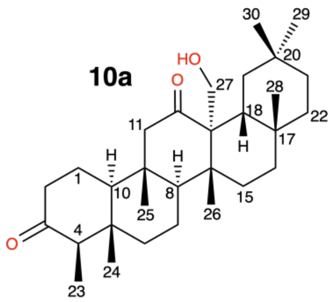

no	δ_{calc}	δ_{exp}	diff	IUPAC no
C1	23.2	22.6	0.6	1
C2	39.9	41.3	-1.4	2
C3	210.0	211.6	-1.6	3
C4	57.4	57.9	-0.5	4
C5	41.6	42.2	-0.6	5
C6	40.8	40.9	-0.1	6
C7	20.1	18.9	1.2	7
C8	53.5	54.1	-0.6	8
C9	42.2	43.7	-1.5	9
C10	58.6	59.3	-0.7	10
C11	55.5	56.1	-0.6	11
C12	213.8	211.4	2.4	12
C13	61.7	62.3	-0.6	13
C14	43.1	43.7	-0.6	14
C15	32.8	33.1	-0.3	15
C16	35.2	37.2	-2	16
C17	30.2	30.4	-0.2	17
C18	37.8	37.6	0.2	18
C19	36.7	36.3	0.4	19
C20	28.6	28.7	-0.1	20
C21	31.9	33.3	-1.4	21
C22	39.9	40.4	-0.5	22
C23	8.8	7.3	1.5	23
C24	15.5	14.6	0.9	24
C25	20.2	18.3	1.9	25
C26	22.6	22.3	0.3	26
C27	65.0	62.5	2.5	27
C28	31.4	30.6	0.8	28
C29	34.1	35.6	-1.5	29
C30	29.6	31.9	-2.3	30
RMSD		1.2		
Max Abs		2.5		

mol2 coordinates for lowest energy conformer

1	C1	-4.197454151	-1.119155196	0.749782048
2	C2	-4.961391355	-2.345672684	1.268494741
3	C3	-4.904171643	-2.436849596	2.778843824
4	C4	-3.504333525	-2.341986058	3.376227803
5	C5	-2.822059724	-1.007643912	2.910576426
6	C6	-1.379403650	-0.977543447	3.454854578
7	C7	-0.482813874	0.065750603	2.787528762
8	C8	-0.443419989	-0.169011060	1.273028359
9	C9	-1.865912645	0.034729879	0.663763909
10	C10	-2.784608866	-1.036913682	1.345795882
11	C11	-1.784290409	-0.284403349	-0.851122183
12	C12	-0.628045638	0.442894954	-1.511759304

13	C13	0.771532079	0.080611576	-0.991578340
14	C14	0.746592930	0.516647347	0.530807233
15	C15	2.087305617	0.089883790	1.195213269
16	C16	3.283172068	-0.097167691	0.240520557
17	C17	3.307212494	0.785669336	-1.025091619
18	C18	1.895239888	0.858999179	-1.740423865
19	C19	1.987145066	0.433109666	-3.219448657
20	C20	3.091679818	1.127171624	-4.038488723
21	C21	4.458240519	0.972572966	-3.304717233
22	C22	4.342645736	0.183220961	-1.999875488
23	C23	-3.538552873	-2.587929476	4.882336231
24	C24	-3.607497922	0.182731299	3.489027939
25	C25	-2.437296449	1.468729602	0.788016470
26	C26	0.614883654	2.051982355	0.640203896
27	C27	0.939435203	-1.459322239	-1.202373565
28	C28	3.824967233	2.184432157	-0.617612693
29	C29	3.162128853	0.442925189	-5.411132732
30	C30	2.750514510	2.608093541	-4.253919482
31	H31	-2.302344166	-1.996738524	1.095730670
32	H32	-0.256934172	-1.246992274	1.172221910
33	O33	-5.898949601	-2.566575831	3.460667952
34	H34	1.573967563	1.904147357	-1.753554156
35	H35	-2.534013561	-2.570943392	5.313503323
36	H36	-4.158979612	-1.852673951	5.400204735
37	H37	-3.978373069	-3.567937386	5.085405762
38	H38	3.526687505	3.097321228	-4.854017383
39	H39	1.794756205	2.716801670	-4.779204939
40	H40	2.199451862	0.502122883	-5.933071552
41	H41	3.427783486	-0.616677179	-5.309590218
42	H42	3.919192653	0.918125736	-6.045824570
43	H43	-3.144736247	1.138705021	3.243906545
44	H44	-4.643247108	0.214673612	3.137526559
45	H45	-3.641703440	0.113433690	4.580311267
46	H46	-2.192757055	2.067341212	-0.092385784
47	H47	-2.072331017	2.012091118	1.661072316
48	H48	-0.172399168	2.460216184	0.006524487
49	H49	0.397410110	2.334509049	1.674669785
50	H50	1.533616767	2.561261443	0.361139879
51	H51	1.996154500	-1.704683433	-1.341754375
52	H52	0.598439980	-2.027943814	-0.336215734
53	H53	-6.007824747	-2.335270747	0.953926600
54	H54	-4.146444019	-1.180139660	-0.340725571
55	H55	4.880706825	1.961326012	-3.090915900
56	H56	0.519749810	-0.011877596	3.219049210
57	H57	-0.835803729	1.074570326	3.026537359
58	H58	-0.923324908	-1.967546188	3.305796737
59	H59	-1.399526598	-0.804631810	4.536618426
60	H60	-2.692463136	0.031176495	-1.370363187
61	H61	-1.654112559	-1.358977510	-1.010466331
62	H62	1.947668954	-0.857123670	1.730843503
63	H63	2.338751162	0.829379649	1.963951160
64	H64	5.319602576	0.144616082	-1.501130997
65	H65	4.080225204	-0.859856108	-2.221848757
66	H66	4.210685153	0.082492351	0.798751337
67	H67	3.335459783	-1.149092590	-0.057079313
68	H68	1.020376891	0.623367213	-3.697491746

69	H69	2.185972897	-0.645252038	-3.289134838
70	H70	-2.922203542	-3.152845028	2.910157440
71	H71	3.396186361	2.526712323	0.326881694
72	H72	4.911133404	2.152139997	-0.472269592
73	H73	3.615631816	2.948167313	-1.371616589
74	H74	2.670207111	3.153712682	-3.308568541
75	H75	-4.494463712	-3.254471264	0.863264395
76	H76	-4.775881722	-0.218273608	0.980277167
77	H77	-3.527841740	1.447035851	0.849964643
78	O78	0.186784196	-1.968976148	-2.290712867
79	H79	0.507227799	-1.554073072	-3.100278272
80	H80	5.182154094	0.474775340	-3.960388024
81	O81	-0.820768997	1.312793351	-2.337773664

---

3 lowest energy conformers were considered for Boltzmann average to contribute to the ^{13}C NMR data

III.23. Cartesian coordinates of the global minimum conformer of salasone A (10b). {Morikawa, 2003 #36}

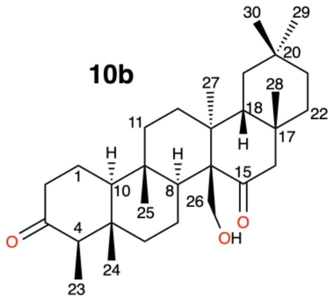

no	δ_{calc}	δ_{exp}	diff	IUPAC no
C1	23.0	22.2	0.8	1
C2	40.0	41.3	-1.3	2
C3	210.0	212.4	-2.4	3
C4	57.7	58.1	-0.4	4
C5	41.5	41.9	-0.4	5
C6	40.4	40.8	-0.4	6
C7	21.4	21.4	0.0	7
C8	45.8	45.3	0.5	8
C9	36.8	37.4	-0.6	9
C10	58.7	59.2	-0.5	10
C11	34.7	34.0	0.7	11
C12	32.0	30.9	1.1	12
C13	42.3	42.4	-0.1	13
C14	58.4	59.8	-1.4	14
C15	213.6	211.6	2.0	15
C16	52.9	54.3	-1.4	16
C17	32.7	32.6	0.1	17
C18	44.7	44.1	0.6	18
C19	35.8	35.7	0.1	19
C20	28.0	28.0	0.0	20
C21	32.2	32.9	-0.7	21
C22	39.3	39.3	0.0	22
C23	8.8	6.8	2.0	23
C24	16.1	15.0	1.1	24
C25	19.5	17.6	1.9	25
C26	62.1	60.5	1.6	26

C27	21.5	19.9	1.6	27
C28	32.0	32.5	-0.5	28
C29	33.4	34.6	-1.2	29
C30	31.2	31.5	-0.3	30
RMSD		1.1		
Max Abs		2.4		

mol2 coordinates for lowest energy conformer

1	C1	-4.244300061	-0.142829324	1.155930782
2	C2	-5.193976109	-1.109441973	1.878142413
3	C3	-5.009741437	-1.061283504	3.381209656
4	C4	-3.569149421	-1.203500086	3.858189193
5	C5	-2.685320009	-0.093683348	3.185255139
6	C6	-1.223623511	-0.289775541	3.635266409
7	C7	-0.191819809	0.430364357	2.766893122
8	C8	-0.342186822	-0.019338604	1.306011511
9	C9	-1.733554794	0.434834872	0.752896232
10	C10	-2.794795955	-0.309193440	1.636382907
11	C11	-1.863535516	-0.049868009	-0.707091745
12	C12	-0.665107950	0.324555894	-1.579068986
13	C13	0.666242460	-0.256311803	-1.072813819
14	C14	0.895473877	0.261631867	0.405335269
15	C15	2.097757077	-0.568992424	0.882427804
16	C16	3.314738374	-0.657846102	-0.021916634
17	C17	3.289691874	0.004598625	-1.419913875
18	C18	1.835456210	0.192847140	-2.014722570
19	C19	1.705947282	-0.452657151	-3.410424695
20	C20	2.813521808	-0.095220556	-4.421749680
21	C21	4.211774285	-0.345867819	-3.783154187
22	C22	4.117285821	-0.897976088	-2.362204073
23	C23	-3.503777288	-1.284778582	5.380916941
24	C24	-3.180173490	1.284509120	3.661271430
25	C25	-1.973611867	1.960892221	0.729496575
26	C26	1.288922069	1.763728895	0.502363505
27	C27	0.558605203	-1.803417415	-1.080957988
28	C28	4.020616282	1.363200296	-1.313615746
29	C29	2.637685975	-1.001771976	-5.648386105
30	C30	2.677435736	1.364082819	-4.879019443
31	H31	-2.555121217	-1.375123135	1.487195984
32	H32	-0.407183995	-1.111564950	1.368111705
33	O33	-5.941093312	-0.912107515	4.143757907
34	H34	1.697481375	1.267902083	-2.177974207
35	H35	-2.478592653	-1.436586556	5.727947685
36	H36	-3.909357088	-0.388077300	5.855222849
37	H37	-4.108363158	-2.125572886	5.731041052
38	H38	3.461645275	1.615921017	-5.602323524
39	H39	1.707810927	1.533753351	-5.362726106
40	H40	1.643233961	-0.879153832	-6.094416562
41	H41	2.756759293	-2.057098016	-5.376051974
42	H42	3.382776681	-0.767457382	-6.417555826
43	H43	-2.549325273	2.097504396	3.300670382
44	H44	-4.207562971	1.498389503	3.351183994
45	H45	-3.160359830	1.329195777	4.753979126
46	H46	-1.503084655	2.430255784	-0.137713783
47	H47	-1.606004014	2.483615346	1.612669766
48	H48	0.417452321	2.363501979	0.770788219

49	H49	1.650672184	2.152729264	-0.446964375
50	H50	0.005660202	-2.194450196	-0.224976032
51	H51	0.034709931	-2.142101388	-1.979949564
52	H52	-6.241318953	-0.901914856	1.644462725
53	H53	-4.315217824	-0.331765282	0.081296478
54	H54	4.790964086	0.583990540	-3.770716698
55	H55	0.798011977	0.159333403	3.138287268
56	H56	-0.300712639	1.518214600	2.868310149
57	H57	-0.980796742	-1.361483367	3.613200862
58	H58	-1.115449215	0.031625967	4.677268878
59	H59	-2.762958520	0.380122400	-1.162403506
60	H60	-2.000058428	-1.139162954	-0.725992812
61	H61	-0.575281475	1.416541529	-1.654845477
62	H62	-0.861103297	-0.030014734	-2.596113358
63	H63	5.123957680	-1.017711570	-1.941361942
64	H64	3.678253967	-1.904660745	-2.385273914
65	H65	4.150814474	-0.254107551	0.559821505
66	H66	3.516271974	-1.732326674	-0.103345057
67	H67	0.744252865	-0.173464206	-3.855086006
68	H68	1.686481948	-1.542846820	-3.313914089
69	H69	-3.206210565	-2.160189728	3.450554293
70	H70	3.697833969	1.936421858	-0.442777163
71	H71	5.098478750	1.195623657	-1.197320501
72	H72	3.880253033	1.980941227	-2.204996067
73	H73	2.760363317	2.068246352	-4.044666366
74	H74	-4.971382588	-2.135729132	1.552466853
75	H75	-4.594339033	0.882873439	1.311939466
76	H76	-3.043388203	2.174205932	0.653062138
77	H77	4.786121047	-1.045607888	-4.401162898
78	O78	2.339931821	1.997021715	1.427663770
79	H79	2.007832543	1.802772751	2.312503384
80	H80	1.538018694	-2.289721876	-1.076463212
81	O81	2.081741059	-1.232195689	1.900735232

---

6 lowest energy conformers were considered for Boltzmann average to contribute to the ^{13}C NMR data

III.24. Cartesian coordinates of the global minimum conformer of 3β-hydroxyolean-28(19β)-olide (11P)

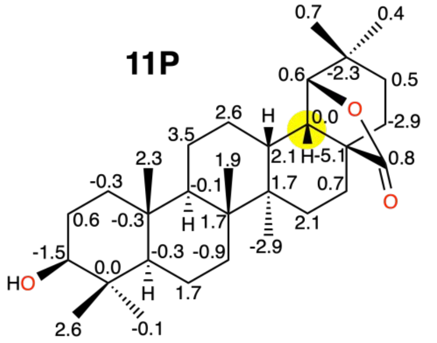

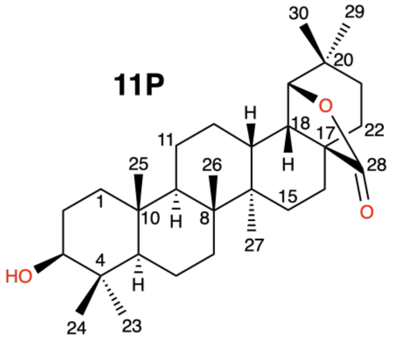

no	δ_{calc}	δ_{exp}	diff	IUPAC no	
C30	38.6	38.9	-0.3	1	
C32	27.9	27.3	0.6	2	
C33	77.3	78.8	-1.5	3	
C31	38.8	38.8	0.0	4	*

C29	55.1	55.4	-0.3	5	
C28	19.9	18.2	1.7	6	
C26	32.8	33.7	-0.9	7	
C23	42.4	40.7	1.7	8	
C25	51.1	51.2	-0.1	9	
C27	36.9	37.2	-0.3	10	
C24	24.3	20.8	3.5	11	
C21	30.4	27.8	2.6	12	*
C15	38.0	35.9	2.1	13	
C20	42.2	40.5	1.7	14	*
C17	28.5	26.4	2.1	15	*
C12	26.2	25.5	0.7	16	*
C10	40.9	46.0	-5.1	17	
C11	46.6	46.6	0.0	18	
C16	86.5	85.9	0.6	19	
C22	31.2	33.5	-2.3	20	
C19	32.7	32.2	0.5	21	*
C14	29.0	31.9	-2.9	22	*
C7	27.8	27.9	-0.1	23	*
C8	16.2	13.6	2.6	24	
C6	18.7	16.4	2.3	25	*
C5	17.2	15.3	1.9	26	
C2	21.0	23.9	-2.9	27	*
C13	180.6	179.8	0.8	28	
C3	25.8	25.4	0.4	29	*
C4	29.4	28.7	0.7	30	*
RMSD		1.9			
Max Abs		5.1			

mol2 coordinates for lowest energy conformer

1	O1	1.797293120	-1.202866782	6.011462547
2	C2	-1.029989465	0.898721619	1.563283507
3	C3	-1.763631744	-3.712887674	2.671442609
4	C4	-0.610133124	-4.950085263	4.494690264
5	C5	2.354130775	0.480345999	-0.404713318
6	C6	1.413029087	0.163619687	-3.347814423
7	C7	-0.699169769	4.358638153	-3.876399409
8	C8	1.016666941	2.919159065	-4.939033523
9	O9	-1.651262028	2.743220974	-6.131579555
10	C10	0.398592812	-0.856394291	4.001121949
11	C11	0.789973925	-1.686707718	2.765381797
12	C12	0.756627285	0.629978546	3.830711097
13	C13	1.278648708	-1.607949515	5.006783113
14	C14	-1.057787074	-1.106597950	4.471442970
15	C15	0.496139381	-1.174761406	1.323616574
16	C16	0.622385758	-3.092672749	3.374078818
17	C17	1.294730681	0.954206597	2.420497176
18	O18	1.403453096	-2.900268529	4.594417475
19	C19	-1.316517284	-2.565123179	4.869439479
20	C20	0.404228150	0.388235695	1.281203267
21	C21	-0.527817473	-1.789644175	0.374503701
22	C22	-0.775933291	-3.574140892	3.832208494
23	C23	0.869164683	0.842975459	-0.176871465
24	C24	-0.172857359	-1.358717536	-1.057138224
25	C25	-0.084850696	0.168404892	-1.228693808
26	C26	0.743327452	2.373629604	-0.331917581

27	C27	0.084507643	0.640261482	-2.718987030
28	C28	0.896163481	2.869243320	-1.771908282
29	C29	-0.107618609	2.190627733	-2.708565004
30	C30	-1.075534616	0.036350462	-3.547884477
31	C31	-0.265094098	2.896456897	-4.089315955
32	C32	-1.245472285	0.677634192	-4.924640708
33	C33	-1.419603343	2.189296791	-4.846239653
34	H34	1.884798051	-1.609846759	2.771677891
35	H35	1.442128901	-1.413131119	0.829145730
36	H36	-1.088447915	0.535634872	-0.985323334
37	H37	-1.093426895	2.332170771	-2.231697910
38	H38	-1.692007509	0.864483771	0.697511132
39	H39	-1.008514423	1.936336788	1.912062198
40	H40	-1.516397145	0.314900407	2.342057192
41	H41	-2.051034438	-2.746015529	2.253782848
42	H42	-1.355242020	-4.329667042	1.863286104
43	H43	-2.675832716	-4.203065083	3.029301711
44	H44	-1.558845104	-5.272637585	4.937615172
45	H45	0.145079754	-4.918779904	5.284257618
46	H46	-0.310147514	-5.706512409	3.759534663
47	H47	2.771499196	1.034125596	-1.244927675
48	H48	2.522481297	-0.578226050	-0.616724254
49	H49	2.959140314	0.744073110	0.466033542
50	H50	1.733326798	-0.788111654	-2.914666233
51	H51	2.231998114	0.873730102	-3.220147460
52	H52	1.307804922	-0.005878416	-4.422646995
53	H53	-1.494100222	4.433352811	-3.124623068
54	H54	-1.083268739	4.761022720	-4.817769976
55	H55	0.132756455	4.992764962	-3.554626604
56	H56	0.882532421	3.586718770	-5.796856744
57	H57	1.307520780	1.936691820	-5.318343013
58	H58	1.859877567	3.309680215	-4.361422543
59	H59	-0.924950327	2.464631368	-6.702086704
60	H60	-0.127330329	1.241641023	4.036819503
61	H61	1.502049423	0.913113816	4.581244509
62	H62	-1.753020369	-0.819600302	3.678970060
63	H63	-1.263689096	-0.451382010	5.325687011
64	H64	1.131320435	-3.873990059	2.802021920
65	H65	1.376500607	2.042472007	2.327053947
66	H66	2.314391845	0.563128314	2.340522455
67	H67	-2.391707049	-2.727021197	5.009168816
68	H68	-0.849174890	-2.764014061	5.840577916
69	H69	-1.554067722	-1.489572303	0.614070830
70	H70	-0.494634491	-2.881451860	0.426565435
71	H71	-0.927204932	-1.762910393	-1.737621703
72	H72	0.775274085	-1.838541461	-1.332989183
73	H73	-0.240229086	2.702740271	0.021934507
74	H74	1.486902314	2.872687297	0.301061743
75	H75	0.735516141	3.951380521	-1.773541120
76	H76	1.921977118	2.721112665	-2.127668060
77	H77	-2.014969544	0.155876520	-2.988707105
78	H78	-0.925440580	-1.040332183	-3.682021780
79	H79	-2.120641870	0.252822813	-5.428010175
80	H80	-0.382858798	0.443163134	-5.564135764
81	H81	-2.343483536	2.398041405	-4.289989474

---

4 lowest energy conformers were considered for Boltzmann average to contribute to the ^{13}C NMR data

III.25. Cartesian coordinates of the global minimum conformer of 28-oxyallobetulin (11R)

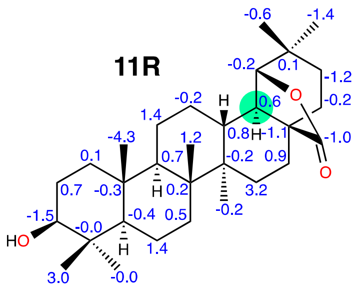

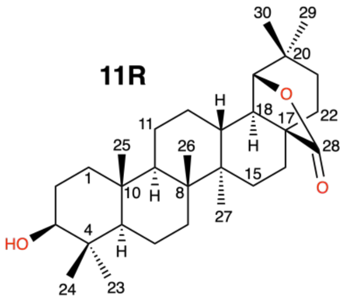

no	δ_{calc}	δ_{exp}	diff	IUPAC no	
C30	39.0	38.9	0.1	1	
C32	28.0	27.3	0.7	2	
C33	77.3	78.8	-1.5	3	
C31	38.8	38.8	0.0	4	*
C29	55.0	55.4	-0.4	5	
C28	19.6	18.2	1.4	6	
C26	34.2	33.7	0.5	7	
C23	40.9	40.7	0.2	8	
C25	51.9	51.2	0.7	9	
C27	36.9	37.2	-0.3	10	
C24	22.2	20.8	1.4	11	
C21	27.6	27.8	-0.2	12	*
C15	36.7	35.9	0.8	13	
C20	40.3	40.5	-0.2	14	*
C17	29.6	26.4	3.2	15	*
C12	26.4	25.5	0.9	16	*
C10	44.9	46.0	-1.1	17	
C11	47.2	46.6	0.6	18	
C16	85.7	85.9	-0.2	19	
C22	33.6	33.5	0.1	20	
C19	31.0	32.2	-1.2	21	*
C14	31.7	31.9	-0.2	22	*
C7	27.9	27.9	0.0	23	*
C8	16.6	13.6	3.0	24	*
C6	19.6	23.9	-4.3	25	*
C5	16.5	15.3	1.2	26	*
C2	16.2	16.4	-0.2	27	*
C13	178.8	179.8	-1.0	28	
C3	24.0	25.4	-1.4	29	*
C4	28.1	28.7	-0.6	30	*
RMSD		1.3			
Max Abs		4.3			

(The chemical shifts come from the compound 11P)

mol2 coordinates for lowest energy conformer

1	O1	-1.125298121	-2.505459686	4.517984165
2	C2	1.006370338	1.396959726	1.346021533
3	C3	-3.789886262	2.250521821	3.794394008
4	C4	-5.028200015	0.194535335	4.487473818

5	C5	0.573785610	-2.207014637	-0.185035719
6	C6	0.179247756	-1.650385980	-3.298360414
7	C7	4.330540882	0.436722648	-4.105165404
8	C8	2.895682289	-1.399818013	-4.950475942
9	O9	2.672732175	1.119289679	-6.427255802
10	C10	-0.839413203	-0.129979318	3.918355863
11	C11	-1.572519631	0.456648239	2.701085677
12	C12	0.667965921	-0.280083548	3.769231163
13	C13	-1.537904504	-1.488393425	4.029736531
14	C14	-1.236591458	0.694436105	5.165514447
15	C15	-1.074541605	-0.078454455	1.347002778
16	C16	-2.988116569	-0.024375034	3.050468088
17	C17	1.047576228	-0.860367238	2.405143589
18	O18	-2.777989931	-1.387259174	3.491519079
19	C19	-2.746666012	0.632686731	5.456163308
20	C20	0.477584163	-0.051302237	1.214916791
21	C21	-1.754994512	0.643239374	0.184741330
22	C22	-3.638431998	0.776407013	4.198908386
23	C23	0.901384579	-0.698026160	-0.183371624
24	C24	-1.352405519	0.035619807	-1.157137696
25	C25	0.171228079	0.064083562	-1.344515433
26	C26	2.424093455	-0.556891181	-0.397569228
27	C27	0.639684712	-0.255174468	-2.813239077
28	C28	2.884286960	-0.923943418	-1.808694381
29	C29	2.185316112	-0.042342790	-2.847550334
30	C30	0.010430560	0.804499080	-3.752178496
31	C31	2.869960986	-0.032056148	-4.247494635
32	C32	0.629908117	0.833400429	-5.149188195
33	C33	2.141082442	1.026320610	-5.114939571
34	H34	-1.524060930	1.548290248	2.697450485
35	H35	-1.379271786	-1.132322360	1.297435014
36	H36	0.457299036	1.117570555	-1.224821510
37	H37	2.319616658	0.992625214	-2.487095794
38	H38	0.871051858	1.992908254	0.441378901
39	H39	2.073789385	1.403542213	1.586566516
40	H40	0.503028846	1.935471260	2.153069000
41	H41	-2.837012359	2.788061883	3.793511026
42	H42	-4.230466756	2.344974807	2.794266809
43	H43	-4.453382405	2.762395356	4.499357576
44	H44	-5.462717230	0.663412751	5.378565484
45	H45	-4.971974536	-0.885055107	4.659948950
46	H46	-5.710184401	0.373713222	3.646653170
47	H47	0.832056457	-2.669671953	-1.135498455
48	H48	-0.482104690	-2.423379345	-0.003080452
49	H49	1.153093535	-2.730248214	0.580084667
50	H50	-0.736854558	-1.963442029	-2.789869237
51	H51	0.926415741	-2.431483904	-3.139815343
52	H52	-0.046914612	-1.647095223	-4.368218388
53	H53	4.406122239	1.312603189	-3.449340524
54	H54	4.717653037	0.716527872	-5.089031976
55	H55	4.977473276	-0.347242557	-3.699895607
56	H56	3.558933853	-1.360723914	-5.822537289
57	H57	1.911926219	-1.735131223	-5.289115575
58	H58	3.292965503	-2.171757957	-4.283229428
59	H59	2.401446594	0.326532612	-6.905542256
60	H60	1.146204391	0.693301028	3.929377860

61	H61	1.028976929	-0.946475048	4.560305147
62	H62	-0.912050244	1.728723390	4.993707562
63	H63	-0.684152257	0.327453256	6.037692291
64	H64	-3.663605374	-0.083412378	2.192543365
65	H65	2.139027565	-0.921205387	2.335136410
66	H66	0.671772706	-1.888040511	2.374188086
67	H67	-3.019972069	1.402943879	6.186790266
68	H68	-2.969283713	-0.330358615	5.931823928
69	H69	-1.488270563	1.709458973	0.203777699
70	H70	-2.845289100	0.598063243	0.293359834
71	H71	-1.846616210	0.591333027	-1.959005107
72	H72	-1.737042580	-0.990214920	-1.210186954
73	H73	2.730506079	0.480182301	-0.220684208
74	H74	2.962520581	-1.171505555	0.333017938
75	H75	3.967855231	-0.781567656	-1.860429530
76	H76	2.710584615	-1.987437469	-2.011813713
77	H77	0.127524067	1.799100541	-3.297591123
78	H78	-1.066298608	0.632594144	-3.854857325
79	H79	0.189002608	1.647469410	-5.734424967
80	H80	0.394991635	-0.092718779	-5.692560313
81	H81	2.349784313	2.007194227	-4.667851511

---

3 lowest energy conformers were considered for Boltzmann average to contribute to the ^{13}C NMR data

III.26. Cartesian coordinates of the global minimum conformer of 1β,30-Dihydroxy-3-oxo-D:A-friedooleanane (13a)

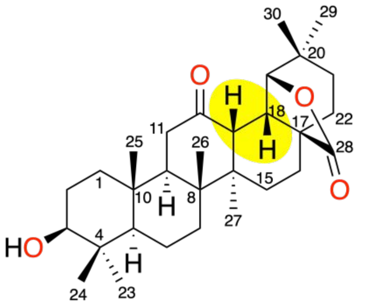

no	δ_{calc}	δ_{exp}	diff	IUPAC no
C30	37.4	38.5	-1.1	1
C32	27.7	27.8	-0.1	2
C33	77.1	77.9	-0.8	3
C31	38.7	39.4	-0.7	4
C29	54.7	55.6	-0.9	5
C28	19.8	18.4	1.4	6
C26	32.3	34.8	-2.5	7
C23	43.5	41.7	1.8	8
C25	50.4	49.8	0.6	9
C27	37.0	38.3	-1.3	10
C24	37.6	38.5	-0.9	11
C1	209.1	214.9	-5.8	12
C15	52.3	54.4	-2.1	13
C20	44.9	44.8	0.1	14
C17	28.9	26.1	2.8	15
C12	30.8	26.0	4.8	16
C10	40.8	42.0	-1.2	17
C11	43.6	45.8	-2.2	18
C16	88.1	87.2	0.9	19

C22	30.9	32.4	-1.5	20	
C19	31.6	33.6	-2.0	21	
C14	24.5	26.8	-2.3	22	
C7	28.0	28.6	-0.6	23	
C8	16.4	39.4		24	**
C6	17.2	15.9	1.3	25	
C5	18.8	17.7	1.1	26	
C2	25.7	25.8	-0.1	27	
C13	178.3	180.1	-1.8	28	
C3	25.9	22.7	3.2	29	*
C4	29.6	30.5	-0.9	30	*
RMSD		2.0			
Max Abs		5.8			

** The chemical shift assigned to C-24 (non-IUPAC) (39.4 ppm) must be a typographical error and has therefore been excluded from the calculation of RMSD and Max Abs.

mol2 coordinates for lowest energy conformer

1	O1	-1.586271907	-2.173566873	5.925286143
2	C2	0.093381808	1.429357676	1.255606937
3	C3	-3.272993460	1.946960244	2.650596204
4	C4	-4.748202879	1.127564618	4.478454834
5	C5	0.950366100	-2.188096569	-0.046074205
6	C6	0.624843621	-1.815330618	-3.116205843
7	C7	4.026530821	1.371679765	-3.883820974
8	C8	3.250084621	-0.835841606	-4.699975277
9	O9	2.362244352	1.446697522	-6.297693938
10	C10	-0.984824327	-0.788391284	3.969460844
11	C11	-1.887448329	-0.964362191	2.741243343
12	C12	0.387172474	-1.354096031	3.649944807
13	C13	-1.881062330	-1.531625201	4.954191762
14	C14	-0.908248474	0.652477598	4.534995591
15	C15	-1.212378335	-0.758185299	1.360639906
16	C16	-3.216108245	-0.492833111	3.360397855
17	C17	1.076149241	-0.525369208	2.538128356
18	O18	-3.173864193	-1.324197819	4.573891628
19	C19	-2.277842850	1.252650754	4.882726443
20	C20	0.213372401	-0.109752723	1.288000408
21	C21	-2.085913065	-0.319056296	0.211060191
22	C22	-3.367234107	0.969609141	3.824855187
23	C23	0.881478059	-0.640355788	-0.067287161
24	C24	-1.447203338	-0.442363214	-1.165095899
25	C25	0.048048034	-0.087111258	-1.268051633
26	C26	2.328629197	-0.114153487	-0.207775495
27	C27	0.641053293	-0.328854562	-2.702632496
28	C28	2.935238292	-0.315018610	-1.598657507
29	C29	2.063293217	0.313570742	-2.688676116
30	C30	-0.223116015	0.460667642	-3.715084669
31	C31	2.781258917	0.482231181	-4.061491416
32	C32	0.430367567	0.613478592	-5.088475225
33	C33	1.815037080	1.245481459	-5.004896052
34	H34	-2.078309397	-2.045219305	2.717923710
35	H35	-1.013250714	-1.794503122	1.068332418
36	H36	0.101662949	1.003023032	-1.170802739
37	H37	1.884998138	1.352459239	-2.361360648
38	H38	-0.612239353	1.788182727	0.500819856

39	H39	1.057432428	1.908947558	1.066213583
40	H40	-0.261117859	1.818152621	2.206987111
41	H41	-2.299772234	1.912732876	2.159804846
42	H42	-4.025571523	1.726550123	1.890142512
43	H43	-3.432108152	2.969067241	3.013539268
44	H44	-4.848493359	2.127841507	4.915149901
45	H45	-4.895032841	0.390209164	5.272722416
46	H46	-5.547096931	1.002583947	3.738630025
47	H47	1.778482641	-2.556148295	-0.653074349
48	H48	0.049184996	-2.673666779	-0.429228671
49	H49	1.119450645	-2.565507291	0.967154629
50	H50	-0.260446840	-2.326400264	-2.727465241
51	H51	1.499069866	-2.371346257	-2.774326974
52	H52	0.583153352	-1.924786283	-4.201576809
53	H53	3.804328669	2.251389947	-3.266988645
54	H54	4.361883382	1.722409444	-4.864240448
55	H55	4.856884792	0.831347329	-3.418066105
56	H56	3.907007291	-0.624046976	-5.551063307
57	H57	2.431358850	-1.466256019	-5.056285669
58	H58	3.833893060	-1.428849442	-3.988709294
59	H59	2.349296865	0.595710317	-6.753660350
60	H60	1.029284632	-1.370651426	4.537110464
61	H61	0.274438245	-2.397564729	3.326373170
62	H62	-0.381193717	1.299863469	3.837818644
63	H63	-0.288890797	0.614294305	5.439651072
64	H64	-4.088772604	-0.817017929	2.793233324
65	H65	1.478419650	0.388778518	2.988126309
66	H66	1.955382627	-1.090180418	2.220143023
67	H67	-2.180148022	2.334737577	5.026350628
68	H68	-2.610817026	0.846692396	5.844231780
69	H69	-2.049943793	0.164611821	-1.843454081
70	H70	-1.617978057	-1.488422454	-1.457811428
71	H71	2.362513516	0.959039326	0.006122586
72	H72	2.975638448	-0.596334772	0.533298369
73	H73	3.928364373	0.143640364	-1.598675026
74	H74	3.099759825	-1.379897860	-1.800037490
75	H75	-0.420259351	1.463873405	-3.309855005
76	H76	-1.198189587	-0.023465740	-3.839927428
77	H77	-0.197988951	1.238898737	-5.731486360
78	H78	0.503465018	-0.361605726	-5.590148426
79	H79	1.701659411	2.258865475	-4.596745349
80	O80	-3.255229801	-0.015896569	0.327523661

---

5 lowest energy conformers were considered for Boltzmann average to contribute to the ^{13}C NMR data

III.27. Cartesian coordinates of the global minimum conformer of 3β-hydroxy-12-oxo-13Hα-olean-28(19β)-olide (13b)

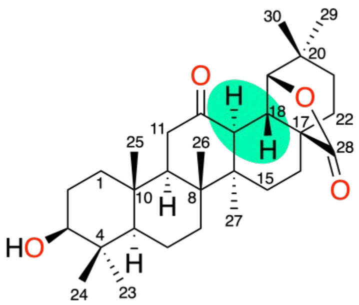

no	δ_{calc}	δ_{exp}	diff	IUPAC no	
C24	38.5	38.5	0.0	1	
C32	27.4	27.8	-0.4	2	
C33	77.2	77.9	-0.7	3	
C31	38.6	39.4	-0.8	4	
C29	54.3	55.6	-1.3	5	
C28	19.3	18.4	0.9	6	
C26	33.4	34.8	-1.4	7	
C23	41.2	41.7	-0.5	8	
C25	50.3	49.8	0.5	9	
C27	37.7	38.3	-0.6	10	
C30	37.7	38.5	-0.8	11	
C1	216.4	214.9	1.5	12	
C15	54.4	54.4	0.0	13	
C20	43.9	44.8	-0.9	14	
C17	26.7	26.1	0.6	15	
C12	26.4	26.0	0.4	16	
C10	41.9	42.0	-0.1	17	
C11	45.5	45.8	-0.3	18	
C16	86.2	87.2	-1	19	
C22	32.1	32.4	-0.3	20	
C19	32.1	33.6	-1.5	21	
C14	27.5	26.8	0.7	22	
C7	27.8	28.6	-0.8	23	
C8	16.4	39.4		24	**
C6	18.4	17.7	0.7	25	*
C5	17.0	15.9	1.1	26	*
C2	25.6	25.8	-0.2	27	
C13	179.0	180.1	-1.1	28	
C3	22.9	22.7	0.2	29	*
C4	29.6	30.5	-0.9	30	*
RMSD		0.8			
Max Abs		1.6			

** The chemical shift assigned to C-24 (non-IUPAC) (39.4 ppm) must be a typographical error and has therefore been excluded from the calculation of RMSD and Max Abs.

mol2 coordinates for lowest energy conformer

1	O1	1.771209778	2.728005550	5.307574787
2	C2	-1.352041105	-0.950994934	1.698676258
3	C3	3.632023821	-1.871908903	2.826147698
4	C4	5.120467077	-0.419459284	4.183757653
5	C5	0.301934233	1.890832545	-0.509119718
6	C6	0.181154542	0.563575899	-3.357571159
7	C7	-4.490878865	0.221831300	-3.855623796
8	C8	-2.481330599	1.065211410	-5.037464132

9	O9	-3.369233145	-1.584811203	-5.881576748
10	C10	1.089075661	0.851149465	3.861053950
11	C11	1.769527219	0.740549231	2.488613269
12	C12	-0.392280874	1.230464889	3.762667522
13	C13	2.014165261	1.897698875	4.473619499
14	C14	1.285155107	-0.456038841	4.672302789
15	C15	1.040710391	-0.216224916	1.557147526
16	C16	3.229230339	0.648091659	2.959038599
17	C17	-0.845212175	1.407883435	2.307327621
18	O18	3.242961470	1.754052027	3.917515228
19	C19	2.756513881	-0.790962762	4.950682155
20	C20	-0.458646878	0.255082808	1.333825263
21	C21	1.784639040	-0.493114487	0.263677895
22	C22	3.671604175	-0.622140551	3.717070177
23	C23	-0.657416468	0.725445170	-0.174270288
24	C24	0.989235419	-1.124777633	-0.857643330
25	C25	-0.419051704	-0.517081952	-1.081059419
26	C26	-2.096430243	1.236794041	-0.406395670
27	C27	-0.782036826	-0.376070475	-2.596176749
28	C28	-2.476905533	1.382247265	-1.883389150
29	C29	-2.275448945	0.068175702	-2.644287182
30	C30	-0.693128660	-1.789061668	-3.217711423
31	C31	-2.976279721	0.009791002	-4.034486485
32	C32	-1.327431190	-1.881544237	-4.604210427
33	C33	-2.782577168	-1.422576088	-4.601065647
34	H34	1.714517457	1.726658462	2.007656079
35	H35	0.984019317	-1.205773782	2.027934825
36	H36	-1.144856003	-1.256683461	-0.729106153
37	H37	-2.805893185	-0.698070006	-2.052446952
38	H38	-1.052043790	-1.870137880	1.186375035
39	H39	-2.409150915	-0.774179605	1.482039036
40	H40	-1.272813959	-1.153456302	2.771492113
41	H41	2.613239552	-2.214840217	2.621399274
42	H42	4.128209898	-1.694495058	1.867542582
43	H43	4.149289558	-2.692983088	3.336177899
44	H44	5.432849346	-1.256415944	4.818852024
45	H45	5.224552361	0.505638285	4.756663370
46	H46	5.803193730	-0.374115496	3.327031210
47	H47	0.053595194	2.334965865	-1.472405533
48	H48	1.354185500	1.605111710	-0.561219050
49	H49	0.221493948	2.693745476	0.226065851
50	H50	1.163917044	0.607058875	-2.879449808
51	H51	-0.187467472	1.589106940	-3.426567877
52	H52	0.349331380	0.220455627	-4.381423257
53	H53	-4.879403862	-0.366209936	-3.015299427
54	H54	-5.010575530	-0.098520028	-4.762947879
55	H55	-4.740751856	1.272696963	-3.678625469
56	H56	-3.147178597	1.093476512	-5.906918417
57	H57	-1.464893751	0.884168664	-5.396724247
58	H58	-2.498832111	2.065247762	-4.592767274
59	H59	-2.825701951	-1.097322308	-6.511151354
60	H60	-0.995646678	0.457322414	4.250802734
61	H61	-0.576661358	2.154736660	4.320243765
62	H62	0.793704876	-1.275558239	4.132360128
63	H63	0.750669172	-0.350598214	5.623263215
64	H64	3.941706532	0.905406099	2.176860579

65	H65	-1.935545075	1.511674325	2.292881222
66	H66	-0.453651332	2.362822336	1.948153042
67	H67	2.841283794	-1.814883878	5.333406838
68	H68	3.118732831	-0.135362558	5.751538189
69	H69	0.895926153	-2.191899954	-0.612911599
70	H70	1.623086822	-1.072412989	-1.745582181
71	H71	-2.817450296	0.548939138	0.049765909
72	H72	-2.227176682	2.201568269	0.096061388
73	H73	-3.526043142	1.689849026	-1.930796881
74	H74	-1.906595473	2.193754860	-2.350139278
75	H75	-1.207901517	-2.499761048	-2.554718892
76	H76	0.351610726	-2.116831234	-3.273229811
77	H77	-1.291443133	-2.914729659	-4.965800601
78	H78	-0.757317396	-1.284456502	-5.329710768
79	H79	-3.351271994	-2.103515813	-3.953487421
80	O80	2.973874551	-0.271305411	0.140217258

---

6 lowest energy conformers were considered for Boltzmann average to contribute to the ^{13}C NMR data

III.28. Cartesian coordinates of the global minimum conformer of 3β,6β-dihydroxy-12-oxo-13Hβ-olean-28(19β)-olide (14a)

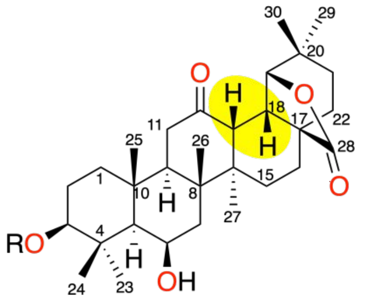

no	δ_{calc}	δ_{exp}	diff	IUPAC no	
C30	38.7	40.3	-1.6	1	
C32	29.1	27.2	1.9	2	
C33	77.3	79.0	-1.7	3	
C31	39.6	39.7	-0.1	4	
C29	56.1	55.8	0.3	5	
C28	70.0	68.4	1.6	6	
C26	40.8	42.4	-1.6	7	
C23	42.8	40.6	-2.2	8	*
C25	51.1	49.8	1.3	9	
C27	37.2	37.6	-0.4	10	
C24	37.8	38.0	-0.2	11	
C1	209.1	214.7	-5.6	12	
C15	51.1	54.1	-3.0	13	
C20	44.7	45.1	0.4	14	*
C17	29.0	26.0	3.0	15	
C12	30.6	26.8	3.8	16	
C10	40.8	42.0	-1.2	17	
C11	43.4	45.8	-2.4	18	
C16	88.1	88.0	0.1	19	
C22	30.9	32.4	-1.5	20	
C19	31.6	33.4	-1.8	21	
C14	24.5	25.4	-0.9	22	
C7	27.6	27.8	-0.2	23	

C8	17.7	17.0	0.7	24	
C6	18.3	17.1	1.2	25	
C5	18.0	18.9	-0.9	26	
C2	25.4	26.2	-0.8	27	
C13	178.3	180.5	-2.2	28	
C3	26.0	22.8	3.2	29	*
C4	29.7	30.4	0.7	30	*
RMSD		2.0			
Max Abs		5.6			

mol2 coordinates for lowest energy conformer

1	O1	-1.685173215	1.470009370	6.142168913
2	C2	1.545198203	0.047424548	1.140678116
3	C3	1.967370614	3.453401183	2.493156961
4	C4	1.229338904	4.849148071	4.412963199
5	C5	-2.114777370	-1.046735395	0.159659957
6	C6	-2.063527360	-0.672413459	-2.927688893
7	C7	1.179778952	-3.924005343	-4.025998336
8	C8	-1.082359013	-3.175639245	-4.705879563
9	O9	1.001143680	-2.253638265	-6.355564366
10	C10	-0.463624382	0.966560881	4.054234825
11	C11	-0.819325115	1.856392863	2.855668575
12	C12	-0.967772315	-0.438920069	3.780743750
13	C13	-1.164626130	1.809355914	5.114414757
14	C14	1.026726690	0.985365149	4.480074726
15	C15	-0.702469899	1.199019324	1.456178794
16	C16	-0.381133630	3.213426503	3.436882329
17	C17	-0.209919137	-1.071018842	2.588070888
18	O18	-1.084737500	3.113135434	4.725308430
19	C19	1.569158423	2.392805672	4.769695867
20	C20	0.028093853	-0.179380886	1.311990359
21	C21	-0.429337340	2.100322180	0.277037482
22	C22	1.105475700	3.465015788	3.758249965
23	C23	-0.583316524	-0.878422443	0.005697792
24	C24	-0.629264394	1.456384156	-1.087319836
25	C25	-0.198124978	-0.015060711	-1.234507893
26	C26	0.039210262	-2.277527381	-0.190450643
27	C27	-0.546342643	-0.619583490	-2.642896251
28	C28	-0.185986016	-2.944335994	-1.556012736
29	C29	0.173406123	-2.004058064	-2.714461253
30	C30	0.112990328	0.296104420	-3.705437824
31	C31	0.254906395	-2.695338943	-4.115340410
32	C32	0.209491230	-0.335524934	-5.092969864
33	C33	0.930607380	-1.677871676	-5.062153198
34	H34	-1.907884686	1.972167105	2.939201249
35	H35	-1.745998467	0.931835297	1.260947132
36	H36	0.897970429	-0.003797124	-1.244703170
37	H37	1.227530150	-1.758490398	-2.495936491
38	H38	1.986839903	0.448909139	2.048758765
39	H39	1.784866615	0.760738673	0.347498502
40	H40	2.077658944	-0.882718597	0.925073333
41	H41	1.961842582	2.483940468	1.993811244
42	H42	1.615096344	4.190932069	1.768410190
43	H43	3.004530849	3.690818452	2.757371861
44	H44	2.257616104	5.015862400	4.753455711
45	H45	0.563558225	4.938373591	5.275779423
46	H46	0.979229867	5.642608470	3.699686427
47	H47	-2.473807758	-1.850807864	-0.479882221
48	H48	-2.680647566	-0.150808101	-0.109608942

49	H49	-2.383857883	-1.304427400	1.189726227
50	H50	-2.585985283	0.161491152	-2.449375605
51	H51	-2.519053295	-1.602008101	-2.591295479
52	H52	-2.263748232	-0.579641118	-3.997345694
53	H53	2.108620533	-3.694200104	-3.487894432
54	H54	1.445003557	-4.252772587	-5.033811258
55	H55	0.686274906	-4.759953040	-3.520192049
56	H56	-0.887605857	-3.727919431	-5.629032208
57	H57	-1.761176969	-2.358532039	-4.954343539
58	H58	-1.593725011	-3.829083425	-3.997780385
59	H59	1.438140298	-1.619187464	-6.935183380
60	H60	-0.856686135	-1.081939901	4.660386285
61	H61	-2.042533973	-0.392825162	3.559834242
62	H62	1.639047947	0.492583707	3.728752672
63	H63	1.110957081	0.372678617	5.385814839
64	H64	-0.818355041	4.062537533	2.911477364
65	H65	0.765979876	-1.418824998	2.943816657
66	H66	-0.750003356	-1.981937684	2.318980568
67	H67	2.664691275	2.367182081	4.798503545
68	H68	1.245834377	2.697086972	5.771572841
69	H69	-0.120544328	2.096908704	-1.810258049
70	H70	-1.704653399	1.563783181	-1.289261137
71	H71	1.124673678	-2.226126367	-0.060637325
72	H72	-0.322156808	-2.967106451	0.583682772
73	H73	0.515535439	-3.789173461	-1.594152945
74	H74	1.127633422	0.560419157	-3.371713589
75	H75	-0.445173558	1.235468079	-3.784503207
76	H76	0.750650061	0.351089349	-5.758446437
77	H77	-0.781178829	-0.474502227	-5.539413822
78	H78	1.953830871	-1.508632576	-4.677283578
79	O79	-1.505926575	-3.459370788	-1.712096742
80	H80	-1.645213285	-4.115080355	-1.018621994
81	O81	-0.198776810	3.288056251	0.374037213

---

2 lowest energy conformers were considered for Boltzmann average to contribute to the ^{13}C NMR data

III.29. Cartesian coordinates of the global minimum conformer of 3β,6β-dihydroxy-12-oxo-13Hα-olean-28(19β)-olide (14b)

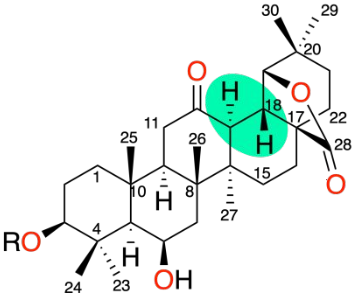

no	δ_{calc}	δ_{exp}	diff	IUPAC no	
C30	39.3	40.3	-1.0	1	
C32	27.5	27.2	0.3	2	
C33	77.8	79.0	-1.2	3	
C31	39.6	39.7	-0.1	4	
C29	55.3	55.8	-0.5	5	
C28	69.8	68.4	1.4	6	
C26	41.8	42.4	-0.6	7	
C23	40.7	40.6	0.1	8	*

C25	51.1	49.8	1.3	9	
C27	37.8	37.6	0.2	10	
C24	38.5	38.0	0.5	11	
C1	216.2	214.7	1.5	12	
C15	54.2	54.1	0.1	13	
C20	43.9	45.1	-1.2	14	*
C17	26.9	26.0	0.9	15	
C12	26.4	25.4	1.0	16	*
C10	42.0	42.0	0.0	17	
C11	45.6	45.8	-0.2	18	
C16	86.3	88.0	-1.7	19	
C22	32.1	32.4	-0.3	20	
C19	32.1	33.4	-1.3	21	
C14	27.4	26.8	0.6	22	*
C7	27.4	27.8	-0.4	23	
C8	17.3	17.0	0.3	24	
C6	18.8	17.1	1.7	25	
C5	17.6	18.9	-1.3	26	
C2	25.6	26.2	-0.6	27	
C13	179.1	180.5	-1.4	28	
C4	29.4	30.4	-1.0	29	
C3	22.8	22.8	0.0	30	
RMSD		0.9			
Max Abs		1.7			

mol2 coordinates for lowest energy conformer

1	O1	1.008892205	2.015230576	5.793252743
2	C2	-1.058082970	-1.626958281	1.450264468
3	C3	3.993328007	-1.473473709	2.681995750
4	C4	5.065225091	0.052765603	4.325162109
5	C5	-0.123020058	1.892862253	-0.116018472
6	C6	0.163824229	1.139795734	-3.153655983
7	C7	-4.317799162	0.008132156	-4.037536001
8	C8	-2.460079711	1.375301469	-4.940824058
9	O9	-2.811421590	-1.219282200	-6.231562942
10	C10	0.842141332	0.303136568	4.026615814
11	C11	1.534324144	0.609518396	2.689453762
12	C12	-0.686359310	0.305746900	3.920896945
13	C13	1.462794747	1.428384371	4.847894853
14	C14	1.364969225	-1.031181218	4.619032793
15	C15	1.073811173	-0.318760946	1.575734745
16	C16	2.966583678	0.805645258	3.209551790
17	C17	-1.164333565	0.628250833	2.499459923
18	O18	2.690250921	1.697474392	4.336941840
19	C19	2.870654385	-1.024388025	4.912563055
20	C20	-0.492872078	-0.192276702	1.358195201
21	C21	1.869867846	-0.186628325	0.289327718
22	C22	3.716067369	-0.423997349	3.767485397
23	C23	-0.783669051	0.496307253	-0.048637813
24	C24	1.248200461	-0.782612858	-0.954767214
25	C25	-0.255628228	-0.459467898	-1.154088662
26	C26	-2.301225665	0.673047046	-0.259200067
27	C27	-0.605507368	-0.098796053	-2.635916810
28	C28	-2.753891762	1.002804963	-1.689718353
29	C29	-2.158947277	0.022516230	-2.711142857
30	C30	-0.209400567	-1.329685329	-3.490172324

31	C31	-2.785356740	0.100838033	-4.140789479
32	C32	-0.780690807	-1.307782460	-4.906310677
33	C33	-2.299027562	-1.157322185	-4.911733511
34	H34	1.232162647	1.619114382	2.380446157
35	H35	1.259489906	-1.358661598	1.872799619
36	H36	-0.806029212	-1.392702321	-0.994727636
37	H37	-2.516081035	-0.952142877	-2.334762453
38	H38	-0.536594336	-2.328635191	0.792012025
39	H39	-2.126600599	-1.680187407	1.221220103
40	H40	-0.930537352	-2.003388786	2.470758634
41	H41	3.094358525	-2.018225877	2.380103636
42	H42	4.428008042	-1.018275274	1.788609706
43	H43	4.701019705	-2.214507616	3.070393834
44	H44	5.577148534	-0.772033666	4.834417740
45	H45	4.930336660	0.868127901	5.041282242
46	H46	5.717415284	0.406332261	3.518094127
47	H47	-0.444738059	2.418138621	-1.011935907
48	H48	0.968435969	1.866263132	-0.125975711
49	H49	-0.420343472	2.513831118	0.733382760
50	H50	1.103136978	1.282402890	-2.611659359
51	H51	-0.419338887	2.054615972	-3.061815701
52	H52	0.432283212	1.027726293	-4.207598379
53	H53	-4.629404457	-0.791548478	-3.353363270
54	H54	-4.732111969	-0.214963720	-5.024612216
55	H55	-4.756457890	0.949330345	-3.690262488
56	H56	-3.080805123	1.406425246	-5.844057003
57	H57	-1.413576812	1.439038294	-5.249099460
58	H58	-2.683074466	2.264800071	-4.349871361
59	H59	-2.365224314	-0.535182830	-6.746449651
60	H60	-1.071346873	-0.672105695	4.231087215
61	H61	-1.105351722	1.032811665	4.624726045
62	H62	1.106759751	-1.842273137	3.926583636
63	H63	0.815347069	-1.232206373	5.545526743
64	H64	3.594126449	1.365954205	2.517966005
65	H65	-2.245265128	0.456929403	2.450655423
66	H66	-1.026350876	1.699238030	2.332775661
67	H67	3.214051126	-2.042859288	5.128262134
68	H68	3.046944429	-0.443151600	5.825196675
69	H69	1.384529462	-1.870171474	-0.880335136
70	H70	1.867955576	-0.454695424	-1.791317065
71	H71	-2.827639200	-0.246452270	0.020258789
72	H72	-2.684591602	1.453842324	0.411701815
73	H73	-3.844247847	0.863864266	-1.700408978
74	H74	-0.569173320	-2.240324860	-2.989053996
75	H75	0.882062712	-1.415056298	-3.542740297
76	H76	-0.521918378	-2.235462497	-5.427862818
77	H77	-0.330162904	-0.493307560	-5.489665808
78	H78	-2.732001485	-2.034679498	-4.411957077
79	O79	-2.473990471	2.348299333	-2.064731887
80	H80	-2.862473384	2.924561583	-1.395492023
81	O81	2.976237796	0.316409789	0.259741277

---

10 lowest energy conformers were considered for Boltzmann average to contribute to the ^{13}C NMR data
